# Design, synthesis and optimization of TarO inhibitors as multifunctional antibiotics against Methicillin-resistant *Staphylococcus aureus*

**DOI:** 10.1038/s44259-025-00098-z

**Published:** 2025-04-12

**Authors:** Yuanchen Zhong, Feifei Chen, Dianyan Chen, Qian He, Xiaofei Zhang, Lefu Lan, Chunhao Yang

**Affiliations:** 1 School of Pharmaceutical Science and Technology, Hangzhou Institute for Advanced Study, University of Chinese Academy of Sciences, 310024 Hangzhou, China; 2 State Key Laboratory of Drug Research, Shanghai Institute of Materia Medica, Chinese Academy of Sciences, 555 Zuchongzhi Road, 201203 Shanghai, China; 3 University of Chinese Academy of Sciences, 100049 Beijing, China

**Keywords:** Drug discovery, Medicinal chemistry, Target identification

## Abstract

UDP-*N*-acetylglucosamine-undecaprenyl-phosphate *N*-acetylglucosaminephosphotransferase (TarO) has been found to simultaneously contribute to β-lactam resistance and virulence of Methicillin-resistant *Staphylococcus aureus* (MRSA). However, optimization of hit compounds targeting TarO has been hindered due to their high lipophilicity and the poor correlation between the enzyme activity inhibition and β-lactam sensitization. In this study, 31 analogues of Tarocin A were designed, synthesized and evaluated by a luminescence-based reporter preliminary screening. In the subsequent β-lactams synergy test, a good correlation was observed between the results obtained from these two methods. Finally, analog **18a** with more potential against TarO and an improved hydrophilicity (clogP = 3.2) was obtained. Compared with Tarocin A, **18a** shows stronger β-lactam sensitizing and anti-biofilm activities in vitro, as well as potent anti-virulence and synergistic potency with imipenem in vivo. These results suggest that TarO is a promising target for combating MRSA, and **18a** can serve as a lead molecule.

## Introduction

Methicillin-resistant *Staphylococcus aureus* (MRSA) is one of the most significant pathogens in both hospital and community settings^1,2^. Since its isolation in the 1960s, it has posed a serious threat to human and has become a global public health concern^3–5^. Based on the route of MRSA infection, it can be classified into healthcare associate MRSA (HA-MRSA) and community associate MRSA (CA-MRSA)^3^. Besides the widespread resistance to β-lactams, many CA-MRSA strains also exhibit strong virulence, making them more likely to cause serious infections^6–8^. Due to the increasing resistance of MRSA strains and sluggish progress in the development of new antibiotics, there is an urgent need for exploring new antimicrobial strategies^9,10^. Among them, the development of β-lactam sensitizers and anti-virulence agents holds significant promise for future development of anti-bacterial drugs^8,11–13^.

Wall teichoic acid (WTA) is an anionic glycophosphate polymer covalently attached to peptidoglycan of bacterial cell^14^. It is one of the main components of the cell wall for gram-positive bacteria, and plays critical roles in drug resistance and virulence of MRSA^12,15^. WTA can serve as a scaffold for the localization of penicillin-binding proteins (PBPs), including PBP2, PBP2a and PBP4, thereby influencing peptidoglycan cross-linking and resistance to β-lactams^15–18^. Additionally, WTA modulates the expression of major virulence factors such as phenol-soluble modulins (PSMs) and staphylococcal protein A (SpA) by interfering with the function of the two-component signal transduction systems, such as AgrCA, VraSR, and SaeSR^8,19–22^, thereby facilitating MRSA in colonization, immune evasion and spread in the host. Biofilms are communities of microorganisms in which cells adhere to a surface and are encased in a matrix composed of extracellular polymeric substances (EPS). Like many human pathogens, *S. aureus* also has the capability to form biofilms on implanted medical devices and host tissues, resulting in chronic or recurrent infections^23^. The absence of WTA reduces biofilm formation under both steady and flow conditions, likely by impairing the initial attachment to abiotic surfaces^12,24^.

The biosynthesis of WTA begins in the cytoplasm, where it is synthesized and subsequently translocated to the cell membrane surface^14^. TarO, an early-stage enzyme involved in WTA biosynthesis, catalyzes the transfer of *N*-acetylglucosamine-1-phosphate to a membrane-anchored undecaprenyl-phosphate carrier lipid^25–27^. Unlike late-stage enzymes in the WTA biosynthetic pathway, TarO is not essential for the survival of *S. aureus* but is critical for WTA biosynthesis^15,28–31^. Therefore, inhibiting TarO can prevent the biosynthesis of WTA, thereby reducing virulence, β-lactam resistance and biofilm formation capacity of MRSA^11,12,15,22,31^.

**Fig. 1:**
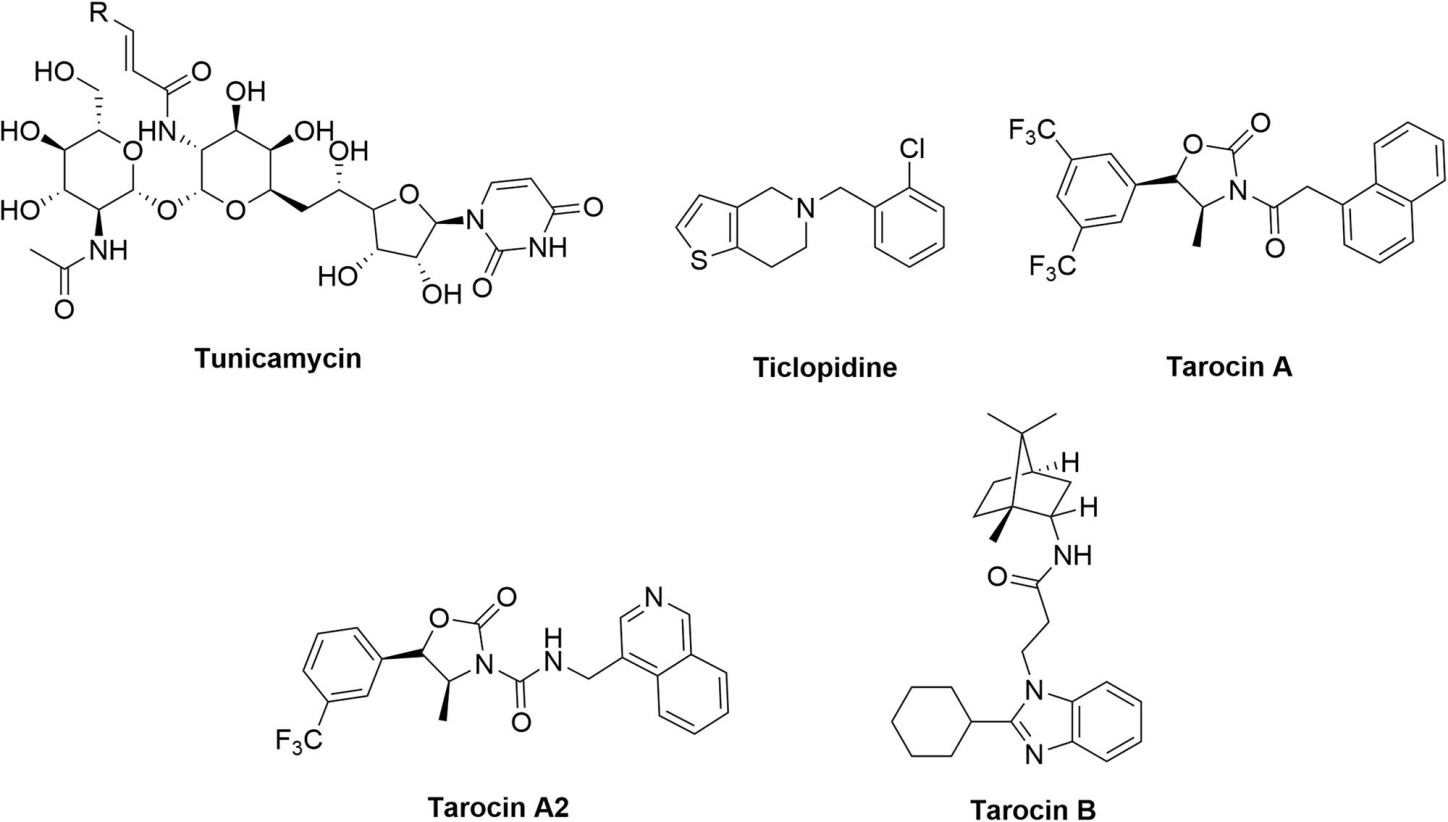
Compounds with TarO inhibitory activity.

Although TarO is a theoretically promising drug target^31^, reports on TarO inhibitors are limited and the development of such inhibitors faces significant challenges, resulting in slow progress. For example, as a multi-spanning membrane protein^11^, expression and purification of TarO are difficult and no successful reports have been documented thus far. This often leads to an indirect and potentially inaccurate evaluations on enzymatic activity inhibition in vitro. Meanwhile, only a few TarO inhibitors have been proven to be β-lactam sensitizers^11,31,32^, the expected anti-biofilm and anti-virulence activities remain to be validated. Tunicamycin, a natural product isolated from *Streptomyces lysosuperficus*, was the first compound identified with TarO inhibitory activity^32^. However, the significant cytotoxicity in eukaryotic cells has hindered its further development^11,33,34^. Subsequently, Ticlopidine and its analogues were reported to have low to moderate TarO inhibitory activity, but their metabolic stability issues were proved to be difficult to overcome^35^. In 2016, Lee et al. discovered two series of compounds, with oxazolidin-2-one and benzimidazole scaffold, respectively, exhibit TarO inhibitory activity through high-throughput screening (HTS)^11^. These compounds exhibit good TarO inhibitory activity and lower cytotoxicity compared to tunicamycin, indicating their potential for further optimization. Although the representative compound, Tarocin A and B showed good performance in TarO inhibition, two major issues remain unresolved. The first is the relatively high lipophilicity (clogP > 7.0) of these compounds and the second is the poor correlation between the enzyme inhibitory activity and sensitizing efficacy to β-lactams. Subsequently, series of modifications were attempted to improve the drug-like properties of these compounds^36–38^. The best performed compound Tarocin A2, which shows better water solubility (lower clogP) but attenuated TarO inhibiting activity compared with Tarocin A, is still not ideal and failed in further investigation^38^ (Fig. 1).

The existing method for determining the IC_50_ of TarO inhibitors is indirect, relying on measuring the inhibition of the product generated by cell membrane extracts containing TarO^11,31^. Although this approach allowed for a quantitative assessment of the inhibitors, there might be inaccuracies in the biochemical assay due to the unavoidable interference from other membrane proteins present in the complex mixtures during the reaction.

In a previous study, we found that targeting TarO rapidly triggers the expression of *vraX*^22^, a gene encoding an important immune evasion factor^39^. This was achieved *via* the VraSR two-component signal transduction system under cell wall stress stimulation^22,40^. Specifically, targeting TarO leads to the loss of WTA, causing the mislocalization of penicillin-binding protein 2 (PBP2) and thus blocks peptidoglycan synthesis. The cell wall stress resulting from the ineffective cell wall biosynthesis activates the VraSR system, and the corresponding response regulator VraR dimerizes^41^ and subsequently binds to the promoter region of *vraX* to upregulate its expression^22^. Consequently, a *lux*-based bioluminescence system was constructed by expressing the *lux* operon under the control of the *vraX* promoter (*vraX*_pro_-*lux*)^22^. This reporter system was used for the preliminary screening of new synthetic TarO inhibitors, considering that the expression of *vraX* will be induced once the activity of TarO is effectively hindered, thereby a lack of WTA. Considering this and also the high lipophilicity of the compounds may lead to significant differences in inhibitory activity between the cellular and enzymatic levels, we attempt to discover TarO inhibitors at the cellular level directly in preliminary screening, by using the *vraX*_pro_-*lux* bioluminescence reporter system.

With these concerns in mind, we attempted to further optimize the TarO inhibitors based on the previously discovered structure-activity relationship (SAR) of Tarocin A. Thus, 31 analogues of Tarocin A were designed, synthesized and preliminary evaluated *via* the reporter system. The promising hits were subsequently validated by a β-lactams synergy test, and a strong correlation was observed between the results from these two methods. In the end, a more potent and hydrophilic compound, designated **18a**, was obtained and demonstrated well β-lactam sensitization, anti-virulence and anti-biofilm activities against MRSA. This study provides further insights into the SAR of Tarocin A, and highlights the utility and convenience of the luminescence-based reporter system for the preliminary screening of TarO inhibitors.

## Results

### Design

**Fig. 2:**
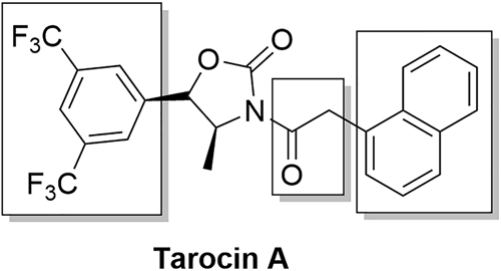
Illustration of the modification regions of Tarocin A.

Modification of Tarocin A is divided into three regions as shown in Fig. 2. By introducing heteroatoms or hydrophilic groups respectively to explore possible substitutable sites of compounds. Then, through advantageous structure splicing, attempts are made to obtain compounds that can improve the hydrophilicity while retain the good TarO inhibitory activity.

### Chemistry

**Scheme 1:**
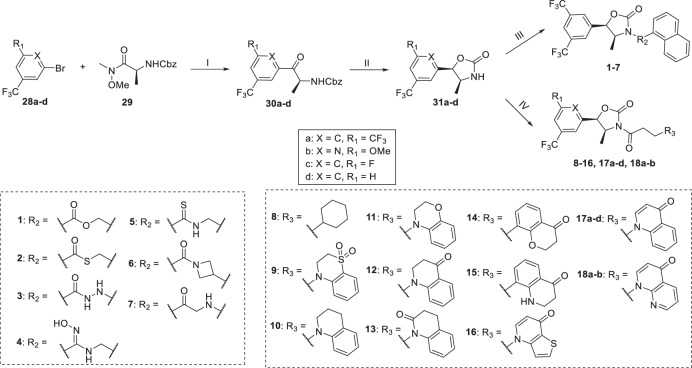
Reagents and conditions: (I) *i*-PrMgCl, THF, -10 °C to rt, 16 h; (II) Al(O-*i*-Pr)_3_, toluene, 2-propanol, 50 °C, 20 h, then KOH, H_2_O, rt, 10 h, 34–49% (2 steps); (III) acyl halides, *n*-BuLi, THF, −78 °C to rt, 2 h, then Et_3_N, nucleophiles, rt, overnight, 26–56%; (IV) carboxylic acids, CMPI, DMAP, Et_3_N, DMF, rt, overnight, 40–68%.

The synthesis of oxazolidine-2-one intermediates **31a-d** proceeded according to the reported route (Scheme 1)^38^. The aryl bromides **28a-d** reacted with Weinreb amide **29** under the action of *i*-PrMgCl to obtain ketones **30a-d**, which were then stereoselectively reduced under Meerwein-Ponndorf-Verley conditions and cyclized with KOH to form **31a-d**. Compounds **1–7** were obtained from **31a-d** reacted with the corresponding acyl halides in the presence of *n*-BuLi and treated with the corresponding nucleophiles. Compounds **8–16, 17a-d,** **18a-b** were synthesized by condensation of **31a-d** with corresponding carboxylic acids under basic conditions via condensation agent 2-chloro-1-methylpyridinium iodide (CMPI) and catalyst 4-dimethylaminopyridine (DMAP). Compound **15** were involved in the protection and deprotection of -Boc protecting group.

**Scheme 2:**
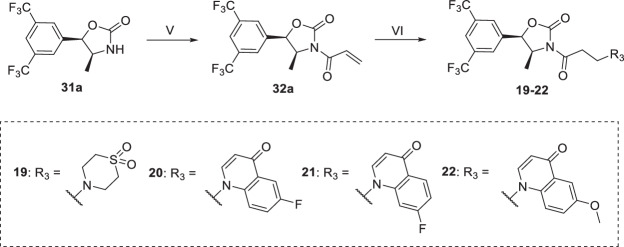
Reagents and conditions: (V) acrylic anhydride, LiCl, Et_3_N, THF, rt, 6 h, 54%; (VI) Michael donors, NaH (or Et_3_N for **19**), DMF, rt, 0.5 h, 20–32%.

The preparation of compounds **19–22** was carried out according to Scheme 2. **31a** reacted with acrylic anhydride under the action of LiCl and Et_3_N to give **32a**^42^. **32a** underwent aza-Michael addition with the corresponding Michael donors under the action of NaH or Et_3_N to obtain **19–22**.

**Scheme 3:**
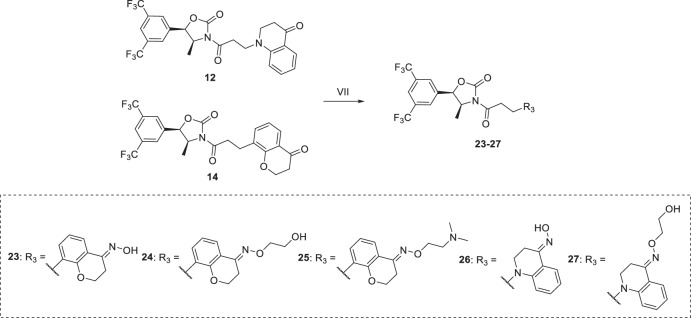
Reagents and conditions: (VII) hydroxylamines, AcOH, MeOH, rt, overnight, 52–85%.

As shown in Scheme 3, oximes **23–27** were obtained by condensation of compound **12** or **14** with corresponding hydroxylamines in the presence of acetic acid.

### Validation of the evaluation model with known TarO inhibitors

**Fig. 3:**
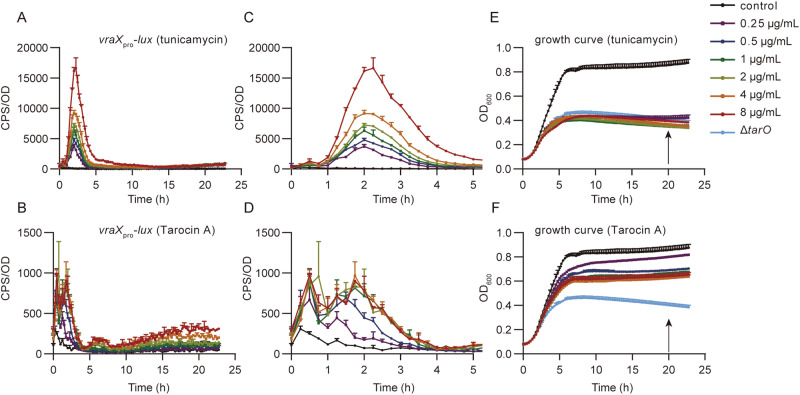
Expression of *vraX*_pro_*-lux* and the growth curve induced by tunicamycin and Tarocin A. Data from *n* = 2 biological replicates and reported as the mean ± SD. **A**, **B** Expression of *vraX*_*pro*_*-lux* induced by different concentrations of tunicamycin and Tarocin A, respectively. **C**, **D** Enlarged figure of (**A**) and (**B**) for the first five hours after induction. **E**, **F** The corresponding growth curve treated by tunicamycin or Tarocin A. Growth curve of a *tarO* null mutant of USA300 LAC strain was also illustrated as a positive control, and the black arrow indicated the time at which OD_600_ was recorded and used for IC_50_ calculation. In (**E**) and (**F**), data from WT control and the isogenic Δ*tarO* strain was derived from the same two biological replicates, respectively.

As expected, addition of tunicamycin or Tarocin A, the previously discovered TarO inhibitor, to MRSA USA300 LAC strain could induce the expression of *vraX*_pro_*-lux* during the exponential growth phase in a concentration-dependent manner (Fig. 3A–D). Meanwhile, compared to wild-type strain, it was reported that the *tarO* mutant exhibits enhanced autolysis in the late growth stage, resulting in a reduced cell density, reflected by the optical density at 600 nm (OD_600_)^22^. In fact, after entering the post-exponential growth phase, treating with tunicamycin or Tarocin A effectively reduced the bacterial growth density, there by a lower OD_600_ value (Fig. 3E, F). Based on these features, we presume that when an inhibitor acts on TarO, the plateau growth curve of the treated wild-type strain will be between the untreated wild-type strain and the *tarO* mutant, along with raised expression of *vraX*_pro_*-lux*. By choosing the twentieth hour (determined by multiple tests) as the sampling point during the plateau phase, relative inhibition rate of different concentrations of active compounds was obtained and used to plot a dose-response curve, and the IC_50_ was calculated.

### TarO inhibitors can achieve dual guarantees of low lipophilicity and high activity

To explore whether TarO inhibitors are able to maintain activity with a low lipophilicity, we modified Tarocin A regionally to test the tolerance range of each part. clogP was used as rough estimate of the lipophilicity of compounds.

Initially, attempts were made to modify the linker between the oxazolidine-2-one and the naphthalene (Table 1). As expected, a positive correlation between compound lipophilicity and activity was generally observed. The introduction of a sulfur atom with better lipophilicity resulted in compound **2** exhibiting potent TarO inhibitory activity, while the introduction of groups with more water-soluble led to varying degrees of decreased activity (compounds **3,** **4,** **5** and **7**), see Supplementary Fig. 1 and 2. Additionally, the flexibility of the alkyl chain is essential for TarO inhibitory activity, as replacement with azetidine (compound **6**) resulted in loss of activity.

**Table 1:** SAR Studies of linker


Compounds	R_2_	IC_50_ (μg/mL)^*a, b*^	clogP^*c*^
Tarocin A		4.67	7.1
Tarocin A2		10.41	4.6
**1**		0.08	7.6
**2**		0.05	8.1
**3**		n.a.	6.4
**4**		n.a.	7.0
**5**		4.60	7.2
**6**		n.a.	7.2
**7**		3.57	6.6

^*a*^IC_50_ is calculated based on the relative inhibition rate of compounds against the wild-type strain at different concentrations, dose–response curve was shown in Fig. 4 and Supplementary Fig. 2.

^*b*^n.a. means that IC_50_ cannot be calculated from the response curve within the test range.

^*c*^clogP data were calculated by software Chemdraw.

**Fig. 4:**
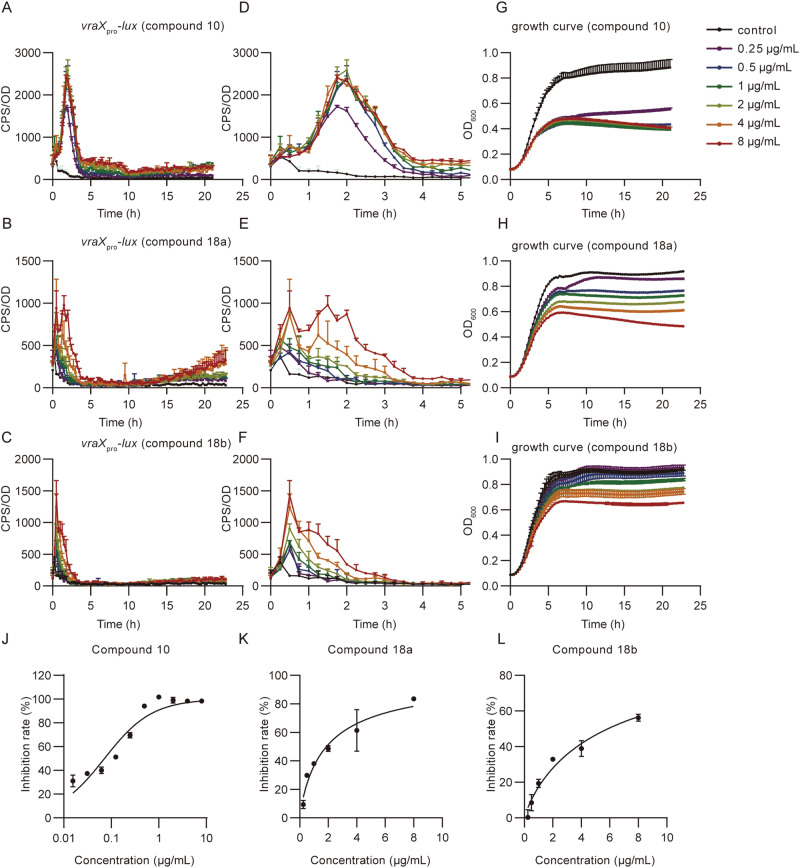
TarO inhibitory activity of compound 10, 18a and 18b. **A**–**C** Expression of *vraX*_*pro*_*-lux* induced by different concentrations of compound **10,** **18a** and **18b**, respectively. **D**–**F** Enlarged figure of (**A**–**C**) for the first five hours after induction. **G**–**I** Corresponding growth curve treated by compound **10,** **18a** or **18b**. **J**–**L** Doses–response curve generated by different concentrations of the three compounds and TarO inhibition rate, data was obtained from two biological replicates.

Due to the challenges associated with modifying the linker, we turned our attention to the naphthyl group (Table 2). First, we attempted to replace the aromatic ring with a saturated alkyl ring, but the resulting compound **8** was inactive, implying that there might be crucial π-π interactions in this region. Furthermore, when a strongly hydrophilic thiomorpholine-1,1-Dioxide was fused to the benzene ring, compound **9** exhibited potent inhibition activity comparable to Tarocin A while compound **19** without aromatic ring on the right part lost its activity. Therefore, we further explored the TarO inhibition activity with various benzene-fused cycloalkyl compounds containing hydrogen bond acceptors (compounds **10–15,** **17a**), among which, compounds **12,** **14**, and **17a** displayed increased activity with reduced clogP. Except highly lipophilic compound **2** (clogP = 7.6), Compound **10** exhibited most potent TarO inhibiting activity in vitro (Fig. 4 and Table 2), it was selected for further investigation to verify this assay. Since compound **17a** successfully lowered the clogP with potent activity, we continued to explore the modification at position 6/7 on the quinolin-4(1*H*)-one of **17a** (compounds **20–22**), as well as the introduction of more hydrophilic hydrogen bond acceptors at the carbonyl positions of compounds **12** and **14** to explore their effects on activity (compounds **23–27**). Unfortunately, these further substitution derivatives all led to decreased or lost activity (Supplementary Fig. 1 and 2), possibly due to the limited binding pocket space for the naphthalene ring. To further explore the chemical space of compound **17a**, we replaced the benzene ring with heterocycles (**16,** **18a**), and we were pleased to find that compared with Tarocin A and A2, compound **18a** has a much lower clogP, while also improved the inhibition activity to some extent (Fig. 4 and Table 2). Additionally, the water solubility of compound **18a** (20 μg/mL) was significantly improved compared to Tarocin A (almost insoluble in pure water).

**Table 2:** SAR Studies of naphthalene ring region


Compounds	R_3_	IC_50_ (μg/mL)^*a,b*^	clogP^*c*^
**8**		n.a.	7.4
**9**		3.48	5.3
**10**		0.07	7.1
**11**		1.00	6.5
**12**		2.60	6.2
**13**		n.a.	5.8
**14**		2.08	5.9
**15**		n.a.	6.3
**16**		n.a.	4.1
**17a**		2.09	4.5
**18a**		1.88	3.2
**19**		n.a.	3.6
**20**		n.a.	4.6
**21**		n.a.	4.6
**22**		n.a.	4.5
**23**		4.00	6.4
**24**		n.a.	6.0
**25**		n.a.	6.9
**26**		n.a.	6.6
**27**		n.a.	6.3

^*a*^IC_50_ is calculated based on the relative inhibition rate of compounds against the wild-type strain at different concentrations, dose–response curve was shown in Fig. 4 and Supplementary Fig. 2.

^*b*^n.a. means that IC_50_ cannot be calculated and given from the response curve within the test range.

^*c*^clogP data were calculated by software Chemdraw.

For the 3,5-bis(trifluoromethyl)phenyl segment, we attempted to replace it with several structures reported in the literature (compounds **17b-d,** **18b**)^38^. As shown in Table 3, the activity of the compounds decreased to varying degrees. Among them, 6-methoxy-4(trifluoromethyl)pyridine had a more balanced effect on the compound’s activity and clogP, ultimately resulting in compound **18b** with comparable activity to Tarocin A and a lower clogP of 1.7 (Fig. 4 and Table 3).

**Table 3:** SAR Studies of 3,5-difluoromethylbenzene region


Compounds	R_3_	R_4_	IC_50_ (μg/mL)^*a*^	clogP^*b*^
**17b**			10.65	3.0
**17c**			12.87	3.7
**17** **d**			13.40	3.6
**18b**			5.86	1.7

^*a*^ IC_50_ is calculated based on the relative inhibition rate of compounds against the wild-type strain at different concentrations (relative inhibition rate is defined as the ratio of the growth inhibition rate in the inhibitor-treated group to that in the *tarO* mutant group at 20 h).

^*b*^clogP data were calculated by software Chemdraw.

### Further validation of the TarO inhibitor

**Fig. 5:**
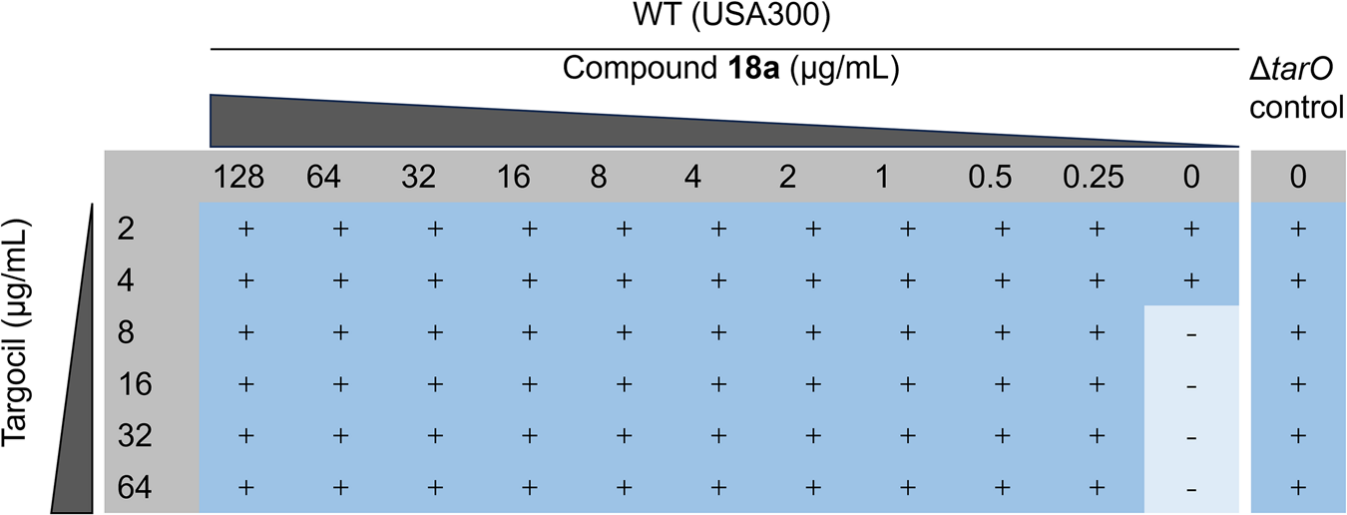
Chemical suppression results of compound 18a against Targocil. Wild-type strain (*S.aureus* USA300 LAC)was used in this test, and its isogenic *tarO* null mutant (Δ*tarO*) strain treated by Targocil were used as a positive control. + indicated growth and − indicated no growth by visual observation.

Since compounds that induce cell wall stress without targeting TarO may also trigger vraX_pro_-*lux* expression and inhibit the growth of *S.aureus*, two other experiments were performed to confirm the target of these new Tarocin A derivatives. First, a chemical suppression experiment of the growth inhibition by depleting late-state WTA biosynthesis was conducted. This strategy based on the previously paradoxical observation that while genes for late WTA synthesis steps are essential, they become dispensable in strains lacking early-step genes, such as *tarO* or *tarA* (encoding for N-acetylglucosaminyldiphospho-undecaprenol-N-acetyl-β-D-mannosaminyltransferase)^43^. The suppression test has been used to identify inhibitors of early steps for WTA biosynthesis, and several different scaffolds of compounds, including the TarO inhibitors, have been described^11,38,44^. Consistent with the previous report, Targocil could inhibit the growth of *S.aureus* with a MIC value about 8 μg/mL (Fig. 5) by targeting TarG, which involved in the late-step of WTA biosynthesis and thus plays an essential role in *S.aureus* survival^45^. As expected, Targocil did not exhibit a lethal effect on the *tarO* mutant (Fig. 5), since the early-step of WTA biosynthesis was disrupted. Surprisingly, compound **18a** could effectively alleviate the growth inhibition of Targocil on WT strain at a concentration as low as 0.25 μg/mL. This suggested that **18a** may target an early step of WTA biosynthesis.

**Fig. 6:**
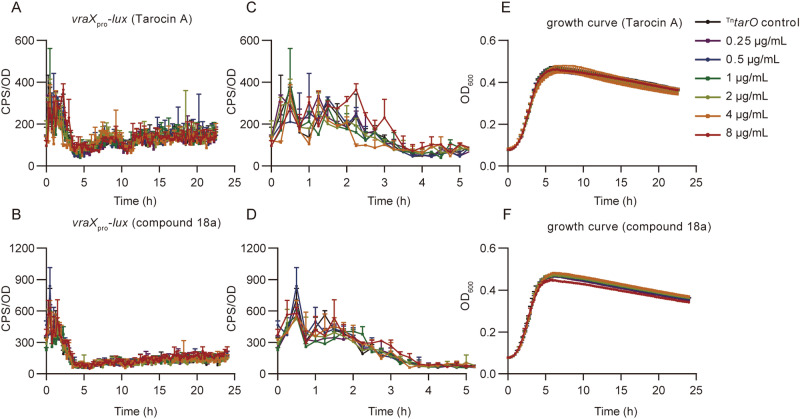
Expression of *vraX*_pro_*-lux* and the growth curve of *tarO* mutant induced by Tarocin A and compound 18a. **A**, **D** Expression of *vraX*_*pro*_*-lux* induced by different concentrations of Tarocin A and compound **18a**, respectively. **B**, **E** Enlarged figure of (**A**) and (**D**) for the first five hours after induction. **C**, **F** Corresponding growth curve treated by Tarocin A and compound **18a**. ^Tn^*tarO*, *S.aureus* JE2 harbors a *vraX*_pro_-*lux* reporter system with a transposon inserted in *tarO*, causing this gene disrupted. *S.aureus* JE2, a derivative of USA300 LAC strain after all three native plasmids were cured for facilitating genetic manipulation^50^.

Second, the *vraX*_pro_-*lux* induction and growth inhibition experiment were conducted using a *tarO* mutant, as we assumed that a compound with other targets may still change the growth characteristic and the *vraX*_pro_-*lux* expression pattern of the mutant, whereas a TarO inhibitor would not, as its target is absent in the mutant. Indeed, neither Tarocin A nor **18a** further changed the *vraX*_pro_-*lux* expression pattern as well as the growth characteristics of the mutant (Fig. 6).

### TarO inhibitors exhibit no eukaryotic cell toxicity

Excellent antibacterial adjuvants should avoid toxicity to both bacteria and eukaryotic cells in order to minimize bacterial survival pressure and maximize the safety. To assess whether the new derived TarO inhibitors have bacteriostatic potential toward *S.aureus*, minimum inhibitory concentration (MIC) of some representatives, including compounds **10,** **18a**, and **18b**, were tested against three MRSA strains (USA300 LAC, USA400 MW2 and HS663). As shown in Table 4, all compounds showed no anti-*S.aureus* activity, as MIC of these compounds were above 256 μg/mL. Furthermore, no significant eukaryotic cell toxicity was observed, as the IC_50_ of these compounds was above 20 μM for all three tested cell lines.

**Table 4:** In vitro antibacterial activity and eukaryotic toxicity of TarO inhibitors

Compounds	MIC (μg/mL)			IC_50_ (μM)^*a*^		
	USA300 LAC^*a*^	USA400 MW2^*b*^	HS663^*c*^	Capan-1	HCT-116	NCI-H1373
**10**	>256	>256	>256	>20	>20	>20
**18a**	>256	>256	>256	>20	>20	>20
**18b**	>256	>256	>256	>20	>20	>20
Tarocin A	>256	>256	>256	>20	>20	>20

^a^USA300 LAC: A representative MRSA (Community associated methicillin-resistant *Staphylococcus aureus*) isolate, which was first isolated in Los Angeles, Calif^48^.

^b^USA400 MW2: A prototype MRSA strain type endemic in the U.S. Midwest^48^.

^c^HS663: A ST239 MRSA isolated from China^49^.

### New TarO inhibitors can effectively increase the sensitivity of MRSA to β-lactams

To determine whether the compounds identified as positive hits in preliminary screening can enhance the sensitivity of MRSA to β-lactam antibiotics, compounds **10,** **18a**, and **18b** were evaluated for their synergistic inhibitory effects against various MRSA strains in combination with different β-lactam antibiotics (USA300 with oxacillin, USA400 with oxacillin, or HS663 with imipenem) using a checkerboard assay. As shown in Table 5, fractional inhibitory concentration index (FICI) of the compounds with each corresponding antibiotic was less than or close to 0.50, indicating their potential to sensitize MRSA to β-lactams, as expected. Notably, the minimum sensitization concentration of the compounds is positively correlated with the inhibition ability determined in the preliminary screening. In other words, the lower the IC_50_ value of the compounds, the lower the concentration was required for sensitizing β-lactam antibiotics (in general, **10** < **18a** < **18b** ≈ Tarocin A). However, consistent with the previous report^38^, there is no correlation between the maximum sensitization fold for β-lactams and the IC_50_ value of the compounds, as shown in Table 6). Overall, the order for the maximum sensitization fold was Tarocin A < **10** < **18b** < **18a**. This trend was further confirmed by testing additional compounds, with the strongest sensitization effect exceeding 1024-fold, see Supplementary Table 1.

**Table 5:** The minimum sensitization concentration of the new TarO inhibitors to β-lactams

Compounds	Strains/antibiotics	Minimum sensitization concentration (μg/mL)^a^	MIC (μg/mL)^b^ Individual/combined	Sensitization fold	FICI^*c*^
**Tarocin A**	USA300 LAC/ oxacillin	0.06	64 /32	2	<0.51
	USA400 MW2/oxacillin	0.06	16 / 8	2	<0.51
	HS663/imipenem	0.25	64 / 32	2	<0.51
**10**	USA300 LAC / oxacillin	0.03	64 / 8	8	<0.13
	USA400 MW2/oxacillin	0.03	16 / 2	8	<0.13
	HS663/imipenem	0.03	64 / 8	8	<0.13
**18a**	USA300 LAC / oxacillin	0.03	64 / 8	8	<0.13
	USA400 MW2/oxacillin	0.03	16 / 4	4	<0.26
	HS663/imipenem	0.03	64 / 32	2	<0.51
**18b**	USA300 LAC / oxacillin	0.25	64 / 16	4	<0.26
	USA400 MW2/oxacillin	0.06	16 / 8	2	<0.50
	HS663/imipenem	0.12	64 / 32	2	<0.51

^a^Test range: 0.03–32 μg/mL.

^b^MIC of β-lactams used alone or in combination with TarO inhibitors.

^c^FICI = (MIC_inhibitor_ in combination/MIC_inhibitor_ alone) + (MIC_antibiotic_ in combination/MIC_antibiotic_ alone).

**Table 6:** The maximum sensitization concentration of the new TarO inhibitors to β-lactams

Compounds	Strains/antibiotics	Maximum sensitization concentration (μg/mL)^a^	MIC (μg/mL)^b^ Individual/combined	Sensitization fold	FICI^c^
**Tarocin A**	USA300 LAC/oxacillin	0.5	64/16	4	<0.26
	USA400 MW2/oxacillin	0.5	16/1	16	<0.07
	HS663/imipenem	2	64/8	8	<0.14
**10**	USA300 LAC/oxacillin	0.06	64/4	16	<0.07
	USA400 MW2/oxacillin	0.12	16/1	16	<0.07
	HS663/imipenem	0.25	64/1	64	<0.02
**18a**	USA300 LAC/oxacillin	32	64/0.25	256	<0.13
	USA400 MW2/oxacillin	32	16/0.25	64	<0.13
	HS663/imipenem	4	64/8	8	<0.15
**18b**	USA300 LAC/oxacillin	32	64/4	16	<0.19
	USA400 MW2/oxacillin	32	16/0.5	32	<0.16
	HS663/imipenem	2	64/4	16	<0.08

^a^test range: 0.03–32 μg/mL.

^b^MIC of β-lactams used alone or in combination with TarO inhibitors.

^c^FICI = (MIC_inhibitor_ in combination) + (MIC_antibiotic_ in combination/MIC_antibiotic_ alone).

### Anti-biofilm formation of the new TarO inhibitors

**Fig. 7:**
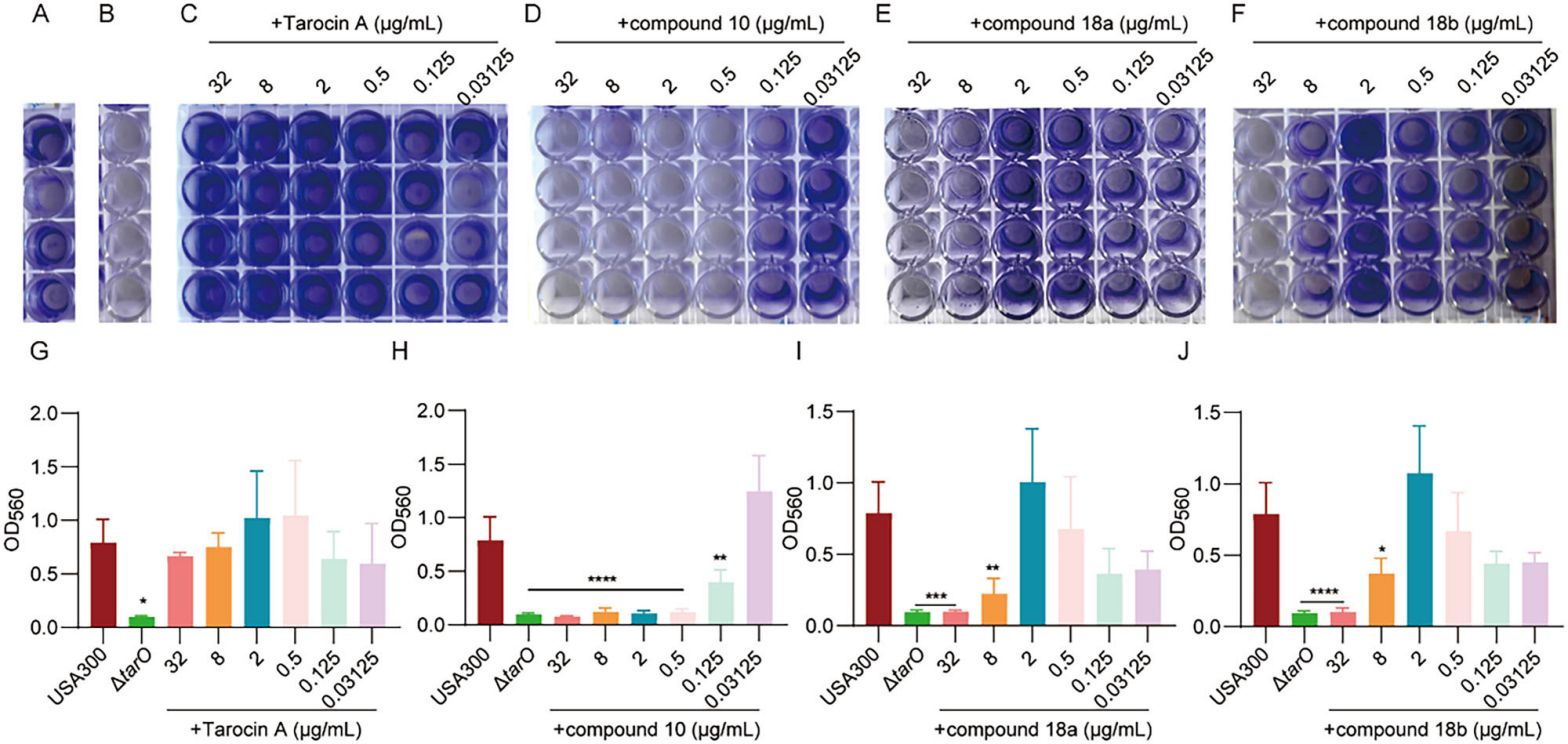
Anti-biofilm formation of the new TarO inhibitors. *S.aureus* USA300 LAC (**A**) and the its isogenic Δ*tarO* strain (**B**) treated by DMSO were used as negative or positive control, respectively. Biofilms were stained with crystal violet (**A**–**F**) and released by glacial acetic acid. Absorbance at 560 nm (*A*_560_) was recorded and used to quantify the biofilm (**G**–**J**). Each experiment was performed with four biological replicates, and data was represented by mean ± SD; *P < 0.05, **P < 0.01, ***P < 0.001, ****P < 0.0001, calculated by One-Way ANOVA with Dunnett test. In (**G**–**J**), data from WT and the isogenic Δ*tarO* strains was from the same four biological replicates.

As WTA was important for the initial adhere of *S.aureus* cells to the surface of abiotic surfaces and essential for a full formation of the biofilms^12^, we also explored the anti-biofilm formation potential of the new TarO inhibitors *via* the crystal staining method. As expected, knockout of *tarO* significantly impaired biofilm formation of the USA300 LAC strain (Fig. 7A, B). However, Tarocin A did not reduce static biofilm formation in USA300 LAC within the tested concentration range (0.03 to 32 μg/mL), whereas both compound **18a** and **18b** can significantly reduce the biofilm formation at a concentration as low as 8 μg/mL. Particularly, compound **10**, with the lowest IC_50_ value in preliminary screening, achieved over 90% and near a half inhibition of biofilm formation at a concentration of 0.5 or 0.125 μg/mL, respectively. These results suggest that blocking WTA biosynthesis by targeting TarO may be a promising strategy to combat biofilm formation.

### Compound 18a significantly reduce the virulence and increase sensitivity of MRSA to β-lactams

**Fig. 8:**
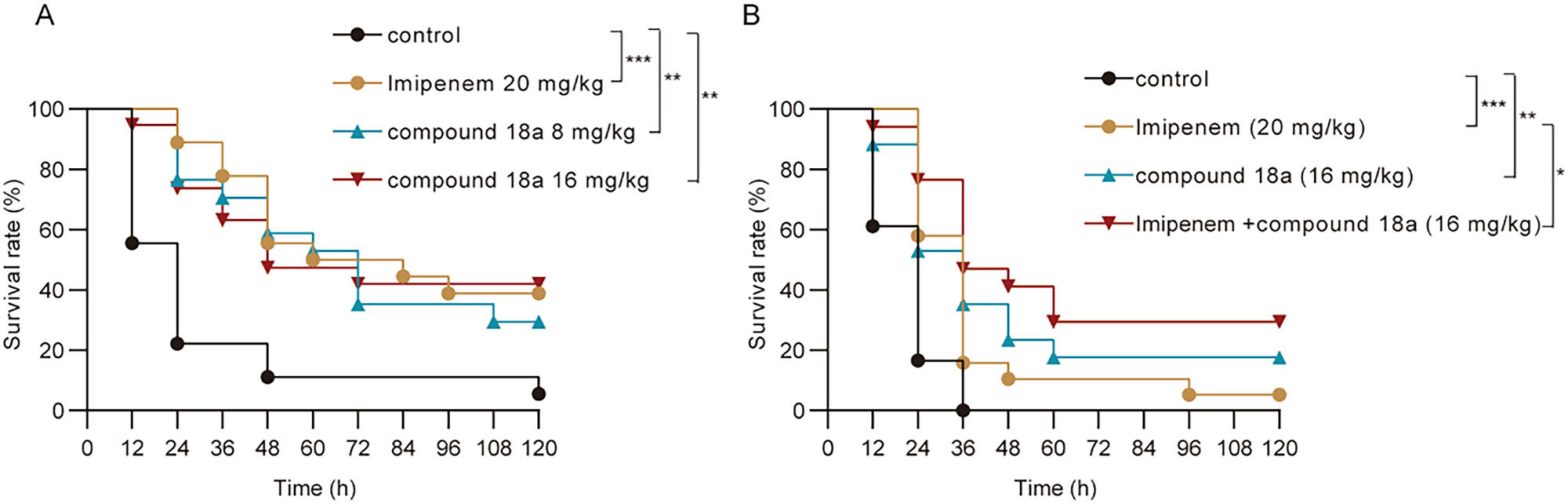
Survival curves of *Galleria mellonella* larvae infected with MRSA HS663 strain. **A** The impact of administering **18a** or imipenem alone on the survival rate of *Galleria mellonella* larvae infected; at a dose of 1.5 × 10^6^ CFU. MIC of imipenem against HS663 was 64 μg/mL. **B** Impact of administering **18a** or imipenem alone or in combination on the survival ability of *Galleria mellonella* larvae infected with HS663 at a dose of 2.0 × 10^6^ CFU. For each group, eighteen larvae were included and the survival/deaths of the larvae was observed d every 12 h for 5 days. *P < 0.05, **P < 0.01, ***P < 0.001, by log-rank test.

Based on the in vitro data, we further chose compound **18a**, as it showed an applaudably improved water solubility while still retained excellent inhibitory activity towards TarO, to evaluate its potential therapeutic effects in vivo using a *Galleria mellonella* larva infection model. As shown in Fig. 8A, a single administration of **18a** at 8 mg/kg and 16 mg/kg significantly extended the survival time of the *Galleria mellonella* larvae, which was comparable to 20 mg/kg of imipenem. At a higher infection dose, the extension of survival time for **18a** at 16 mg/kg was even higher than that of 20 mg/kg of imipenem treating group (Fig. 8B). This indicated that targeting TarO alone could lead to a significant reduce of the MRSA’s virulence. Furthermore, when administrated **18a** at a concentration of 16 mg/kg in combination with 20 mg/kg imipenem, a further improvement in the survival rate of the infected *Galleria mellonella* larvae was observed compared with imipenem treated alone (Fig. 8B).

## Discussion

Antibiotics have empowered humans to combat bacterial infections. However, their misuse has accelerated the emergence of drug-resistant bacteria. Given that bacterial resistance evolves much more rapidly than the development of new antibacterial drugs, there is an urgent need to explore innovative antibacterial strategies. Developing adjuvants that target bacterial virulence or resistance mechanisms represents a promising strategy to combat the growing threat of drug-resistant pathogens.

Although TarO has been validated as a highly effective antibacterial target at the genetic level, research on TarO inhibitors has been progressing rather slowly. Here, utilizing the *vraX*_pro_*-lux* reporter system in hand, we designed, synthesized and efficiently screened the new Tarocin A derivatives for their TarO inhibitory activity. Overall, the novel TarO inhibitors identified in this study exhibited a strong correlation between their IC_50_ values at the cellular level and their ability to sensitize β-lactam antibiotics. Among the modified compounds, **18a** and **18b** demonstrated low lipophilicity while maintaining high activity levels. Notably, compound **18a** showed excellent anti-biofilm, anti-virulence and sensitizing β-lactams capabilities both in vitro and in vivo. These results collectively underscore that TarO is a promising drug target for combating bacterial infections.

In this study, we further confirmed that the new Tarocin A analog, compound **18a**, significantly prolongs the survival time of *Galleria mellonella* larvae infected with the MRSA HS663 strain. Given its MIC against HS663 exceeds 256 µg/mL, compound **18a** does not possess direct antibacterial activity. Thus, its capacity to improve the survival rate likely results from targeting TarO, thereby influencing the expression of virulence or immune evasion factors of HS663. In future studies, testing the *tarO* mutant would clarify whether its efficacy is achieved by targeting TarO. Moreover, compared with Tarocin A, the newly obtained compounds **10,** **18a**, and **18b** all exhibited antibiofilm activity, which was not reported in previous studies on the Tarocin A analogs.

However, this study has several limitations. First, although Tarocin A has been shown to target TarO, the target of these new inhibitors was just preliminary verified and still needs further confirmation. Secondly, **18a** significantly improved its water solubility while maintaining good activity both in vitro and in vivo, this compound did not achieve the desired result in in vitro metabolic stability test (Supplementary Table 3). Future efforts could focus on identifying the metabolites of **18a** and blocking its metabolic sites, potentially leading to the discovery of a more promising TarO inhibitor with enhanced metabolic stability and water solubility.

## Methods

### General procedure for synthesis of compounds 1–7

To a 25 mL two-necked flask equipped with a magnetic stirrer was added **31a** (0.5 mmol, l.0 equiv). The flask was then placed at -78 °C and 5 mL anhydrous THF was added under nitrogen. Then, *n*-BuLi (0.6 mmol, 1.2 equiv) was slowly added to the solution. After the reaction mixture was stirred at -78 °C for 30 min, the corresponding acyl halide (0.6 mmol, 1.2 equiv) was added. Slowly heat the reaction mixture to room temperature and continue stirring for 2 h. Then, Et_3_N (1.0 mmol, 2.0 equiv) and corresponding nucleophile (0.6 mmol, 1.2 equiv) were added. The reaction mixture was stirred continuously overnight and completion of the reaction was detected by TLC. 15 mL saturated NH_4_Cl solution and 10 mL × 3 ethyl acetate were added to the reaction solution to separate phases. The organic phase was washed with 10 mL × 3 saturated saline and dried with anhydrous Na_2_SO_4_. The crude product was concentrated in vacuum and purified by column chromatography on silica gel to afford the desired product **1–7**.

### General procedure for synthesis of compounds 8–16, 17a-d, 18a-b

To a 25 mL pear-shaped flask equipped with a magnetic stirrer were added **31a-d** (0.5 mmol, 1.0 equiv), corresponding carboxylic acid (0.6 mmol, 1.2 equiv), CMPI (0.6 mmol, 1.2 equiv), and DMAP (0.1 mmol, 0.2 equiv). Then, 5 mL DMF and Et_3_N (1.5 mmol, 3.0 equiv) was added. After completion of the addition, the reaction mixture was stirred continuously overnight at room temperature and completion of the reaction was detected by TLC. 15 mL saturated NH_4_Cl solution and 10 mL × 3 ethyl acetate were added to the reaction solution to separate phases. The organic phase was washed with 10 mL × 3 saturated saline and dried with anhydrous Na_2_SO_4_. The crude product was concentrated in vacuum and purified by column chromatography on silica gel to afford the desired product **8–16,** **17a-d,** **18a-b**.

### General procedure for synthesis of compounds 19–22

To a 25 mL pear-shaped flask equipped with a magnetic stirrer was added NaH (0.6 mmol, 1.2 equiv) (or Et_3_N for **19**), corresponding Michael donors (0.6 mmol, 1.2 equiv) and 5 mL anhydrous DMF. The reaction mixture was stirred for 15 min at room temperature. And then, **32a** (0.5 mmol, 1.0 equiv) was added. After completion of the addition, the reaction mixture was stirred for another 30 min and completion of the reaction was detected by TLC. 15 mL saturated NH_4_Cl solution and 10 mL × 3 ethyl acetate were added to the reaction solution to separate phases. The organic phase was washed with 10 mL × 3 saturated saline and dried with anhydrous Na_2_SO_4_. The crude product was concentrated in vacuum and purified by column chromatography on silica gel to afford the desired product **19–22**.

### General procedure for synthesis of compounds 23–27

To a 25 mL pear-shaped flask equipped with a magnetic stirrer was added **12** or **14** (0.1 mmol, 1.0 equiv), corresponding amine (0.15 mmol, 1.5 equiv), 50 μL acetic acid and 3 mL anhydrous methanol.The reaction mixture was stirred overnight at room temperature and completion of the reaction was detected by TLC. The reaction mixture was then concentrated in vacuum and purified by column chromatography on silica gel to afford the desired product **23–27**.

### General procedure for synthesis of compounds 31a-d

The synthesis of oxazolidine-2-one intermediates **31a-d** proceeded according to the reported route^38^.

### General procedure for synthesis of compounds 32a

To a 50 mL two-necked flask equipped with a magnetic stirrer was added **31a** (2.0 mmol, 1.0 equiv), LiCl (2.4 mmol, 1.2 equiv). Then, 10 mL anhydrous THF, acrylic anhydride (2.4 mmol, 1.2 equiv) and Et_3_N (2.4 mmol, 1.2 equiv) was added under nitrogen. After completion of the addition, the reaction mixture was stirred for 6 h and completion of the reaction was detected by TLC. The reaction mixture was then concentrated in vacuum and purified by column chromatography on silica gel to afford the desired product **32a**.

### Synthesis of partial carboxylic acids

Most carboxylic acids are obtained via the general synthetic procedure (Such as 3-(4-oxoquinolin-1(*4H*)-yl)propanoic acid):

To a 25 mL pear-shaped flask equipped with a magnetic stirrer was added quinolin-4(*1H*)-one (2.0 mmol, 1.0 equiv) and methyl acrylate (4.0 mmol, 4.0 equiv) dissolved by 5 mL AcOH. The reaction mixture was placed in an oil bath, stirred at 110 °C for 16 h. Completion of the reaction was detected by TLC and then concentrated in vacuo.

The crude product and NaOH (4.0 mmol, 4.0 equiv) were added to another 25 mL pear-shaped flask equipped with a magnetic stirrer. Dissolved them by 2 mL H_2_O and 4 mL MeOH. The reaction mixture was stirred 2 h at room temperature and completion of the reaction was detected by TLC. 10 mL H_2_O and 10 mL × 3 DCM were added to the reaction mixture. Then, 3 mL HCl (2 M) and 10 mL × 3 *n*-butanol were added to the aqueous phase. And the organic phase was concentrated in vacuo to obtain the acid. The acid was used without further purification.

Synthesis of 3-(4-oxochroman-8-yl)propanoic acid:

To a 25 mL two-necked flask equipped with a magnetic stirrer was added 8-bromochroman-4-one (2.0 mmol, 1.0 equiv), Pd_2_(dba)_3_ (0.1 mmol, 0.05 equiv), tri-tert-butylphosphine tetrafluoroborate (TTBPF) (0.2 mmol, 0.1 equiv). Then, they were dissolved by 6 mL DMF under nitrogen and Et_3_N (2.4 mmol, 1.2 equiv) was added. Put the reaction mixture in an oil bath, stirred at 120 °C for 5 h. Completion of the reaction was detected by TLC. 20 mL water and 15 mL × 3 ethyl acetate were added to the reaction mixture, the organic phase was washed with saturated saline 15 mL× 3, dried over anhydrous sodium sulfate, then concentrated in vacuo. The crude product was purified by chromatography on silica gel (petroleum ether/ethyl acetate, 2:1) to afford 3-(4-oxochroman-8-yl)propanal.

To a 25 mL pear-shaped flask equipped with a magnetic stirrer was added NaClO_2_ (4.0 mmol, 4.0 equiv) and NaH_2_PO_4_ (2.0 mmol, 2.0 equiv) dissolved by 2 mL H_2_O, 3 mL *t*-BuOH and 3 mL 2-methyibut-2-ene. Then 3-(4-oxochroman-8-yl)propanal (1.0 mmol, 1.0 equiv) was added. The reaction mixture was stirred 1 h at room temperature and completion of the reaction was detected by TLC. 5 mL HCl (2 M) was added, and filtered to give 3-(4-oxochroman-8-yl)propanoic acid as a white solid. The acid was used without further purification.

Synthesis of 3-(1-(tert-butoxycarbonyl)-4-oxo-1,2,3,4-tetrahydroquinolin-8-yl)propanoic acid:

To a 50 mL two-necked flask equipped with a magnetic stirrer was added 8-bromo-2,3-dihydroquinolin-4(*1H*)-one (3.0 mmol, 1.0 equiv), Pd(OAc)_2_ (0.15 mmol, 0.05 equiv), Tri-o-tolylphosphine (0.9 mmol, 0.3 equiv). Then, they were dissolved by 4 mL DMF and 4 mL Et_3_N under nitrogen. Put the reaction mixture in an oil bath, stirred at 120 °C for 6 h. Completion of the reaction was detected by TLC. The reaction mixture was filtered. 20 mL water and 15 mL × 3 ethyl acetate were added to the filtrate, the organic phase was washed with saturated saline 15 × 3 mL, dried over anhydrous sodium sulfate, then concentrated in vacuo. The crude product was put into a 25 mL pear-shaped flask equipped with a magnetic stirrer and dissolved by 6 mL DCM. (Boc)_2_O (4.5 mmol, 1.5 equiv), DMAP (0.6 mmool, 0.2 equiv) and Et_3_N (3.0 mmol, 1.0 equiv) was added to the solution. Then, stirred overnight at room temperature. Completion of the reaction was detected by TLC. 20 mL water and 15 mL × 3 DCM were added to the reaction mixture, the organic phase was washed with saturated saline 15 mL × 3, dried over anhydrous sodium sulfate, then concentrated in vacuo. Then, the crude product was put into another 25 mL pear-shaped flask equipped with a magnetic stirrer. HCOONH_4_ (18.0 mmol, 6.0 equiv) and 6 mL EtOH were added. The reaction mixture was heated to reflux and stirred for 2 h and then concentrated in vacuo. The crude product was purified by chromatography on silica gel (petroleum ether/ethyl acetate, 5:1) to afford tert-butyl 8-(3-methoxy-3-oxopropyl)-4-oxo-3,4-dihydroquinoline-1(*2H*)-carboxylate.

To a 25 mL pear-shaped flask equipped with a magnetic stirrer was added tert-butyl 8-(3-methoxy-3-oxopropyl)-4-oxo-3,4-dihydroquinoline-1(*2H*)-carboxylate (1.0 mmol, 1.0 equiv) and NaOH (2.0 mmol, 2.0 equiv) dissolved by 2 mL H_2_O and 4 mL MeOH. The reaction mixture was stirred 2 h at room temperature and completion of the reaction was detected by TLC. 10 mL HCl (1 M) and 10 mL × 3 ethyl acetate were added to the reaction mixture, the organic phase was washed with saturated saline 10 mL × 3, dried over anhydrous sodium sulfate, then concentrated in vacuo to obtain the acid. The acid was used without further purification.

Deprotection of Boc was carried out according to literature methods^46^.

### *vraX*_pro_*-lux* based preliminary screening

Generally, JE2::*vraX*_pro_-*lux* and its isogenic ^Tn^*tarO*::*vraX*_pro_-*lux* reporter strains were used for preliminary screening and cellular-based target verification. JE2::*vraX*_pro_-*lux* was obtained by transduction of the *vraX*_pro_-*lux* fusions from RN4220 strain to JE2, a plasmid-cured derivative of the community-associated MRSA strain USA300 LAC. ^Tn^*tarO*::*vraX*_pro_-*lux* was further obtained by transposon insertion mutation of the *tarO* gene using a mariner-based transposon in JE2 reporter strain^41^. Compounds were dissolved in DMSO to 5.12 mg/mL as the stock solution, and were further diluted with DMSO to a concentration of 100 times of the work solution. To monitor the *vraX*_pro_-*lux* expression level and the growth curve of the reporter strains, overnight cultures of the reporter strains were diluted to an OD_600_ ≈ 0.1 in fresh TSB (Tryptone Soya Broth) supplemented with or without test compounds, as indicated. Then, 200 μL aliquot of the samples were distributed to a 96-well black-wall clear-bottom plate (Costar, Corning Incorporated), and 45 μL saxoline was added to each well to prevent the evaporation of the medium. *vraX*_pro_*-lux* expression level and the growth curve were measured using a Cytation 1 Multi-Mode Microplate Reader (Biotek) at different time points as indicated.

### Quantification the activity of the new TarO inhibitors

For quantification the activity of these new TarO inhibitors, OD_600_ of the twentieth hour post sub-cultivation was extracted and calculated the relative inhibitory rate (RIR) of the compound. The calculation formula is defined as: RIR = (control group_OD600 (20h)_ - inhibitor group_OD600 (20h)_) / (control group_OD600 (20h)_ - ∆*tarO*_OD600 (20h)_). For each concentration of the inhibitors, two biological replicates were performed and the mean RIR of each concentration was submitted for IC_50_ calculation by Graphpad prism 9.5.1, using the nonlinear repression (curve fit) and choosing [inhibitor] VS. normalized response -variable slope.

### Determination of Solubility of Compounds in Water

Weigh the sample in a 10 mL volumetric flask, dissolve in methanol up to the mark to obtain a reference solution of known concentration. Inject 5 μL into the HPLC, detect peak area at 254 nm wavelength. Prepare supersaturated aqueous solution of the sample, filter and inject 5 μL into the HPLC, detect peak area at 254 nm wavelength. The solubility of the compound in water (C_A_) is calculated according to the following formula: C_S_ = C_A_ × A_B_/A_A_ (C_A_ represents the concentration of the reference solution, A_A_ represents the peak area of the reference solution, and A_B_ represents the saturated peak area of the sample).

### Antagonistic test of compound 18a against Targocil

The antagonistic effect of **18a** against Targocil was tested through a checkerboard assay. The concentrations of **18a** and Targocil varied along the rows and columns, respectively. The concentration range for **18a** was from 128 μg/mL to 0.25 μg/mL, while the concentration range for Targocil was from 64 μg/mL to 2 μg/mL. Strains without the addition of **18a** were used as controls. *S.aureus* was grown to mid log-phase and then diluted to an OD_600_ of 0.002 with fresh MHB (Mueller-Hinton broth, cation-adjusted, BD 212322) medium. 100 μL of this freshly diluted culture was added to all wells on the plate, expect for the first row which contained 190 μL diluted culture. 10 μL Targocil stock solution was added into the first row, and mixed several times by pipetting, followed by serial diluted at 1:2 to the lowest concentrations. For serially diluting **18a**, 90 μL of the last column of the mixtures were transferred into the first column, and 10 μL **18a** was added into the first column, after which the dilution was performed as the Targocil. Checkerboard MIC plates were incubated overnight at 37 °C.

### MIC determination

MIC values of the compounds were measured by broth microdilution method with MHB medium. The compounds were dissolved with DMSO to 5.12 mg/mL as the stock, diluted to 256 μg/mL with culture medium, then serial diluted at 1:2 to further reach 128 to 0.0625 μg/mL concentration. 100 μL of dilutions were distributed into 96-well microfilter plates (Thermo scientific 167008) and then 5 μL of exponential phase cultured bacteria (about 10^5^ CFUs) were added. The cultures were incubated at 37 °C for about 24 h. For negative control, same volume of DMSO was added and serially diluted as the test compounds. Vancomycin was used as a positive control. MIC values were defined as the lowest concentration that completely inhibit the growth of bacteria. The MIC of vancomycin was 1 μg/mL under these conditions.

### Checkerboard MIC assay

In the checkerboard assay, concentrations of antibiotics, oxacillin and imipenem, and TarO inhibitors were simultaneously varied across columns and the rows, respectively. In such a condition, each well had a different combination of concentrations of both compounds. The range of each compound chosen for the checkerboard assay was according to their MIC value. The concentration of oxacillin against USA300 LAC and USA400 MW2 was varied from 16 μg/mL to 0.125 μg/mL, imipenem against HS663 was varied from 32 μg/mL to 0.25 μg/mL, while the inhibitors was varied from 32 μg/mL to 0.03125 μg/mL. *S.aureus* USA300 LAC, USA400 MW2 and HS663 were grown to mid log-phase and then diluted to an OD_600_ of 0.002 with fresh MHB medium. 100 μL of this freshly diluted culture was added to all wells on the plate, expect for the first row which contained 190 μL. 10 μL antibiotic stock solutions were added into the first row, and mixed several times by pipetting, followed by serial diluted at 1:2 to the lowest concentrations. For serially diluting the inhibitors, 90 μL of the last column of the mixtures were transferred into the first column, and 10 μL inhibitors stock solution was added into the first column, after which the dilution was performed as the antibiotics. Checkerboard MIC plates were incubated overnight at 37 °C. For each clear well observed on the checkerboard MIC plate, the fractional inhibitory concentration index (FICI) was calculated as follows: FICI = FIC _(inhibitor)_ + FIC _(antibiotic)_ = MIC _inhibitor in combination_/ MIC _inhibitor alone_ + MIC _antibiotic in combination_/MIC _antibiotic alone_. FIC = Fractional Inhibitory Concentration. A FICI value of < 0.5 indicates synergy, a value between 0.5 and 2 indicates additivity or indifference, and a value > 2 indicates antagonism.

### Cytotoxicity assay

The cytotoxicity was detected by Sulforhodamine B method. The human pancreatic cancer cell line Capan-1, human colon cancer cell line HCT-116 and human lung adenocarcinoma cell line NCI-H1373 were purchased from American Type Culture Collection (ATCC; Manassas, VA) and inoculated into 96-well plates in triplicate. The cells were treated with different concentrations of compounds in humidified incubator containing 5% CO_2_ for 72 h when they reached 70%-80% confluence. Vincristine was added as a positive control. Then, SRB staining agent was added and microplate analyzer was used to read the absorbance at 450 nm wavelength. The cytotoxicity of each compound is expressed as the concentration of the compound that reduces cell viability to 50% (IC_50_). The optical density (OD) at a wavelength of 450 nm was measured using an SpectraMax 190 Microplate Reader (Molecular Devices; USA). The IC_50_ value was calculated by the Logit method using Graphpad Prism 9.1.

### Static biofilm inhibition assay

Static biofilm inhibition assays were generally performed in polystyrene 96-well plates as previously described^47^. *S.aureus* USA300 LAC and its isogenic Δ*tarO* strains were grown overnight in TSB at 37 °C with shaking at 250 rpm, and then diluted 1:100 in BHI (Brain Heart Infusion Medium) plus 1% glucose. Different concentrations of inhibitors were added to the bacteria dilutions, and 200 μL of the mixtures were distributed into the 96-well plate with 4 biological replicates. DMSO treated WT and Δ*tarO* samples were used as negative or positive control, respectively. After 24 h static culture at 37 °C, the supernatants were removed carefully and the biofilms were washed with phosphate buffer solution (PBS) for 3 times gently, followed by drying at 65 °C for 1 h. Subsequently, 150 μL methanol was used to fix the biofilm for 30 min at room temperature. After which methanol was removed and the plate was dried again. For quantification, each well was stained with 150 μL of 5% (w/v) crystal violet for approximately 30 min, then washed with sterile ultrapure water for three times and dried. Finally, the biofilm was released into solution using 200 μL of 33% glacial acetic acid and the amount of biofilm was quantified by the absorbance was read at 560 nm.

### Galleria mellonella larva infection assay

*Galleria mellonella* infection experiment was performed according to previous methods^24^. Generally, HS663 was grown overnight in TSB (Tryptone Soya Broth) at 37 °C with shaking at 250 rpm. Then cultures were diluted 1:100 into 10 ml fresh TSB, and were further grown for 3 h. The bacterial cells were harvested by centrifugation at 5700 g for 5 min, washed twice with sterilized PBS, and suspended with PBS. *Galleria mellonella* larvae (Tianjin HuiYuDe Biotechnological Co., Ltd.) about 300 mg was selected to infect. Compounds (**18a** and imipenem) were diluted with solvents containing 18% DMSO, 5% Tween 80 and 77% PBS. After infection for 1 h, the larvae were administrated with compounds or solvent only. Then the *Galleria mellonella* larvae was cultivated at 37 °C and survival rates were recorded every 12 h for 5 days to analyze the mortality data.

## Supplementary information

Supporting Information

## Data Availability

The USA300 LAC strain used in this study has its genomic information available at the National Center for Biotechnology Information (NCBI) under the accession number CP055225.1 The authors declare that the data supporting the findings of this study are available within the paper and its Supplementary Information files. Should any raw data files be needed in another format they are available from the corresponding author upon reasonable request.
