# Supplementary material for: Design, synthesis and optimization of TarO inhibitors as multifunctional antibiotics against Methicillin-resistant Staphylococcus aureus
Source: NPJ Antimicrob Resist. 2025 Apr 12;3:28. doi: 10.1038/s44259-025-00098-z (PMC11993615; doi:10.1038/s44259-025-00098-z)
Supplement: Supplementary file 1 — Supporting Information [file 44259_2025_98_MOESM1_ESM.pdf]

Supporting Information

# Design, synthesis and optimization of TarO inhibitors as multifunctional antibiotics against Methicillin-resistant *Staphylococcus aureus*

Yuanchen Zhong[a, b, +], Feifei Chen[a, b, c +], Dianyan Chen[a, c], Qian He[b], Xiaofei Zhang[b, c *], Lefu Lan[a, c, *], Chunhao Yang[b, c *]

[a] School of Pharmaceutical Science and Technology, Hangzhou Institute for Advanced Study, University of Chinese Academy of Sciences, Hangzhou 310024, China.

[b] State Key Laboratory of Drug Research, Shanghai Institute of Materia Medica, Chinese Academy of Sciences, 555 Zuchongzhi Road, Shanghai, 201203, China.

[C] University of Chinese Academy of Sciences, Beijing, 100049, China

[*] Corresponding author. E-mail addresses: xiaofeizhang@simm.ac.cn (X. Zhang), llan@ucas.ac.cn (L. Lan), chyang@simm.ac.cn (C. Yang).

[+] Contributed equally to this paper.

Content

1. Synthesis information of compounds. ....................................................................................S2
2. Induction of *vraX*$_{pro}$-*lux* expression and the growth curve of the new TarO inhibitors...........S9
3. Doses-response curve of the new TarO inhibitors.................................................................S15
4. Supplementary Table 1: The minimum sensitization concentration of some representative compounds (USA300 LAC /oxacillin)..........................................................................S17
5. Supplementary Table 2: The maximum sensitization concentration of some representative compounds (USA300 LAC /oxacillin)..........................................................................S17
6. Supplementary Table 3: Preliminary liver microsomal stability testing of TarO inhibitors..S19
7. NMR Spectra of the compounds.............................................................................................S20
8. The purity of compounds.........................................................................................................S56

## 1. Synthesis information of compounds.

*naphthalen-1-ylmethyl (4S,5R)-5-(3,5-bis(trifluoromethyl)phenyl)-4-methyl-2-oxooxazolidine-3-carboxylate (**1**)*. As white solid (31%). $^{1}$H NMR (400 MHz, $CDCl_3$) δ 8.07 (d, *J* = 8.3 Hz, 1H), 7.93-7.87 (m, 3H), 7.75 (s, 1H), 7.66 (d, *J* = 7.0 Hz, 1H), 7.59 (t, *J* = 7.5 Hz, 1H), 7.54 (t, *J* = 7.5 Hz, 1H), 7.48 (t, *J* = 7.7 Hz, 1H), 5.85-5.75 (m, 2H), 5.71 (d, *J* = 7.3 Hz, 1H), 4.67 (p, *J* = 6.7 Hz, 1H), 0.87 (d, *J* = 6.5 Hz, 3H). $^{13}$C NMR (150 MHz, $CDCl_3$) δ 150.4, 150.3, 136.4, 133.9, 132.6 (q, *J* = 33.8 Hz), 131.6, 130.2, 130.0, 129.0, 128.3, 127.0, 126.3, 126.2, 125.5, 123.4, 123.1 (dt, *J* = 7.5, 3.6 Hz), 123.0 (q, *J* = 272.7 Hz), 77.1, 67.5, 55.3, 15.5. ESI-HRMS (m/z) $[M+Na]^+$ calcd for $C_{24}H_{17}F_6NO_4$, 520.0954; found 520.0960.

*S-(naphthalen-1-ylmethyl) (4S,5R)-5-(3,5-bis(trifluoromethyl)phenyl)-4-methyl-2-oxooxazolidine-3-carbothioate (**2**)*. As white solid (26%). $^{1}$H NMR (400 MHz, $CDCl_3$) δ 8.03 (d, *J* = 8.3 Hz, 1H), 7.92 (s, 1H), 7.88 (d, *J* =8.3 Hz, 1H), 7.81 (d, *J* =8.3 Hz, 1H), 7.77 (s, 2H), 7.61-7.55 (m, 2H), 7.51 (t, *J* = 7.4 Hz, 1H), 7.41 (t, *J* = 7.6 Hz, 1H), 5.75 (d, *J* = 7.5 Hz, 1H), 4.92 (p, *J* = 6.7 Hz, 1H), 4.76-4.63 (m, 2H), 0.97 (d, *J* = 6.6 Hz, 3H). $^{13}$C NMR (100 MHz, $CDCl_3$) δ 167.9, 151.7, 136.1, 134.1, 132.6 (q, *J* = 33.9 Hz) 131.7, 131.6, 129.1, 128.9, 128.3, 126.7, 126.2 (d, *J* = 3.8 Hz), 125.5, 123.6, 123.1 (d, *J* = 3.5 Hz), 123.0 (q, *J* = 273.2 Hz), 78.1, 55.5, 32.3, 15.2. ESI-HRMS (m/z) $[M+Na]^+$ calcd for $C_{24}H_{17}F_6NO_3S$, 536.0726; found 536.0721.

*(4S,5R)-5-(3,5-bis(trifluoromethyl)phenyl)-4-methyl-N'-(naphthalen-1-yl)-2-oxooxazolidine-3-carbohydrazide (**3**)*. As brown solid (35%). $^{1}$H NMR (400 MHz, $CDCl_3$) δ 9.47 (d, *J* = 3.6 Hz, 1H), 7.97-7.87 (m, 2H), 7.85-7.80 (m, 1H), 7.74 (s, 2H), 7.52-7.44 (m, 3H), 7.38 (t, *J* = 7.8 Hz, 1H), 6.99 (d, *J* = 7.4 Hz, 1H), 6.66 (d, *J* = 3.9 Hz, 1H), 5.74 (d, *J* = 7.6 Hz, 1H), 4.83 (p, *J* = 6.7 Hz, 1H), 0.95 (d, *J* = 6.6 Hz, 3H). $^{13}$C NMR (100 MHz, $CDCl_3$) δ 154.1, 151.7, 142.3, 136.0, 134.3, 132.6 (q, *J* = 33.8 Hz), 128.8, 126.3, 126.1, 126.0, 125.8, 123.3, 123.2 (q, *J* = 3.9 Hz), 123.0 (q, *J* = 272.9 Hz), 122.0, 120.1, 78.6, 54.6, 15.5. ESI-HRMS (m/z) $[M+H]^+$ calcd for $C_{23}H_{17}F_6N_3O_3$, 498.1247; found 498.1245.

*(4S,5R)-5-(3,5-bis(trifluoromethyl)phenyl)-N'-hydroxy-4-methyl-N-(naphthalen-1-ylmethyl)-2-oxooxazolidine-3-carboximidamide (**4**)*. As white solid (52%). $^{1}$H NMR (400 MHz, $CDCl_3$) δ 8.00-7.96 (m, 1H), 7.93 (dd, *J* = 8.0, 1.7 Hz, 1H), 7.87 (d, *J* = 8.1 Hz, 1H), 7.84 (s, 1H), 7.80 (s, 2H), 7.62-7.51 (m, 3H), 7.47 (dd, *J* = 8.1, 7.0 Hz, 1H), 6.17 (t, *J* = 5.3 Hz, 1H), 5.16-5.11 (m, 1H), 4.86-4.67 (m, 2H), 4.31 (qd, *J* = 7.3, 2.3 Hz, 1H), 3.78 (s, 1H), 1.23 (d, *J* = 7.3 Hz, 1H). $^{13}$C NMR (100 MHz, $CDCl_3$) δ 158.6, 156.8, 142.6, 134.0, 132.4, 132.3 (q, *J* = 33.7 Hz), 131.4, 129.2, 129.1, 126.9, 126.8, 126.3, 126.2, 125.6, 123.1 (q, *J* = 273.1 Hz), 122.5, 75.0, 56.1, 44.2, 9.7. ESI-HRMS (m/z) $[M+H]^+$ calcd for $C_{24}H_{19}F_6N_3O_3$, 512.1403; found 512.1407.

*(4S,5R)-5-(3,5-bis(trifluoromethyl)phenyl)-4-methyl-N-(naphthalen-1-ylmethyl)-2-oxooxazolidine-3-carbothioamide (**5**)*. As brown solid (56%). $^{1}$H NMR (400 MHz, $CDCl_3$) δ 7.92 (s, 1H), 7.81-7.77(m, 2H), 7.40 (t, *J* = 7.3 Hz, 1H), 6.84 (t, *J* = 7.5 Hz, 1H), 6.78 (d, *J* = 8.7 Hz, 1H), 5.75 (d, *J* = 7.4 Hz, 1H), 4.87 (p, *J* = 6.7 Hz, 1H), 4.06 (dd, *J* = 7.9, 3.4, 2H), 3.81 (t, *J* = 6.5 Hz, 2H), 3.37-3.24 (m, 2H), 0.90 (d, *J* = 6.6 Hz, 3H). $^{13}$C NMR (100 MHz, $CDCl_3$) δ 170.8, 152.2, 143.4, 136.1, 134.3, 132.7 (q, *J* = 34.1 Hz), 126.1 (q, *J* = 3.8 Hz), 125.2, 123.6, 123.2, 123.0 (q, *J* = 272.9 Hz), 77.8, 54.5, 48.7, 48.2, 47.3, 33.5, 15.0. ESI-HRMS (m/z) $[M+H]^+$ calcd for $C_{23}H_{20}F_6N_2O_5S$, 551.1070; found

551.1070.

*(4S,5R)-5-(3,5-bis(trifluoromethyl)phenyl)-4-methyl-3-(3-(naphthalen-1-yl)azetidine-1-carbonyl)oxazolidin-2-one (**6**)*. As brown solid (30%). $^1$H NMR (400 MHz, $CDCl_3$) δ 7.93-7.88 (m, 2H), 7.83-7.78 (m, 1H), 7.77 (s, 2H), 7.74-7.69 (m, 1H), 7.54 (m, 2H), 7.50 (d, *J* = 6.6 Hz, 2H), 5.76 (d, *J* = 7.4 Hz, 1H), 4.84-4.71 (m, 3H), 4.61 (p, *J* = 6.7 Hz, 1H), 4.48 (dd, *J* = 8.9, 6.7 Hz, 2H), 0.99(m, 3H). $^{13}$C NMR (150 MHz, $CDCl_3$) δ 152.4, 151.6, 136.9, 136.2, 133.9, 132.4 (q, *J* = 33.9 Hz), 131.2 129.1, 127.8, 126.5, 126.2, 126.0, 125.4, 123.1, 122.9 (q, *J* = 272.9 Hz), 122.9, 78.0, 55.3, 31.5, 15.5. ESI-HRMS (m/z) $[M+H]^+$ calcd for $C_{26}H_{20}F_6N_2O_3$, 523.1451; found 523.1450.

*(4S,5R)-5-(3,5-bis(trifluoromethyl)phenyl)-4-methyl-3-(naphthalen-1-ylglycyl)oxazolidin-2-one (7)*. As pink solid (53%). $^1$H NMR (400 MHz, $CDCl_3$) δ 7.95 (m, 2H), 7.84-7.79 (m, 1H), 7.77 (s, 2H), 7.53-7.46 (m, 2H), 7.41-7.35 (m, 1H), 7.30 (d, *J* = 8.2 Hz, 1H), 6.64 (d, *J* = 7.4 Hz, 1H), 5.70 (d, *J* = 7.4 Hz, 1H), 4.85 (p, *J* = 6.7 Hz, 1H), 4.67 (d, *J* = 2.9 Hz, 2H), 0.93 (d, *J* = 6.6 Hz, 3H). $^{13}$C NMR (150 MHz, $CDCl_3$) δ 170.1, 152.0, 142.1, 136.0, 134.4, 132.7 (q, *J* = 33.9 Hz), 128.8, 126.7, 126.1, 125.2, 123.5, 123.2, 123.0 (q, *J* = 273.0 Hz), 120.2, 118.4, 105.1, 78.3, 54.4, 48.5, 14.9. ESI-HRMS (m/z) $[M+H]^+$ calcd for $C_{24}H_{18}F_6N_2O_3$, 497.1294; found 497.1296.

*(4S,5R)-5-(3,5-bis(trifluoromethyl)phenyl)-3-(3-cyclohexylpropanoyl)-4-methyloxazolidin-2-one (**8**)*. As white solid (40%). $^1$H NMR (400 MHz, $CDCl_3$) δ 7.92 (s, 1H), 7.80 (s, 2H), 5.75 (d, *J* =7.4 Hz, 2H), 5.75 (d, *J* =7.4 Hz, 1H), 4.88 (p, *J* = 6.7 Hz, 1H), 3.04-2.86 (m, 2H), 1.81-1.60 (m, 5H), 1.29-1.13 (m, 3H), 1.00-0.85 (m, 5H). $^{13}$C NMR (100 MHz, $CDCl_3$) δ 173.4, 152.2, 136.5, 132.6 (q, *J* = 33.8 Hz), 126.2 (d, *J* = 3.3 Hz), 123.1, 123.0 (q, *J* = 273.0 Hz), 77.5, 54.3, 37.3, 33.4, 33.2, 33.1, 31.7, 26.6, 26.3, 15.0. ESI-HRMS (m/z) $[M+H]^+$ calcd for $C_{21}H_{23}F_6NO_3$, 452.1655; found 452.1658.

*(4S,5R)-5-(3,5-bis(trifluoromethyl)phenyl)-3-(3-(1,1-dioxido-2,3-dihydro-4H-benzo[b][1,4]thiazin-4-yl)propanoyl)-4-methyloxazolidin-2-one (**9**)*. As white solid (61%). $^1$H NMR (400 MHz, $CDCl_3$) δ 10.12 (s, 1H), 7.99 (d, *J* = 8.2 Hz, 1H), 7.95-7.89 (m, 2H), 7.87 (d, *J* = 8.1 Hz, 1H), 7.62-7.51 (m, 3H), 7.46 (t, *J* = 7.6 Hz, 1H), 5.85 (d, *J* = 7.2 Hz, 1H), 5.52 (p, *J* = 6.5 Hz, 1H), 5.38-5.21 (m, 2H), 1.10 (d, *J* = 6.6 Hz, 1H). $^{13}$C NMR (150 MHz, $CDCl_3$) δ 177.4, 153.1, 136.0, 134.1, 132.7 (q, *J* = 34.0 Hz), 131.6, 131.5, 129.3, 129.1, 127.2, 127.0, 126.3, 126.1, 125.5, 123.3, 123.1 (d, *J* = 3.7 Hz), 123.0 (q, *J* = 273.4 Hz), 78.0, 58.5, 48.3, 15.0. ESI-HRMS (m/z) $[M+H]^+$ calcd for $C_{24}H_{18}F_6N_2O_2S$, 513.1066; found 513.1067.

*(4S,5R)-5-(3,5-bis(trifluoromethyl)phenyl)-3-(3-(3,4-dihydroquinolin-1(2H)-yl)propanoyl)-4-methyloxazolidin-2-one (**10**)*. As brown solid (55%). $^1$H NMR (400 MHz, $CDCl_3$) δ 7.91 (s, 1H), 7.74 (s, 2H), 7.07 (t, *J* = 7.7 Hz, 1H), 6.94 (d, *J* = 7.2 Hz,1H), 6.65 (d, *J* = 8.2 Hz, 1H), 6.60 (t, *J* = 7.3 Hz, 1H), 5.42 (d, *J* = 7.3 Hz, 1H), 4.75 (p, *J* = 6.7 Hz, 1H), 3.81 (dt, *J* = 14.6, 7.1 Hz, 1H), 3.68-3.57 (m, 1H), 3.37 (m, 3H), 3.11 (dt, *J* = 15.5, 5.9 Hz, 1H), 2.75 (t, *J* = 6.3 Hz, 2H), 1.96 (t, *J* = 6.0 Hz, 2H), 0.88 (d, *J* = 6.6 Hz, 3H). $^{13}$C NMR (100 MHz, $CDCl_3$) δ 171.9, 152.2, 144.7, 136.3, 132.6 (q, *J* = 34.0 Hz), 129.6, 127.3, 126.1 (q, *J* = 5.0 Hz), 123.1, 123.0 (q, *J* = 272.3 Hz), 122.9, 116.2, 110.7, 77.6, 54.7, 49.5, 47.1, 32.8, 28.2, 22.2, 14.9. ESI-HRMS (m/z) $[M+H]^+$ calcd for $C_{24}H_{22}F_6N_2O_3$, 501.1607; found 501.1610.

*(4S,5R)-5-(3,5-bis(trifluoromethyl)phenyl)-3-(3-(2,3-dihydro-4H-benzo[b][1,4]oxazin-4-yl)propanoyl)-4-methyloxazolidin-2-one (**11**)*. As yellow solid (65%). $^1$H NMR (400 MHz, $CDCl_3$) δ 7.92 (s, 1H), 7.77 (s, 2H), 6.89-6.81 (m, 1H), 6.78 (dd, *J* = 7.9, 1.4 Hz, 1H), 6.74 (dd, *J* = 8.1, 1.1 Hz, 1H), 6.68-6.59 (m, 1H), 5.57 (d, *J* = 7.3 Hz, 1H), 4.81 (p, *J* = 6.7 Hz, 1H), 4.25-4.21 (m, 2H), 3.79-3.59 (m, 2H), 3.48-3.40 (m, 2H), 3.38-3.15 (m, 2H), 0.89 (d, *J* = 6.6 Hz, 3H). $^{13}$C NMR (100

MHz, $CDCl_3$) δ 171.5, 152.2, 144.3, 136.2, 134.5, 132.6 (q, *J* = 33.9 Hz), 126.1, 123.1, 123.0 (q, *J* = 273.0 Hz), 118.0, 116.7, 112.1, 77.6, 64.5, 54.5, 47.4, 46.4, 32.9, 14.9. ESI-HRMS (m/z) $[M+H]^+$ calcd for $C_{23}H_{20}F_6N_2O_4$, 503.1400; found 503.1401.

*(4S,5R)-5-(3,5-bis(trifluoromethyl)phenyl)-4-methyl-3-(3-(4-oxo-3,4-dihydroquinolin-1(2H)-yl)propanoyl)oxazolidin-2-one (**12**)*. As yellow solid (68%). $^1$H NMR (400 MHz, $CDCl_3$) δ 7.91 (dd, *J* = 8.0, 1.7 Hz, 3H), 7.78 (s, 2H), 7.40 (ddd, *J* = 8.7, 7.1, 1.7 Hz, 1H), 6.79 (d, *J* = 8.5 Hz, 1H), 6.75 (t, *J* = 7.1 Hz, 1H), 5.73 (d, *J* = 7.4 Hz, 1H), 4.88 (p, *J* = 6.7 Hz, 1H), 3.79 (td, *J* = 6.9, 3.2 Hz, 2H), 3.67-3.59 (m, 2H), 3.30 (td, *J* = 6.9, 1.5 Hz, 3H), 2.73-2.66 (m, 2H), 0.91 (d, *J* = 6.6 Hz, 3H). $^{13}$C NMR (125 MHz, $CDCl_3$) δ 193.6, 171.0, 152.2, 150.8, 136.1, 135.7, 132.7 (q, *J* = 33.8 Hz), 128.7, 126.1, 123.2, 123.0 (q, *J* = 273.0 Hz), 120.2, 117.2, 112.7, 77.8, 54.5, 49.7, 46.6, 38.1, 33.3, 15.0. ESI-HRMS (m/z) $[M+H]^+$ calcd for $C_{24}H_{20}F_6N_2O_4$, 515.1400; found 515.1397.

*(4S,5R)-5-(3,5-bis(trifluoromethyl)phenyl)-4-methyl-3-(3-(2-oxo-3,4-dihydroquinolin-1(2H)-yl)propanoyl)oxazolidin-2-one (**13**)*. As white solid (58%). $^1$H NMR (400 MHz, $CDCl_3$) δ 7.92 (s, 1H), 7.80 (s, 2H), 7.24 (d, *J* = 8.0 Hz, 1H), 7.18 (d, *J* = 7.3 Hz, 1H), 7.02 (t, *J* = 7.9 Hz, 2H), 5.77 (d, *J* = 7.4 Hz, 1H), 4.83 (p, *J* = 6.8 Hz, 1H), 4.43-4.25 (m, 1H), 3.37 (dt, *J* = 16.4, 7.9 Hz, 1H), 3.22 (dt, *J* = 15.7, 7.4 Hz, 1H), 2.91 (dd, *J* = 8.5, 6.3 Hz, 2H), 2.65 (dd, *J* = 8.7, 6.1 Hz, 2H), 0.89 (d, *J* = 6.6 Hz, 3H). $^{13}$C NMR (100 MHz, $CDCl_3$) δ 170.6, 152.4, 139.4, 136.4, 132.6 (q, *J* = 33.8 Hz), 128.4, 127.7, 126.8, 126.2, 123.2, 123.1, 123.0 (q, *J* = 273.0 Hz), 77.8, 54.5, 38.1, 34.0, 31.9, 25.6, 14.9. ESI-HRMS (m/z) $[M+H]^+$ calcd for $C_{24}H_{20}F_6N_2O_3$, 515.1400; found 515.1401.

*(4S,5R)-5-(3,5-bis(trifluoromethyl)phenyl)-4-methyl-3-(3-(4-oxochroman-8-yl)propanoyl)oxazolidin-2-one (**14**)*. As white solid (65%). $^1$H NMR (400 MHz, $CDCl_3$) δ 7.92 (s, 1H), 7.83-7.77 (m, 3H), 7.41 (dd, *J* = 7.4, 1.8 Hz, 1H), 6.95 (t, *J* = 7.6 Hz, 1H), 5.74 (d, *J* = 7.3 Hz, 1H), 4.88 (p, *J* = 6.8 Hz, 1H), 4.58 (t, *J* = 6.4 Hz, 2H), 3.34-3.20 (m, 2H), 3.02 (m, 2H), 2.82 (t, *J* = 6.4 Hz, 2H), 0.90 (d, *J* = 6.6 Hz, 3H). $^{13}$C NMR (150 MHz, $CDCl_3$) δ 192.2, 172.1, 160.1, 152.2, 136.6, 136.3, 132.6 (q, *J* = 34.1 Hz), 129.3, 126.1, 125.8, 123.1, 123.0 (q, *J* = 273.1 Hz) 121.5, 121.1, 77.6, 67.2, 54.4, 37.8, 35.5, 24.8, 15.0. ESI-HRMS (m/z) $[M+H]^+$ calcd for $C_{24}H_{19}F_6NO_5$, 516.1240; found 516.1243.

*(4S,5R)-5-(3,5-bis(trifluoromethyl)phenyl)-4-methyl-3-(3-(4-oxo-1,2,3,4-tetrahydroquinolin-8-yl)propanoyl)oxazolidin-2-one (**15**)*. As yellow solid (44%). $^1$H NMR (600 MHz, $CDCl_3$) δ 7.93 (s, 1H), 7.80 (s, 2H), 7.79 (dd, *J* = 7.9, 1.3 Hz, 1H), 7.23 (dd, *J* = 7.2, 1.3 Hz, 1H), 6.68 (t, *J* = 7.6 Hz, 1H), 5.81 (d, *J* = 7.4 Hz, 1H), 5.32 (s, 1H), 4.92 (p, *J* = 6.7 Hz, 1H), 3.62 (t, *J* = 6.6 Hz, 2H), 3.26 (ddd, *J* = 15.5, 9.0, 6.4 Hz, 1H), 3.12 (ddd, J = 15.8, 8.7, 7.3 Hz, 1H), 2.91-2.84 (m, 2H), 2.71-2.67 (m, 2H), 0.93 (d, *J* = 6.6 Hz, 3H). $^{13}$C NMR (150 MHz, $CDCl_3$) δ 194.2, 171.9, 152.5, 150.3, 136.1, 135.6, 132.7 (q, *J* = 33.9 Hz), 126.7, 126.1, 125.0, 123.2, 123.0 (q, *J* = 273.0 Hz), 119.7, 117.3, 77.9, 54.5, 42.0, 37.8, 35.7, 26.3, 15.0. ESI-HRMS (m/z) $[M+H]^+$ calcd for $C_{24}H_{20}F_6N_2O_4$, 515.1400; found 515.1401.

*(4S,5R)-5-(3,5-bis(trifluoromethyl)phenyl)-4-methyl-3-(3-(7-oxothieno[3,2-b]pyridin-4(7H)-yl)propanoyl)oxazolidin-2-one (**16**)*. As yellow solid (42%). $^1$H NMR (400 MHz, $CDCl_3$) δ 8.15 (s, 2H), 8.09 (s, 1H), 8.04 (d, *J* = 5.6 Hz, 1H), 8.00 (d, *J* = 7.4 Hz, 1H), 7.60 (d, *J* = 5.6 Hz, 1H), 6.37 (d, *J* = 7.4 Hz, 1H), 6.12 (d, *J* = 7.7 Hz, 1H), 5.09 (q, *J* = 6.5 Hz, 1H), 4.71 (t, *J* = 6.5 Hz, 2H), 3.57 (td, *J* = 6.5, 2.6 Hz, 2H), 0.90 (d, *J* = 6.7 Hz, 3H). $^{13}$C NMR (125 MHz, $CDCl_3$) δ 170.7, 153.2, 145.7, 143.1, 138.7, 133.7, 132.3 (q, *J* = 33.5 Hz), 130.1, 128.0, 124.2 (q, *J* = 272.2 Hz), 123.3, 118.3, 110.9, 78.5, 54.8, 50.0, 36.4, 15.0. ESI-HRMS (m/z) $[M+H]^+$ calcd for $C_{22}H_{16}F_6N_2O_4S$, 519.0808; found 519.0808.

*(4S,5R)-5-(3,5-bis(trifluoromethyl)phenyl)-4-methyl-3-(3-(4-oxoquinolin-1(4H)-yl)propanoyl)oxazolidin-2-one (**17a**)*. As white solid (64%). $^1$H NMR (400 MHz, $CDCl_3$) δ 8.47 (dd, *J* = 8.1, 1.5 Hz, 1H), 7.92 (s, 1H), 7.79-7.75 (m, 3H), 7.69 (ddd, *J* = 8.6, 7.1, 1.6 Hz, 1H), 7.45 (d, *J* = 8.6 Hz, 1H), 7.40 (t, *J* = 7.5 Hz, 1H), 6.26 (d, *J* = 7.8 Hz, 1H), 5.77 (d, *J* = 7.4 Hz, 1H), 4.88 (p, *J* = 6.7 Hz, 1H), 4.54 (t, *J* = 6.2 Hz, 2H), 3.53 (t, *J* = 6.2 Hz, 2H), 0.91 (d, *J* = 6.6 Hz, 3H). $^{13}$C NMR (100 MHz, $CDCl_3$) δ 178.3, 169.7, 152.1, 144.2, 139.5, 135.9, 132.7 (q, *J* = 33.7 Hz), 132.6, 127.6, 127.5, 126.1, 123.9, 123.2, 122.9 (q, *J* = 272.9 Hz), 115.0, 110.2, 77.9, 54.5, 47.4, 35.1, 14.9. ESI-HRMS (m/z) $[M+H]^+$ calcd for $C_{24}H_{18}F_6N_2O_4$, 513.1244; found 513.1243.

*(4S,5S)-5-(6-methoxy-4-(trifluoromethyl)pyridin-2-yl)-4-methyl-3-(3-(4-oxoquinolin-1(4H)-yl)propanoyl)oxazolidin-2-one (**17b**)*. As white solid (44%). $^1$H NMR (400 MHz, $CDCl_3$) δ 8.56 (dd, *J* = 8.2, 1.6 Hz, 1H), 8.10 (d, *J* = 7.4 Hz, 1H), 7.76 (ddd, *J* = 8.7, 7.0, 1.7 Hz, 1H), 7.58 (d, *J* = 8.7 Hz, 1H), 7.45 (ddd, *J* = 8.0, 7.1, 0.9 Hz, 1H), 7.23 (d, *J* = 1.2 Hz, 1H), 7.03 (br, 1H), 6.94 (dd, *J* = 1.5, 0.8 Hz, 1H), 5.62 (d, *J* = 7.4 Hz, 1H), 4.96 (p, *J* = 6.6 Hz, 1H), 4.72-4.66 (m, 2H), 3.92 (s, 3H), 3.64-3.45 (m, 2H), 0.95 (d, *J* = 6.5 Hz, 3H). $^{13}$C NMR (150 MHz, $CDCl_3$) δ 178.8, 169.5, 164.5, 153.2, 152.6, 146.1, 142.0 (d, *J* = 34.9 Hz), 139.2, 133.5, 127.8, 126.6, 124.9, 122.5 (q, *J* = 273.5 Hz), 115.4, 110.1, 109.1, 108.1, 78.5, 54.4, 48.3, 35.3, 29.4, 14.5. ESI-HRMS (m/z) $[M+H]^+$ calcd for $C_{23}H_{20}F_3N_3O_5$, 476.1428; found 476.1430.

*(4S,5R)-5-(3-fluoro-5-(trifluoromethyl)phenyl)-4-methyl-3-(3-(4-oxoquinolin-1(4H)-yl)propanoyl)oxazolidin-2-one (**17c**)*. As white solid (40%). $^1$H NMR (400 MHz, $CDCl_3$) δ 8.44 (dd, *J* = 8.1, 1.6 Hz, 1H), 7.94 (d, *J* = 7.6 Hz, 1H), 7.68 (ddd, *J* = 8.6, 7.0, 1.6 Hz, 1H), 7.49 (d, *J* = 8.7 Hz, 1H), 7.41-7.30 (m, 3H), 7.24 (t, *J* = 1.9 Hz, 1H), 6.56 (br, 1H), 5.77 (d, *J* = 7.3 Hz, 1H), 4.84 (p, *J* = 6.7 Hz, 1H), 4.60 (t, *J* = 6.4, 2H), 3.52 (dt, *J* = 8.5, 6.5 Hz, 2H), 0.89 (d, *J* = 6.6 Hz, 3H). $^{13}$C NMR (125 MHz, $CDCl_3$) δ 178.4, 169.7, 162.7 (d, *J* = 251.2 Hz), 152.4, 145.2, 139.3, 137.2 (d, *J* = 7.5 Hz), 133.3 (d, *J* = 8.0 Hz), 133.0, 127.5, 126.9, 124.0, 123.0 (q, *J* = 273.0 Hz), 118.5, 116.7 (d, *J* = 23.4 Hz), 115.3, 113.6 (d, *J* = 24.0 Hz), 110.0, 78.0, 54.6, 47.9, 35.1, 14.7. ESI-HRMS (m/z) $[M+H]^+$ calcd for $C_{23}H_{18}F_4N_2O_4$, 463.1275; found 463.1276.

*(4S,5R)-4-methyl-3-(3-(4-oxoquinolin-1(4H)-yl)propanoyl)-5-(3-(trifluoromethyl)phenyl)oxazolidin-2-one (**17d**)*. As white solid (45%). $^1$H NMR (400 MHz, $CDCl_3$) δ 8.48 (dd, *J* = 8.2, 1.6 Hz, 1H), 7.96 (d, *J* = 7.6 Hz, 1H), 7.71 (ddd, *J* = 8.6, 7.0, 1.6 Hz, 1H), 7.65 (d, *J* = 7.7 Hz, 1H), 7.60-7.56 (m, 2H), 7.55-7.47 (m, 2H), 7.40 (dd, *J* = 8.1, 7.1 Hz, 1H), 6.59 (br, 1H), 5.79 (d, *J* = 7.3 Hz, 1H), 4.84 (p, *J* = 6.7 Hz, 1H), 4.63 (t, *J* = 6.4 Hz, 2H), 3.55 (q, *J* = 6.1 Hz, 2H), 0.99 (d, *J* = 6.6 Hz, 3H). $^{13}$C NMR (125 MHz, $CDCl_3$) δ 178.4, 169.7, 152.7, 145.2, 139.3, 134.3, 133.0, 131.4 (q, *J* = 32.8 Hz), 129.6, 129.2, 127.5, 126.9, 125.9, 124.4, 123.8 (q, *J* = 272.4 Hz), 122.6, 115.3, 110.0, 78.7, 54.8, 48.0, 35.1, 14.8. ESI-HRMS (m/z) $[M+H]^+$ calcd for $C_{23}H_{19}F_3N_2O_4$, 445.1370; found 445.1373.

*(4S,5R)-5-(3,5-bis(trifluoromethyl)phenyl)-4-methyl-3-(3-(4-oxo-1,8-naphthyridin-1(4H)-yl)propanoyl)oxazolidin-2-one (**18a**)*. As white solid (56%). $^1$H NMR (400 MHz, $CDCl_3$) δ 8.74 (dd, *J* = 4.5, 1.9 Hz, 1H), 8.71 (dd, *J* = 8.0, 2.0 Hz, 1H), 8.08 (d, *J* = 7.8 Hz, 1H), 7.89 (s, 1H), 7.78 (s, 2H), 7.36 (dd, *J* = 8.0, 4.5 Hz, 1H), 6.56 (d, *J* = 7.5 Hz, 1H), 5.82 (d, *J* = 7.4 Hz, 1H), 4.96-4.78 (m, 2H), 4.68 (p, *J* = 6.5 Hz, 1H), 3.72-3.46 (m, 2H), 0.88 (d, *J* = 6.6 Hz, 3H). $^{13}$C NMR (125 MHz, $CDCl_3$) δ 179.2, 170.6, 152.8, 152.2, 149.5, 145.5, 136.4, 136.2, 132.6 (q, *J* = 33.8 Hz), 126.2, 123.1, 123.0 (q, *J* = 273.1 Hz), 121.8, 120.4, 77.9, 54.4, 46.7, 35.8, 15.0. ESI-HRMS (m/z) $[M+H]^+$ calcd for $C_{23}H_{17}F_6N_3O_4$, 514.1196; found 514.1197.

*(4S,5S)-5-(6-methoxy-4-(trifluoromethyl)pyridin-2-yl)-4-methyl-3-(3-(4-oxo-1,8-naphthyridin-1(4H)-yl)propanoyl)oxazolidin-2-one (**18b**)*. As white solid (46%). $^1$H NMR (400 MHz, $CDCl_3$) δ 8.70 (dd, *J* = 4.5, 1.9 Hz, 1H), 8.65 (dd, *J* = 7.9, 1.9 Hz, 1H), 7.91 (d, *J* = 7.9 Hz, 1H), 7.32 (dd, *J* = 7.9, 4.5 Hz, 1H), 7.23 (s, 1H), 6.26 (d, *J* = 7.9 Hz, 1H), 5.55 (d, *J* = 7.5 Hz, 1H), 4.92 (p, *J* = 6.6 Hz, 1H), 4.77-4.59 (m, 2H), 3.90 (s, 3H), 3.64-3.48 (m, 2H), 0.93 (d, *J* = 6.5 Hz, 3H). $^{13}$C NMR (100 MHz, $CDCl_3$) δ 178.9, 170.7, 164.4, 153.2, 152.4, 152.3, 149.7, 144.5, 141.9 (q, *J* = 34.2 Hz), 136.2, 122.4 (q, *J* = 273.3 Hz), 122.0, 119.9, 110.8, 109.0, 108.0, 78.2, 54.3, 54.1, 35.8, 14.4. ESI-HRMS (m/z) $[M+H]^+$ calcd for $C_{22}H_{19}F_3N_4O_5$, 477.1380; found 477.1383.

*(4S,5R)-5-(3,5-bis(trifluoromethyl)phenyl)-3-(3-(1,1-dioxidothiomorpholino)propanoyl)-4-methyloxazolidin-2-one (**19**)*. As white solid (32%). $^1$H NMR (400 MHz, $CDCl_3$) δ 7.92 (s, 1H), 7.80 (s, 2H), 5.79 (d, *J* = 7.4 Hz, 1H), 4.90 (p, *J* = 6.7 Hz, 1H), 3.24-3.01 (m, 10H), 2.97-2.91 (m, 2H), 0.90 (d, *J* = 6.6 Hz, 2H). $^{13}$C NMR (151 MHz, $CDCl_3$) δ 171.0, 152.3, 136.2, 132.6 (q, *J* = 33.9 Hz), 126.1, 123.1, 123.0 (q, *J* = 272.9 Hz), 77.7, 54.4, 51.8, 51.4, 50.9, 33.8, 14.9. ESI-HRMS (m/z) $[M+H]^+$ calcd for $C_{19}H_{20}F_6N_2O_5S$, 503.1070; found 503.1073.

*(4S,5R)-5-(3,5-bis(trifluoromethyl)phenyl)-3-(3-(6-fluoro-4-oxoquinolin-1(4H)-yl)propanoyl)-4-methyloxazolidin-2-one (**20**)*. As white solid (24%). $^1$H NMR (400 MHz, $CDCl_3$) δ 8.11 (dd, *J* = 8.9, 2.3, 1H), 7.93 (s, 1H), 7.77 (s, 2H), 7.50-7.40 (m, 2H), 6.23 (d, *J* = 7.7 Hz, 1H), 5.78 (d, *J* = 7.4 Hz, 1H), 4.89 (p, *J* =6.7 Hz, 1H), 4.54 (t, *J* = 6.2 Hz, 2H), 3.51 (q, *J* = 6.0 Hz, 2H), 0.91 (d, *J* = 6.6 Hz, 2H). $^{13}$C NMR (125 MHz, $CDCl_3$) δ 177.5, 169.6, 159.3 (d, *J* = 246.4 Hz), 152.1, 144.2, 136.0, 135.8, 132.7 (q, *J* = 33.9 Hz), 129.1 (d, *J* = 6.7 Hz), 126.1, 123.3, 122.9 (q, *J* = 273.0 Hz), 121.1 (d, *J* = 25.1 Hz), 117.2 (d, *J* = 7.6 Hz), 112.3 (d, *J* = 22.4 Hz), 109.6, 78.0, 54.5, 47.7, 35.1, 15.0. ESI-HRMS (m/z) $[M+H]^+$ calcd for $C_{24}H_{17}F_7N_2O_4$, 531.1149; found 531.1150.

*(4S,5R)-5-(3,5-bis(trifluoromethyl)phenyl)-3-(3-(7-fluoro-4-oxoquinolin-1(4H)-yl)propanoyl)-4-methyloxazolidin-2-one (**21**)*. As white solid (20%). $^1$H NMR (400 MHz, $CDCl_3$) δ 8.47 (dd, *J* = 9.2, 6.6 Hz, 1H), 7.93 (s, 1H), 7.77 (s, 2H), 7.74 (d, *J* = 7.8 Hz, 1H), 7.15-7.08 (m, 2H), 6.24 (d, *J* = 7.8 Hz, 1H), 5.79 (d, *J* = 7.4 Hz, 1H), 4.89 (p, *J* = 6.7 Hz, 1H), 4.47 (td, *J* = 6.3, 3.0 Hz, 2H), 3.50 (td, *J* = 7.0, 6.5 Hz, 1H), 0.92 (d, *J* = 6.6 Hz, 3H). $^{13}$C NMR (150 MHz, $CDCl_3$) δ 177.5, 169.4, 165.2 (d, *J* = 252.2 Hz), 152.0, 144.3, 140.9 (d, *J* = 11.2 Hz), 135.7, 132.6 (q, *J* = 33.9 Hz), 130.5 (d, *J* = 10.7 Hz), 126.0, 123.7, 123.1, 123.0 (q, *J* = 273.1 Hz), 112.6 (d, *J* = 11.2 Hz), 110.6, 101.1 (d, *J* = 26.5 Hz), 77.9, 54.4, 47.4, 34.8, 14.8. ESI-HRMS (m/z) $[M+H]^+$ calcd for $C_{24}H_{17}F_7N_2O_4$, 531.1149; found 531.1149.

*(4S,5R)-5-(3,5-bis(trifluoromethyl)phenyl)-3-(3-(6-methoxy-4-oxoquinolin-1(4H)-yl)propanoyl)-4-methyloxazolidin-2-one (**22**)*. As white solid (22%). $^1$H NMR (400 MHz, $CDCl_3$) δ 7.92 (s, 1H), 7.87 (d, *J* = 2.8 Hz, 1H), 7.76 (s, 2H), 7.73 (d, *J* =7.7 Hz, 1H), 7.41 (d, *J* = 9.2 Hz, 1H), 7.32 (dd, *J* = 9.2, 2.8 Hz, 1H), 6.25 (d, *J* = 7.6 Hz, 1H), 5.76 (d, *J* = 7.3, 1H), 4.87 (p, *J* = 6.7 Hz, 1H), 4.54 (t, *J* = 6.1 Hz, 1H), 3.93 (s, 3H), 3.51 (t, *J* = 6.0 Hz), 0.91 (d, *J* = 6.6 Hz, 3H). $^{13}$C NMR (125 MHz, $CDCl_3$) δ 177.8, 169.7, 156.5, 152.1, 143.2, 135.9, 134.0, 132.7 (q, *J* = 33.8 Hz), 128.7, 126.1, 123.3, 123.2, 123.0 (q, *J* = 273.3 Hz), 116.7, 109.2, 106.6, 77.9, 56.0, 54.5, 47.6, 35.2, 15.0. ESI-HRMS (m/z) $[M+H]^+$ calcd for $C_{25}H_{20}F_6N_2O_5$, 543.1349; found 543.1352.

*(4S,5R)-5-(3,5-bis(trifluoromethyl)phenyl)-3-(3-((E)-4-(hydroxyimino)chroman-8-yl)propanoyl)-4-methyloxazolidin-2-one (**23**)*. As white solid (85%). $^1$H NMR (400 MHz, $CDCl_3$) δ 7.92 (s, 1H), 7.78 (s, 2H), 7.72 (d, *J* = 7.8 Hz, 1H), 7.19 (d, *J* = 7.2 Hz, 1H), 6.87 (t, *J* = 7.6 Hz, 1H), 5.72 (d, *J* = 7.4 Hz, 1H), 4.87 (p, *J* = 6.7 Hz, 1H), 4.28 (t, *J* = 6.2 Hz, 2H), 3.25 (t, *J* = 6.2 Hz, 2H), 3.00 (t, *J* =

6.2 Hz, 4H), 0.9 (d, $J$ = 6.6 Hz, 3H). $^{13}$C NMR (100 MHz, $CDCl_3$) δ 172.4, 155.0, 152.2, 150.3, 136.4, 132.6 (q, $J$ = 33.8 Hz), 131.9, 129.1, 126.1 (q, $J$ = 4.9 Hz), 123.1, 123.0 (q, $J$ = 273.1 Hz), 122.6, 121.1, 118.4, 77.6, 65.1, 54.4, 35.6, 25.1, 23.6, 15.0. ESI-HRMS (m/z) $[M+H]^+$ calcd for $C_{24}H_{20}F_6N_2O_5$, 531.1349; found 531.1349.

*(4S,5R)-5-(3,5-bis(trifluoromethyl)phenyl)-3-(3-((E)-4-((2-hydroxyethoxy)imino)chroman-8-yl)propanoyl)-4-methyloxazolidin-2-one (**24**)*. As white solid (63%). $^1$H NMR (400 MHz, $CDCl_3$) δ 7.92 (s, 1H), 7.83 (dd, $J$ = 7.9, 1.7 Hz, 1H), 7.77 (s, 2H), 7.25-7.21 (m, 1H), 6.79-6.67 (m, 2H), 5.68 (d, $J$ = 7.4 Hz, 1H), 4.85 (p, $J$ = 6.7 Hz, 1H), 4.31-4.23 (m, 2H), 3.96-3.90 (m, 2H), 3.80-3.61 (m, 2H), 3.34 (t, $J$ = 6.6 Hz, 1H), 3.29-3.21 (m, 2H), 2.89 (td, $J$ = 6.4, 3.9 Hz, 2H), 2.68 (br, 1H), 0.9 (d, $J$ = 6.6 Hz, 3H). $^{13}$C NMR (100 MHz, $CDCl_3$) δ 171.3, 152.4, 152.2, 146.5, 136.2, 132.6 (q, $J$ = 33.5 Hz), 131.2, 126.1, 125.0, 123.2, 123.0 (q, $J$ = 272.8 Hz), 117.8, 117.5, 112.4, 77.7, 74.9, 63.3, 54.5, 47.8, 46.6, 32.9, 24.2, 15.0. ESI-HRMS (m/z) $[M+H]^+$ calcd for $C_{26}H_{24}F_6N_2O_6$, 575.1611; found 575.1612.

*(4S,5R)-5-(3,5-bis(trifluoromethyl)phenyl)-3-(3-((E)-4-((2-(dimethylamino)ethoxy)imino)chroman-8-yl)propanoyl)-4-methyloxazolidin-2-one (**25**)*. As white solid (52%). $^1$H NMR (400 MHz, $CDCl_3$) δ 7.88 (s, 1H), 7.79 (s, 2H), 7.71 (d, $J$ = 8.0 Hz, 1H), 7.16 (d, $J$ = 7.3 Hz, 1H), 6.83 (t, $J$ = 7.6 Hz, 1H), 5.76 (d, $J$ = 7.3 Hz, 1H), 4.88 (p, $J$ = 6.6 Hz, 1H), 4.63-4.54 (m, 2H), 4.22 (t, $J$ = 6.3 Hz, 2H), 3.47-3.38 (m, 2H), 3.19 (t, $J$ = 7.4 Hz, 2H), 2.96-2.91 (m, 4H), 2.87 (s, 6H), 0.87 (d, $J$ = 6.5 Hz, 3H). $^{13}$C NMR (100 MHz, $CDCl_3$) δ 172.2, 155.2, 151.0, 136.4, 132.4 (q, $J$ = 33.8 Hz), 132.2, 129.2, 126.1, 123.0 (q, $J$ = 273.0 Hz), 122.7, 121.0, 117.8, 77.6, 68.5, 65.0, 56.4, 54.3, 43.9, 35.6, 25.0, 24.6, 14.9. ESI-HRMS (m/z) $[M+H]^+$ calcd for $C_{28}H_{29}F_6N_3O_5$, 602.2084; found 602.2087.

*(4S,5R)-5-(3,5-bis(trifluoromethyl)phenyl)-3-(3-((E)-4-(hydroxyimino)-3,4-dihydroquinolin-1(2H)-yl)propanoyl)-4-methyloxazolidin-2-one (**26**)*. As yellow solid (80%). $^1$H NMR (400 MHz, $CDCl_3$) δ 8.24 (br, 1H), 7.92 (s, 1H), 7.82 (dd, $J$ = 7.9, 1.5 Hz, 1H), 7.77 (s, 2H), 6.79-6.70 (m, 1H), 5.68 (d, $J$ = 7.3 Hz, 1H), 4.86 (p, $J$ = 6.7 Hz, 1H), 3.81-3.67 (m, 2H), 3.36 (t, $J$ = 6.6 Hz, 2H), 3.32-3.22 (m, 2H), 2.99-2.88 (m, 2H), 0.91 (d, $J$ = 6.6 Hz, 3H). $^{13}$C NMR (151 MHz, $CDCl_3$) δ 171.3, 152.7, 152.2, 146.4, 136.2, 132.6 (q, $J$ = 34.0 Hz), 131.1, 126.1, 124.9, 123.1, 123.0 (q, $J$ = 272.9 Hz), 117.8, 117.7, 112.4, 77.7, 54.5, 47.9, 46.7, 32.9, 23.5, 15.0. ESI-HRMS (m/z) $[M+H]^+$ calcd for $C_{24}H_{21}F_6N_3O_4$, 530.1509; found 530.1510.

*(4S,5R)-5-(3,5-bis(trifluoromethyl)phenyl)-3-(3-((E)-4-((2-hydroxyethoxy)imino)-3,4-dihydroquinolin-1(2H)-yl)propanoyl)-4-methyloxazolidin-2-one (**27**)*. As white solid (55%). $^1$H NMR (400 MHz, $CDCl_3$) δ 7.92 (s, 1H), 7.78 (s, 2H), 7.73 (dd, $J$ = 8.0, 1.7 Hz, 1H), 7.19 (dd, $J$ = 7.5, 1.7 Hz, 1H), 6.86 (t, $J$ = 7.7 Hz, 1H), 5.72 (d, $J$ = 7.3 Hz, 1H), 4.86 (p, $J$ = 6.7 Hz, 1H), 4.34-4.20 (m, 4H), 3.94 (br, 2H), 3.29-3.18 (m, 2H), 3.03-2.91 (m, 4H), 2.53 (br, 1H), 0.89 (d, $J$ =6.6 Hz, 3H). $^{13}$C NMR (100 MHz, $CDCl_3$) δ 172.3, 155.0, 152.2, 149.8, 136.4, 132.6 (q, $J$ = 33.8 Hz), 132.0, 129.1, 126.1, 123.1, 123.0 (q, $J$ = 272.3 Hz), 121.1, 118.2, 77.6, 75.2, 65.0, 63.1, 54.4, 35.6, 25.1, 24.3, 15.0. ESI-HRMS (m/z) $[M+H]^+$ calcd for $C_{26}H_{25}F_6N_3O_5$, 574.1771; found 574.1772.

The synthesis of oxazolidine-2-one intermediates **31a-d** proceeded according to the reported route.[1]

*(4S,5R)-5-(3,5-bis(trifluoromethyl)phenyl)-4-methyloxazolidin-2-one (**31a**)*. As white solid (45% yield over 2 steps). $^1$H NMR (400 MHz, $CDCl_3$) δ 7.90 (s, 1H), 7.78 (s, 2H), 5.98 (s, 1H), 5.83 (d, $J$ = 8.0 Hz, 1H), 4.32 (p, $J$ = 6.6 Hz, 1H), 0.83 (d, $J$ = 6.6 Hz, 3H). $^{13}$C NMR (150 MHz, $CDCl_3$) δ 158.8, 137.9, 132.4 (q, $J$ = 33.7 Hz), 126.3, 123.1 (q, $J$ = 272.8 Hz), 122.8, 79.6, 52.2, 17.9. ESI-

LRMS (m/z) $[M+H]^+$ 314.2.

*(4S,5S)-5-(6-methoxy-4-(trifluoromethyl)pyridin-2-yl)-4-methyloxazolidin-2-one* ***(31b)***. As white solid (49% yield over 2 steps). $^1H$ NMR (400 MHz, $CDCl_3$) δ 7.24 (s, 1H), 6.91 (s, 1H), 6.88 (s, 1H), 5.63 (d, *J* = 8.2 Hz, 1H), 4.43-4.29 (m, 1H), 3.91 (s, 3H), 0.86 (d, *J* = 6.5 Hz, 3H). $^{13}C$ NMR (150 MHz, $CDCl_3$) δ 164.1, 159.3, 155.2, 141.7 (q, *J* = 34.0 Hz), 122.6 (q, *J* = 273.4 Hz), 109.0, 107.3, 80.1, 54.1, 51.9, 17.2. ESI-LRMS (m/z) $[M+H]^+$ 277.2.

*(4S,5R)-5-(3-fluoro-5-(trifluoromethyl)phenyl)-4-methyloxazolidin-2-one* ***(31c)***. As white solid (34% yield over 2 steps). $^1H$ NMR (400 MHz, $CDCl_3$) δ 7.35 (s, 1H), 7.31 (s, 1H), 6.52 (s, 1H), 5.70 (d, *J* = 7.6 Hz, 1H), 4.34-4.23 (m, 1H), 0.85 (d, *J* = 6.6 Hz, 3H). $^{13}C$ NMR (150 MHz, DMSO) δ 162.0 (d, *J* = 247.4 Hz), 157.6, 140.8 (d, *J* = 7.7 Hz), 131.8 (d, *J* = 8.8 Hz), 123.2 (d, *J* = 272.7 Hz), 118.8, 117.3 (d, *J* = 22.7 Hz), 112.5 (d, *J* = 25.1 Hz), 78.0, 50.9, 17.0. ESI-LRMS (m/z) $[M+H]^+$ 264.2.

*(4S,5R)-4-methyl-5-(3-(trifluoromethyl)phenyl)oxazolidin-2-one* ***(31d)***. As white solid (37% yield over 2 steps). $^1H$ NMR (400 MHz, $CDCl_3$) δ 7.60 (d, *J* = 7.4 Hz, 1H), 7.56-7.46 (m, 3H), 6.84 (s, 1H), 5.75 (d, *J* = 8.1 Hz, 1H), 4.31-4.21 (m, 1H), 0.80 (d, *J* = 6.6 Hz, 3H). $^{13}C$ NMR (150 MHz, $CDCl_3$) δ 159.6, 136.2, 131.1 (q, *J* = 32.6 Hz), 129.4, 129.2, 125.5, 123.9 (q, *J* = 272.4 Hz), 122.9, 80.3, 52.3, 17.6. ESI-LRMS (m/z) $[M+H]^+$ 246.2.

*(4S,5R)-3-acryloyl-5-(3,5-bis(trifluoromethyl)phenyl)-4-methyloxazolidin-2-one* ***(32a)***. As white solid (54%). $^1H$ NMR (600 MHz, $CDCl_3$) δ 7.92 (s, 1H), 7.81 (s, 2H), 7.49 (dd, *J* = 17.0, 10.5 Hz, 1H), 6.60 (d, *J* = 16.9 Hz, 1H), 5.96 (d, *J* = 10.9 Hz, 1H), 5.80 (d, *J* = 7.3 Hz, 1H), 4.96 (p, *J* = 6.8 Hz, 1H), 0.96 (d, *J* = 6.6 Hz, 3H). $^{13}C$ NMR (150 MHz, $CDCl_3$) δ 164.5, 152.1, 136.4, 132.7 (q, *J* = 33.9 Hz), 132.6, 126.2, 127.2, 123.1, 123.0 (q, *J* = 273.0 Hz), 77.7, 54.6, 15.0. ESI-LRMS (m/z) $[M+H]^+$ 367.2.

*3-(4-oxochroman-8-yl)propanal*. As yellow liquid (54%). $^1H$ NMR (400 MHz, $CDCl_3$) δ 9.80 (t, *J* = 1.3 Hz, 1H), 7.76 (dd, *J* = 7.9, 1.7 Hz, 1H), 7.33 (dd, *J* = 7.3, 1.6 Hz, 1H), 6.92 (t, *J* = 7.6 Hz, 1H), 4.57-4.49 (m, 1H), 2.93 (t, *J* = 7.4 Hz, 1H), 2.82-2.77 (m, 1H), 2.76-2.71 (m, 2H). ESI-LRMS (m/z) $[M+H]^+$ 205.2.

*tert-butyl 8-(3-methoxy-3-oxopropyl)-4-oxo-3,4-dihydroquinoline-1(2H)-carboxylate*. As yellow liquid (41%). $^1H$ NMR (600 MHz, $CDCl_3$) δ 7.86 (d, *J* = 7.6 Hz, 1H), 7.44 (d, *J* = 7.4 Hz, 1H), 7.32-7.11 (m, 1H), 4.71-4.47 (m, 1H), 3.67 (s, 3H), 3.61-3.43 (m, 1H), 3.19-2.55 (m, 6H), 1.49 (s, 9H). ESI-LRMS (m/z) $[M+H]^+$ 334.2.

## 2. Induction of *vraX*$_{pro}$-*lux* expression and the growth curve of the new TarO inhibitors

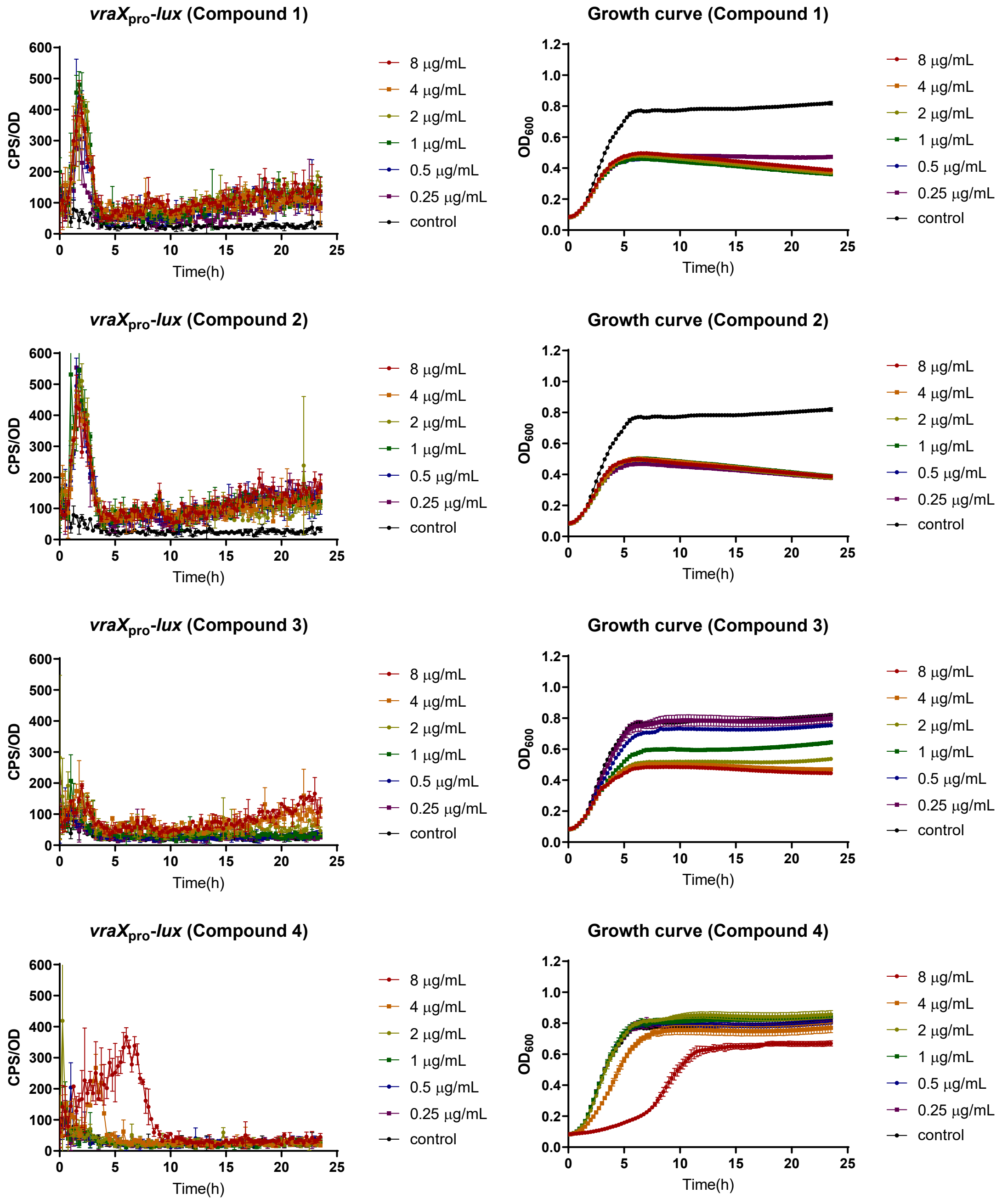

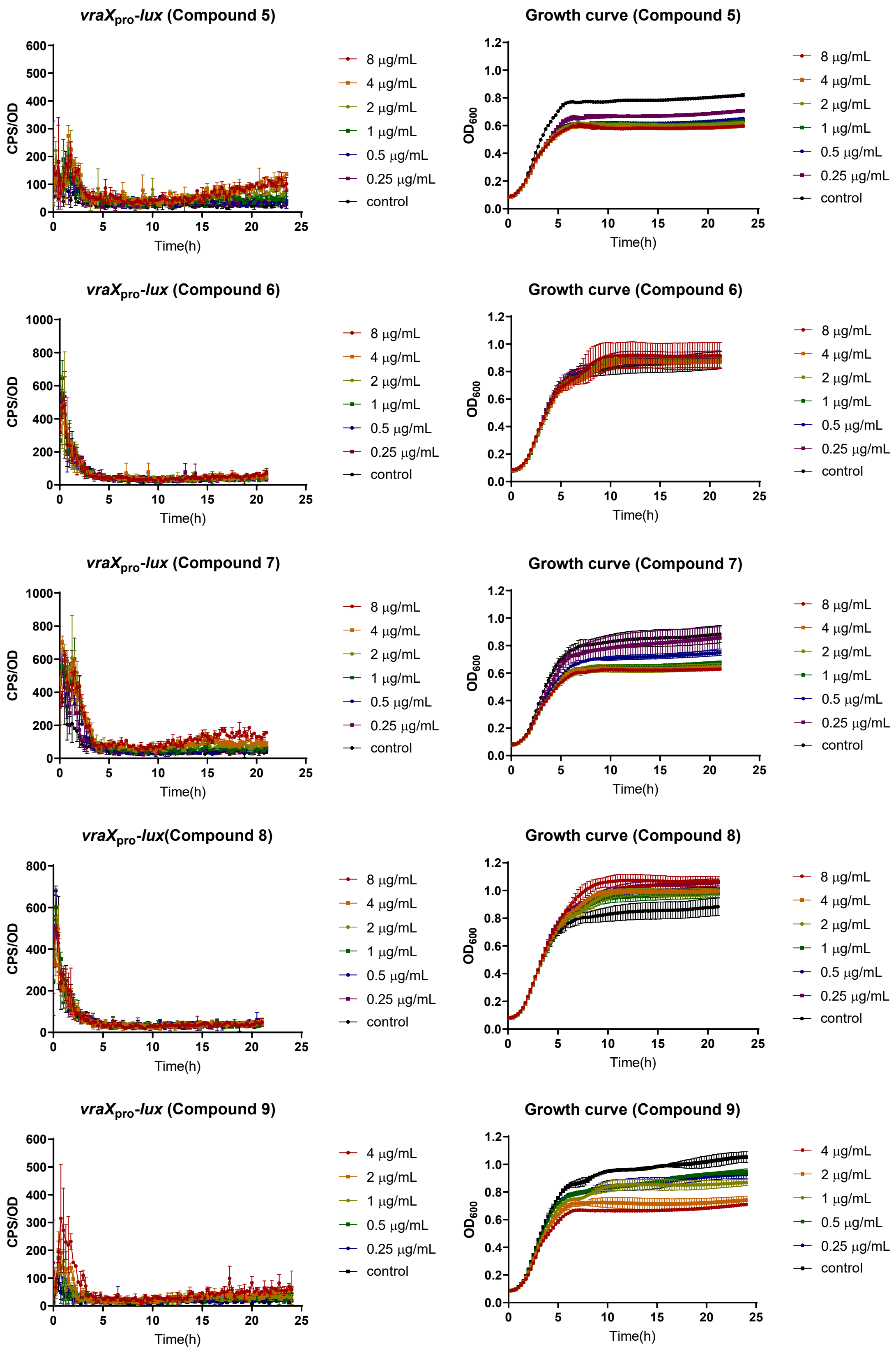
vraXpro-lux (Compound 5)
CPS/OD
Time(h)
Growth curve (Compound 5)
OD600
Time(h)
vraXpro-lux (Compound 6)
Growth curve (Compound 6)
vraXpro-lux (Compound 7)
Growth curve (Compound 7)
vraXpro-lux(Compound 8)
Growth curve (Compound 8)
8 µg/mL
4 µg/mL
2 µg/mL
1 µg/mL
0.5 µg/mL
0.25 µg/mL
control
vraXpro-lux (Compound 9)
Growth curve (Compound 9)
4 µg/mL
2 µg/mL
1 µg/mL
0.5 µg/mL
0.25 µg/mL
control

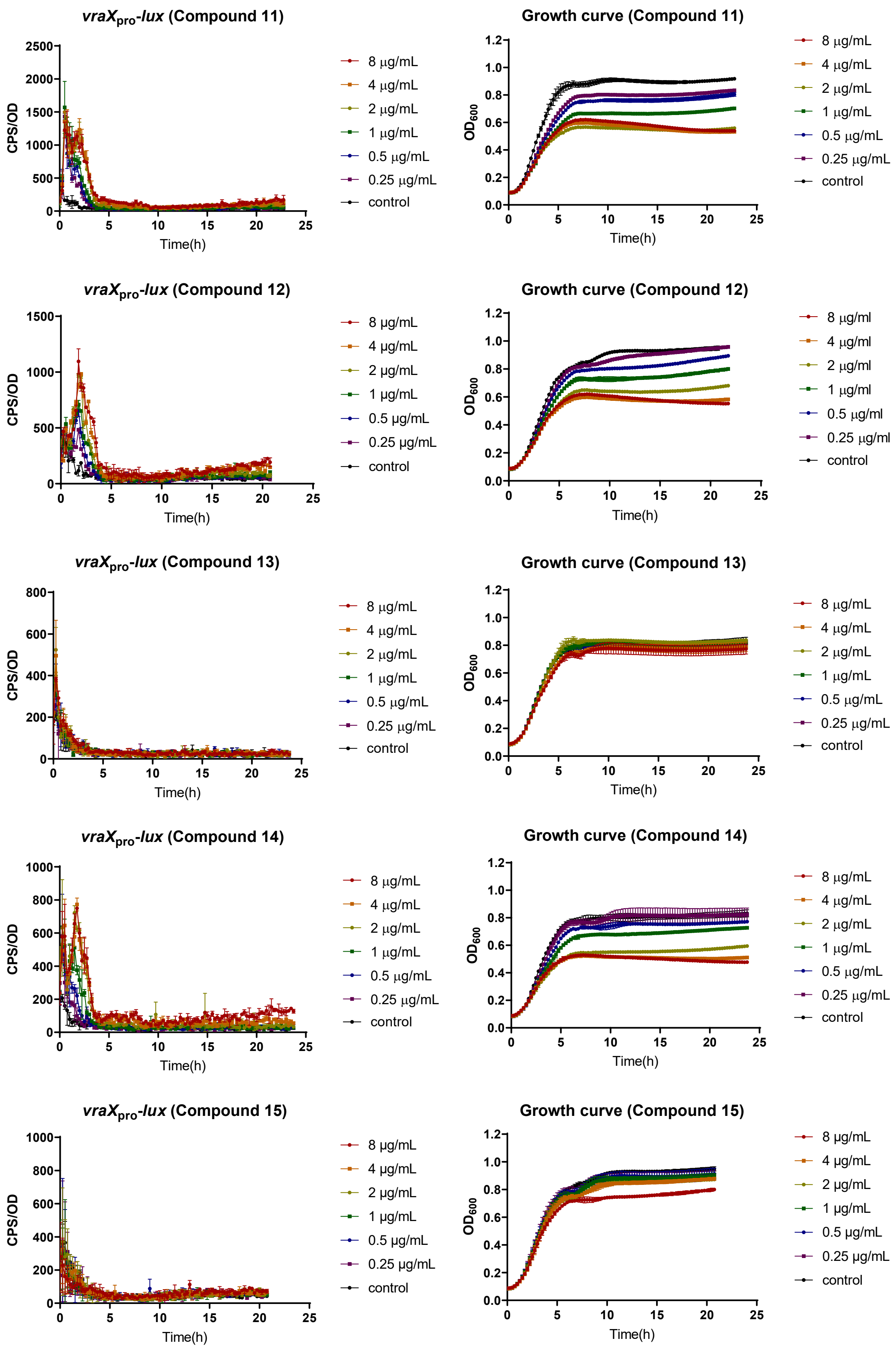
vraXpro-lux (Compound 11)
CPS/OD
Time(h)
8 µg/mL
4 µg/mL
2 µg/mL
1 µg/mL
0.5 µg/mL
0.25 µg/mL
control
Growth curve (Compound 11)
OD600
Time(h)
8 µg/mL
4 µg/mL
2 µg/mL
1 µg/mL
0.5 µg/mL
0.25 µg/mL
control
vraXpro-lux (Compound 12)
CPS/OD
Time(h)
8 µg/mL
4 µg/mL
2 µg/mL
1 µg/mL
0.5 µg/mL
0.25 µg/mL
control
Growth curve (Compound 12)
OD600
Time(h)
8 µg/ml
4 µg/ml
2 µg/ml
1 µg/ml
0.5 µg/ml
0.25 µg/ml
control
vraXpro-lux (Compound 13)
CPS/OD
Time(h)
8 µg/mL
4 µg/mL
2 µg/mL
1 µg/mL
0.5 µg/mL
0.25 µg/mL
control
Growth curve (Compound 13)
OD600
Time(h)
8 µg/mL
4 µg/mL
2 µg/mL
1 µg/mL
0.5 µg/mL
0.25 µg/mL
control
vraXpro-lux (Compound 14)
CPS/OD
Time(h)
8 µg/mL
4 µg/mL
2 µg/mL
1 µg/mL
0.5 µg/mL
0.25 µg/mL
control
Growth curve (Compound 14)
OD600
Time(h)
8 µg/mL
4 µg/mL
2 µg/mL
1 µg/mL
0.5 µg/mL
0.25 µg/mL
control
vraXpro-lux (Compound 15)
CPS/OD
Time(h)
8 µg/mL
4 µg/mL
2 µg/mL
1 µg/mL
0.5 µg/mL
0.25 µg/mL
control
Growth curve (Compound 15)
OD600
Time(h)
8 µg/mL
4 µg/mL
2 µg/mL
1 µg/mL
0.5 µg/mL
0.25 µg/mL
control

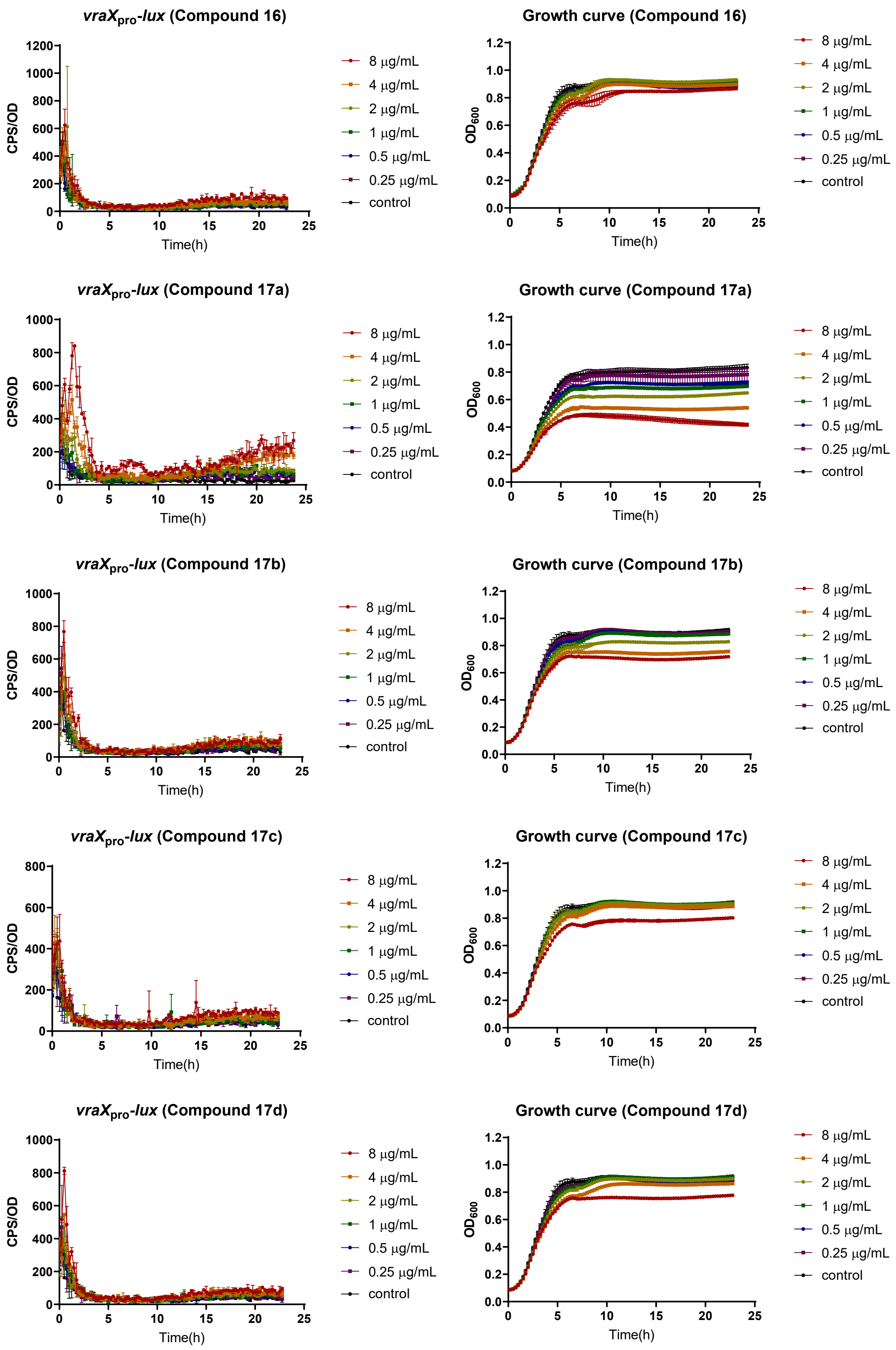
vraXpro-lux (Compound 16)
Growth curve (Compound 16)
vraXpro-lux (Compound 17a)
Growth curve (Compound 17a)
vraXpro-lux (Compound 17b)
Growth curve (Compound 17b)
vraXpro-lux (Compound 17c)
Growth curve (Compound 17c)
vraXpro-lux (Compound 17d)
Growth curve (Compound 17d)
CPS/OD
OD600
Time(h)
8 µg/mL
4 µg/mL
2 µg/mL
1 µg/mL
0.5 µg/mL
0.25 µg/mL
control

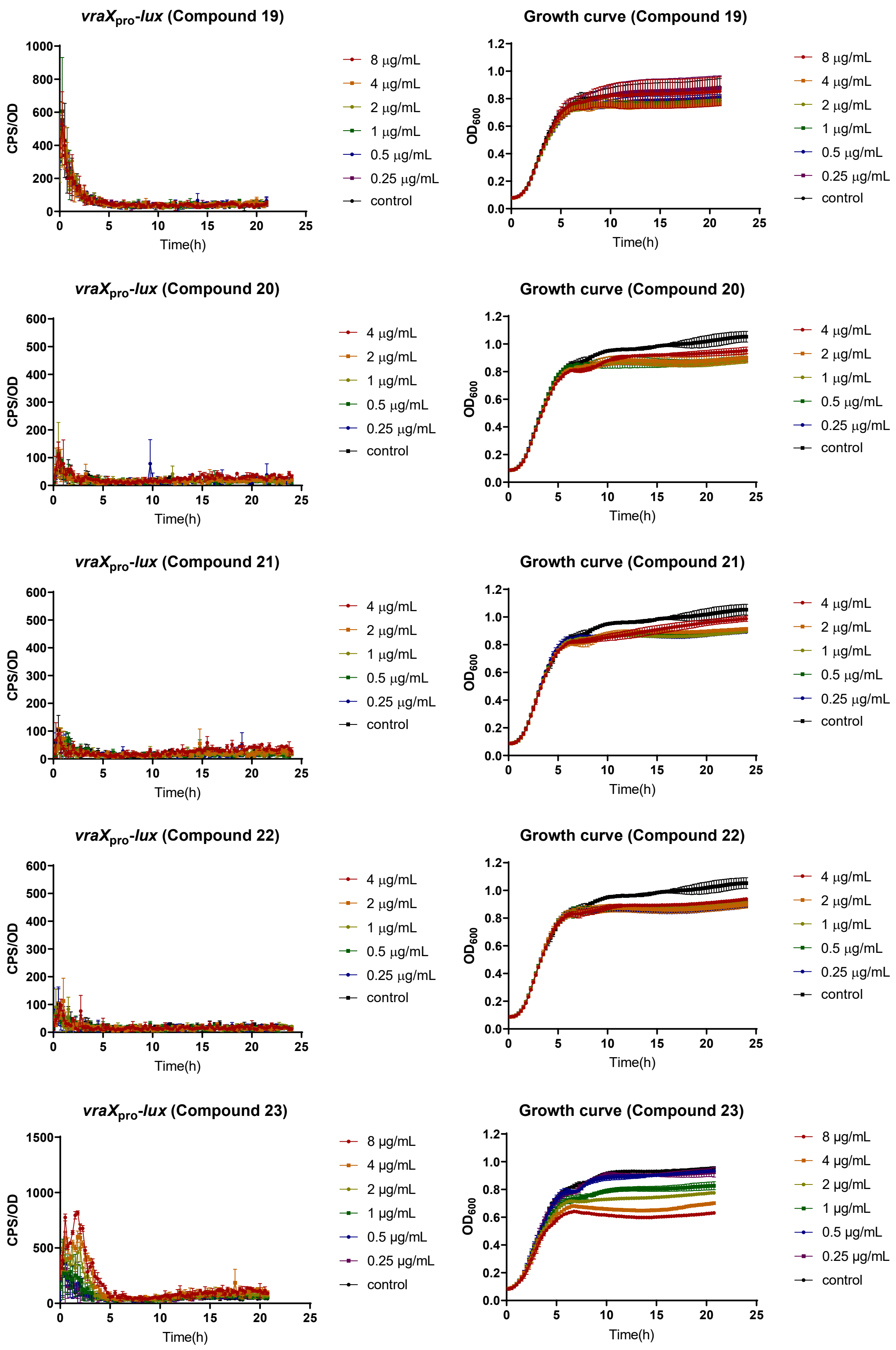
vraXpro-lux (Compound 19)
Growth curve (Compound 19)
vraXpro-lux (Compound 20)
Growth curve (Compound 20)
vraXpro-lux (Compound 21)
Growth curve (Compound 21)
vraXpro-lux (Compound 22)
Growth curve (Compound 22)
vraXpro-lux (Compound 23)
Growth curve (Compound 23)
CPS/OD
OD600
Time(h)
8 μg/mL
4 μg/mL
2 μg/mL
1 μg/mL
0.5 μg/mL
0.25 μg/mL
control

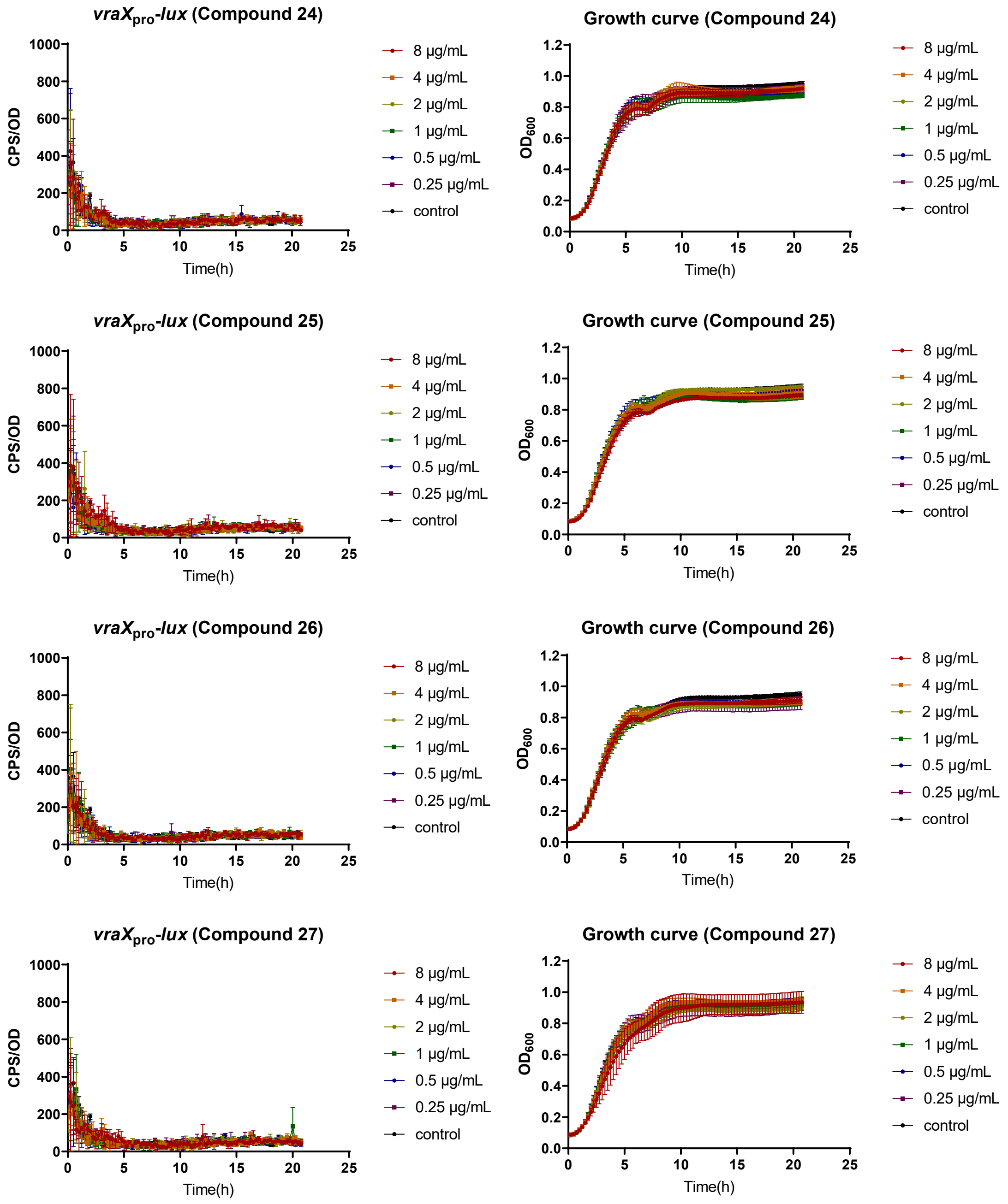


**Supplementary Figure 1:** TarO inhibitory activity of compounds. Data from n = 2 biological replicates and reported as the mean ± SD.

## 3. Doses-response curve of the new TarO inhibitors

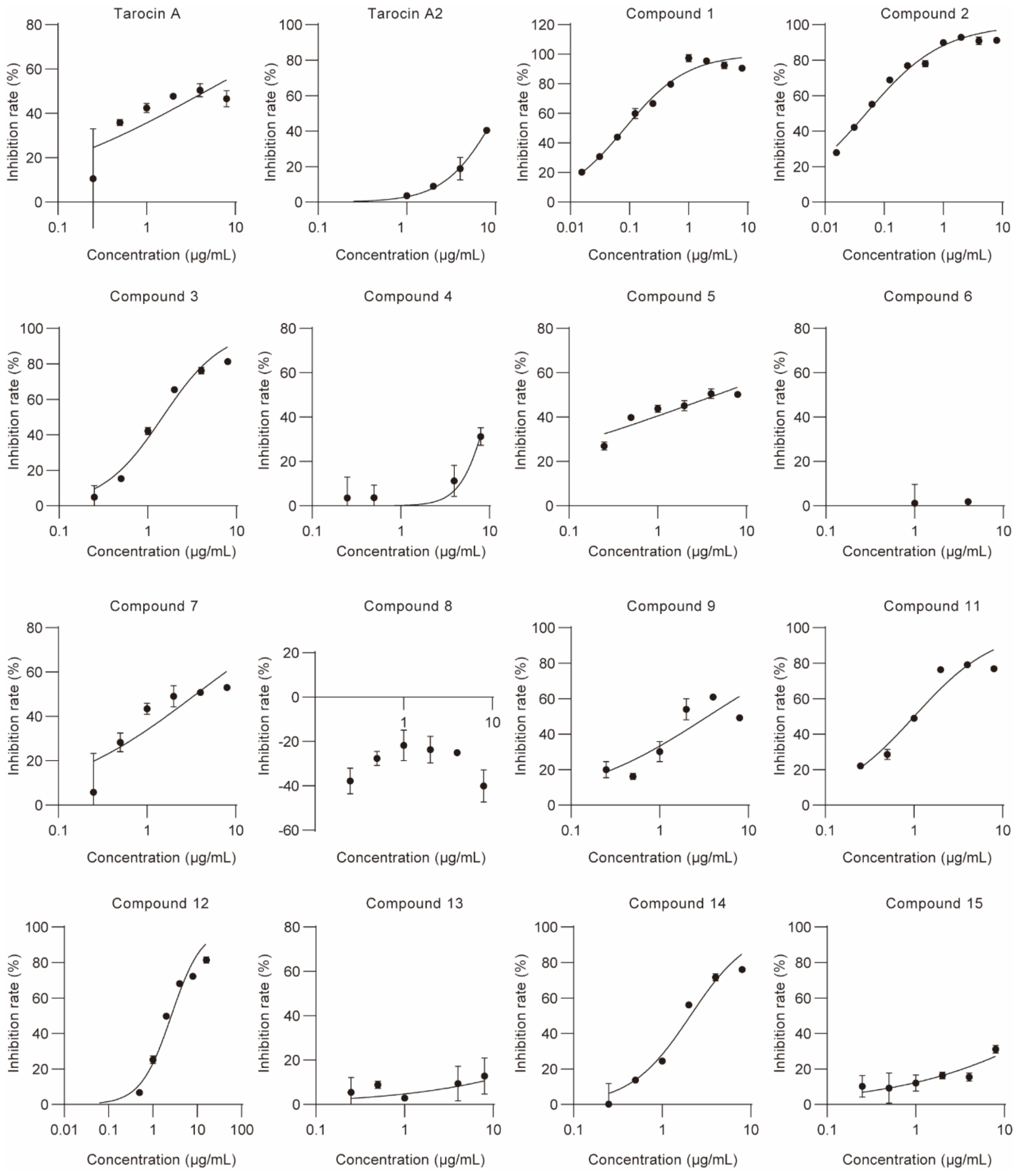

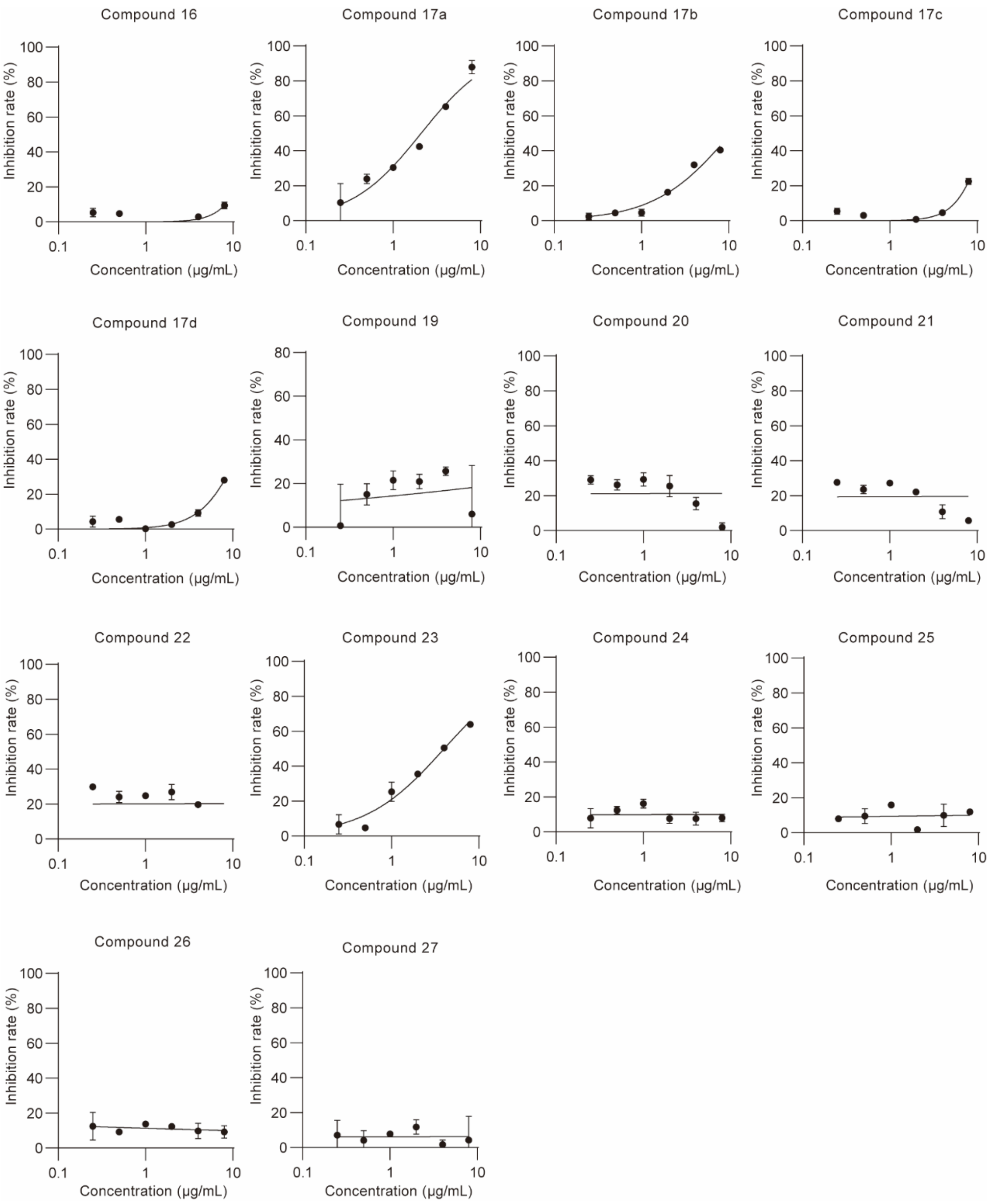


**Supplementary Figure 2:** Doses-response curve generated by different concentrations of the three compounds and TarO inhibition rate. Data was obtained from two biological replicates.

## 4. Supplementary Table 1: The minimum sensitization concentration of some representative compounds (USA300 LAC /oxacillin)

| Compounds | Minimum sensitization concentration (μg/mL)[a] | MIC (μg/mL)[b] Individual/Combined | Sensitization fold | FICI[c] |
|---|---|---|---|---|
| 1 | 0.06 | 64/8 | 8 | <0.13 |
| 2 | 0.06 | 64/8 | 8 | <0.13 |
| 5 | 0.25 | 64/8 | 8 | <0.13 |
| 11 | 0.03 | 64/16 | 4 | <0.13 |
| 14 | 0.25 | 64/8 | 8 | <0.13 |
| 17a | 4 | 64/8 | 8 | <0.15 |

[a] test range: 0.03-32 μg/mL

[b]MIC of β-lactams used alone or in combination with TarO inhibitors.

[c]FICI = ($MIC_{inhibitor}$ in combination / $MIC_{inhibitor}$ alone) + ($MIC_{antibiotic}$ in combination / $MIC_{antibiotic}$ alone).

## 5. Supplementary Table 2: The maximum sensitization concentration of some representative compounds (USA300 LAC /oxacillin)

| Compounds | Maximum sensitization concentration (μg/mL)[a] | MIC (μg/mL)[b] Individual/Combined | Sensitization fold | FICI[c] |
|---|---|---|---|---|
| 1 | 0.25 | 64/4 | 16 | <0.07 |
| 2 | 1 | 64/2 | 32 | <0.04 |
| 5 | 0.25 | 64/8 | 8 | <0.13 |
| 11 | 0.5 | 64/4 | 16 | <0.07 |
| 14 | 2 | 64/4 | 16 | <0.08 |
| 17a | 32 | 64/0.06 | 1024 | <0.13 |

[a] test range: 0.03-32 μg/mL

[b]MIC of β-lactams used alone or in combination with TarO inhibitors.

[c]FICI = ($MIC_{inhibitor}$ in combination / $MIC_{inhibitor}$ alone) + ($MIC_{antibiotic}$ in combination / $MIC_{antibiotic}$ alone).

## 6. Supplementary Table 3: Preliminary liver microsomal stability testing of TarO inhibitors.

| Compounds | Species | $T_{1/2}$ (min) | Clint In Vitro (mL/min/gprot) | MF (%)[a] | Comments |
|---|---|---|---|---|---|
| **10** | Mouse | 1.18 | 1779 | 2.44 | poor |
| **18a** | Mouse | 0.69 | 3051 | 1.44 | poor |
| **18b** | Mouse | 1.66 | 1267 | 3.39 | poor |

[a] MF: metabolic bioavailability.

Microsomes in 0.1 M TRIS buffer pH 7.4 (final concentration 0.33 mg/mL), co-factor $MgCl_2$ (final concentration 5 mM) and tested compound (final concentration 0.1 μM, co-solvent 0.01% DMSO and 0.005% Bovin serum albumin BSA) were incubated at 37°C for 10 min. The reaction was started by the addition of NADPH (final concentration 1 mM). Aliquots were sampled at 0, 5, 15, 30 and 60 min respectively and methanol (cold in wet ice) was added to terminate the reaction. After centrifugation (4000rpm, 5 min), samples were then analyzed by LC-MS/MS.

## 7. NMR Spectra of the compounds

$^{1}$H NMR of Compound **1**

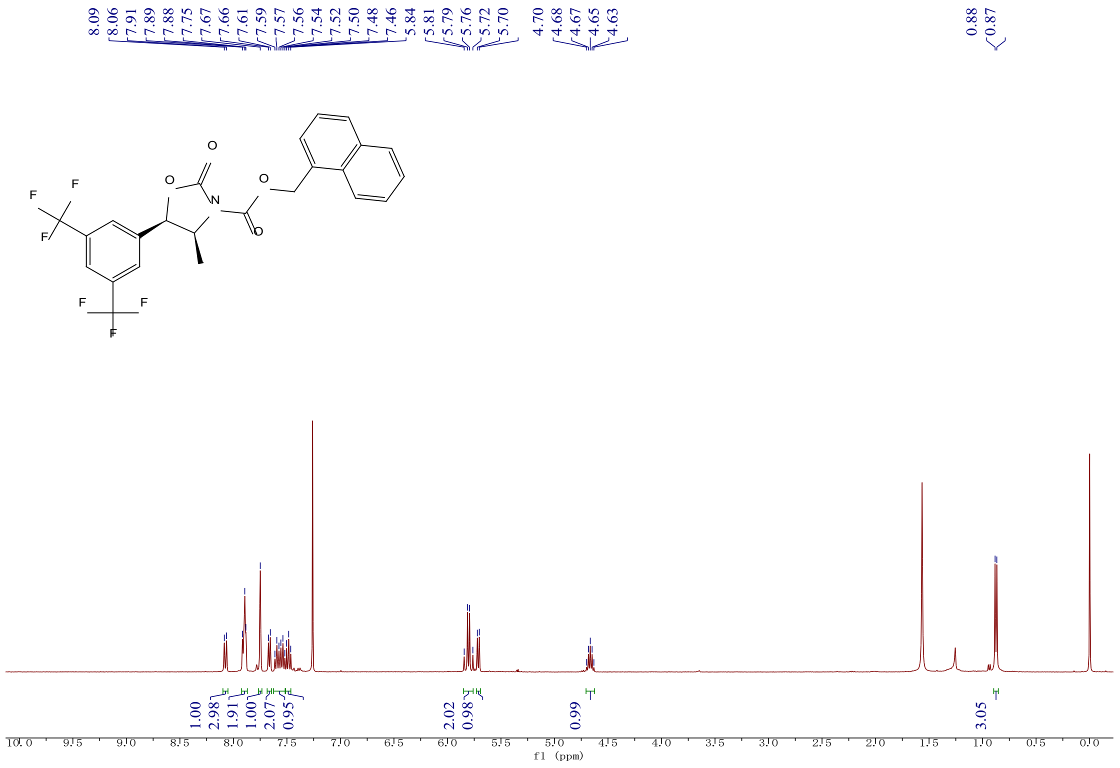


$^{13}$C NMR of Compound **1**

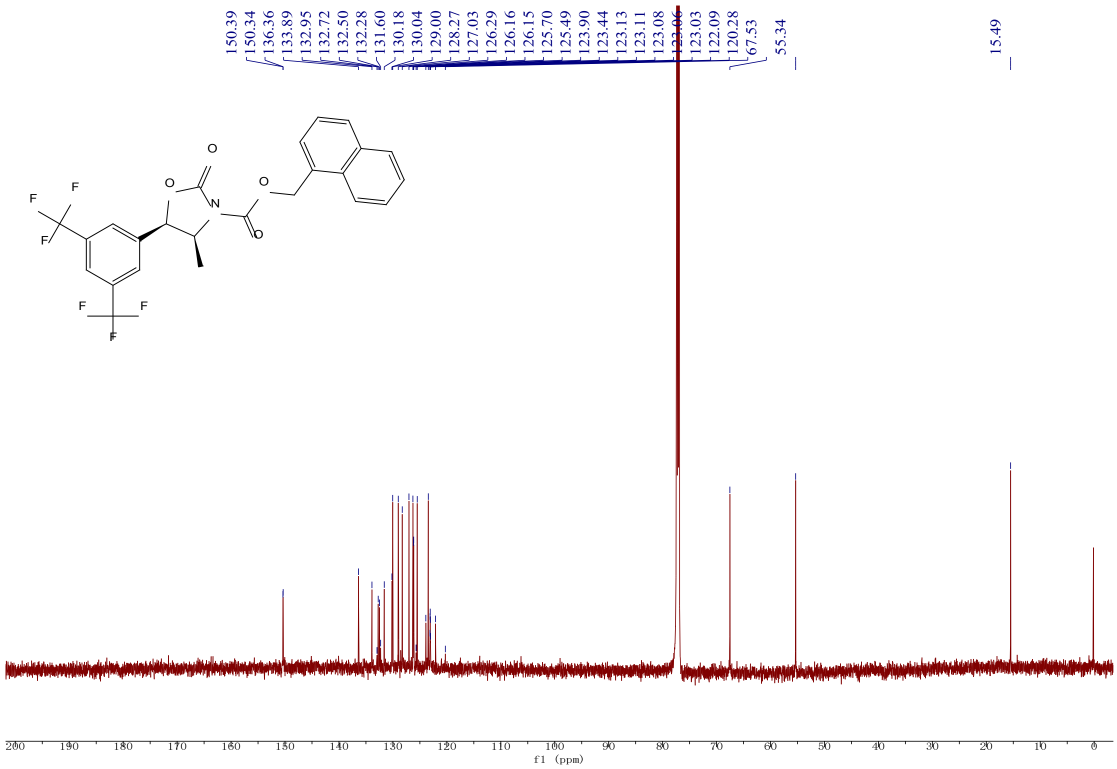


¹H NMR of Compound **2**

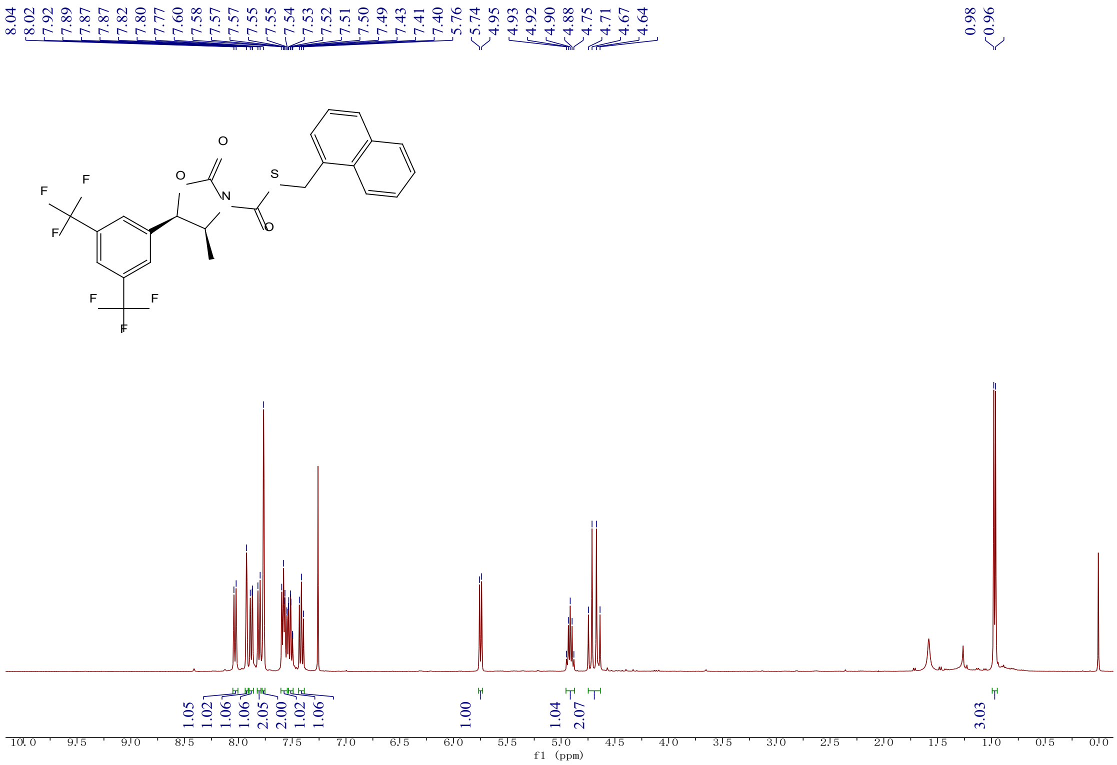


¹³C NMR of Compound **2**

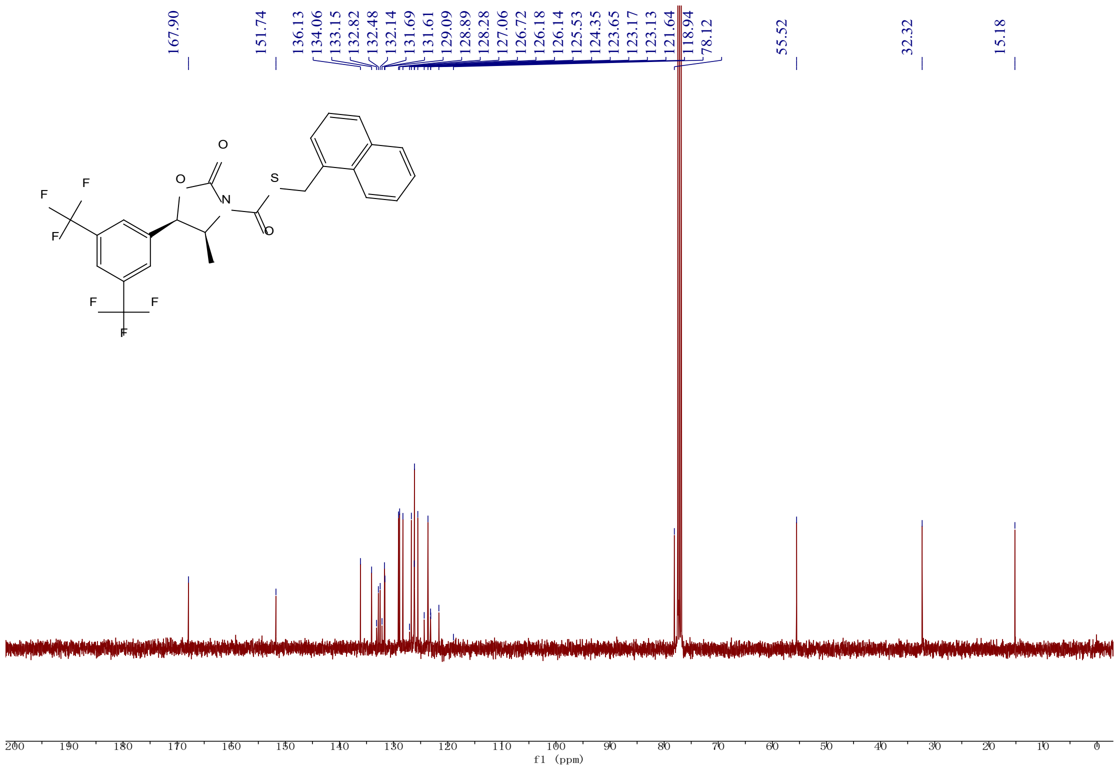


$^{1}$H NMR of Compound **3**

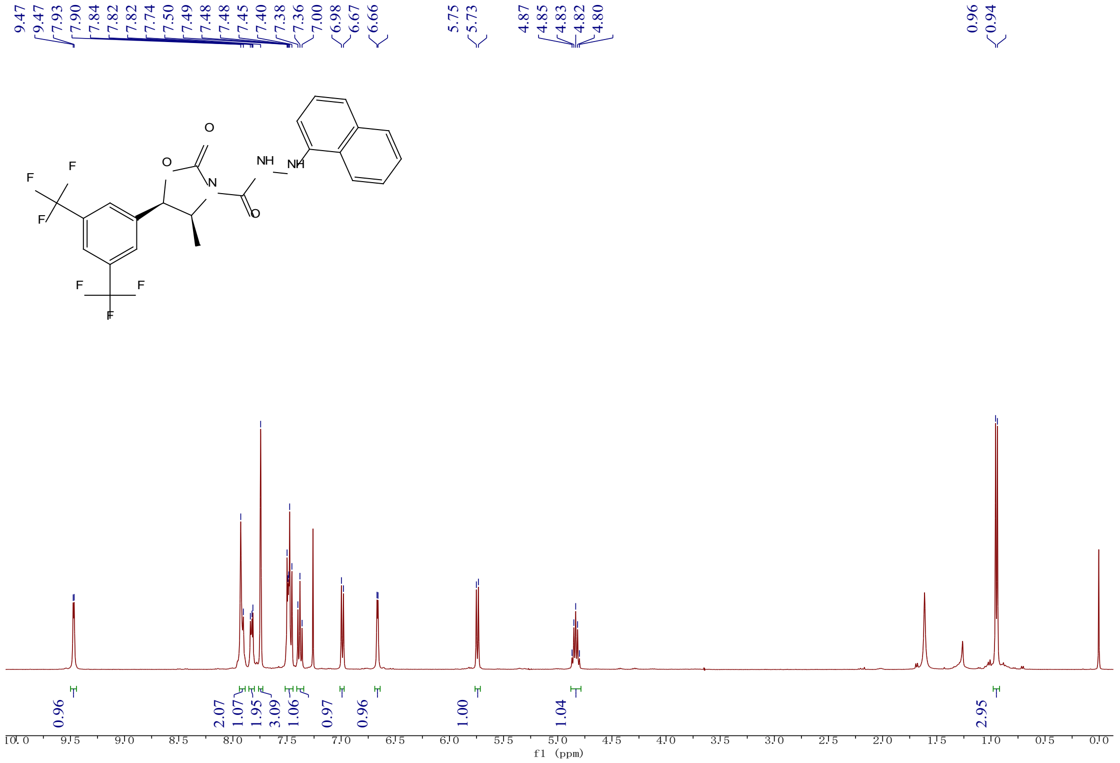


$^{13}$C NMR of Compound **3**

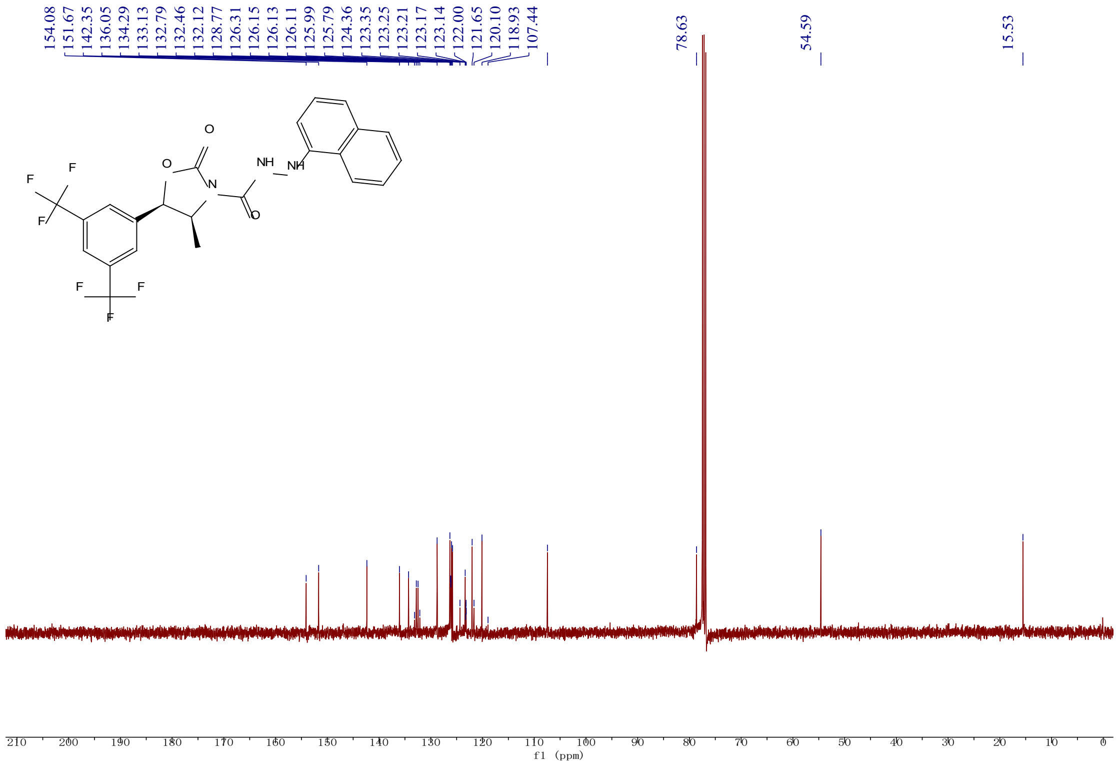


$^{1}$H NMR of Compound **4**

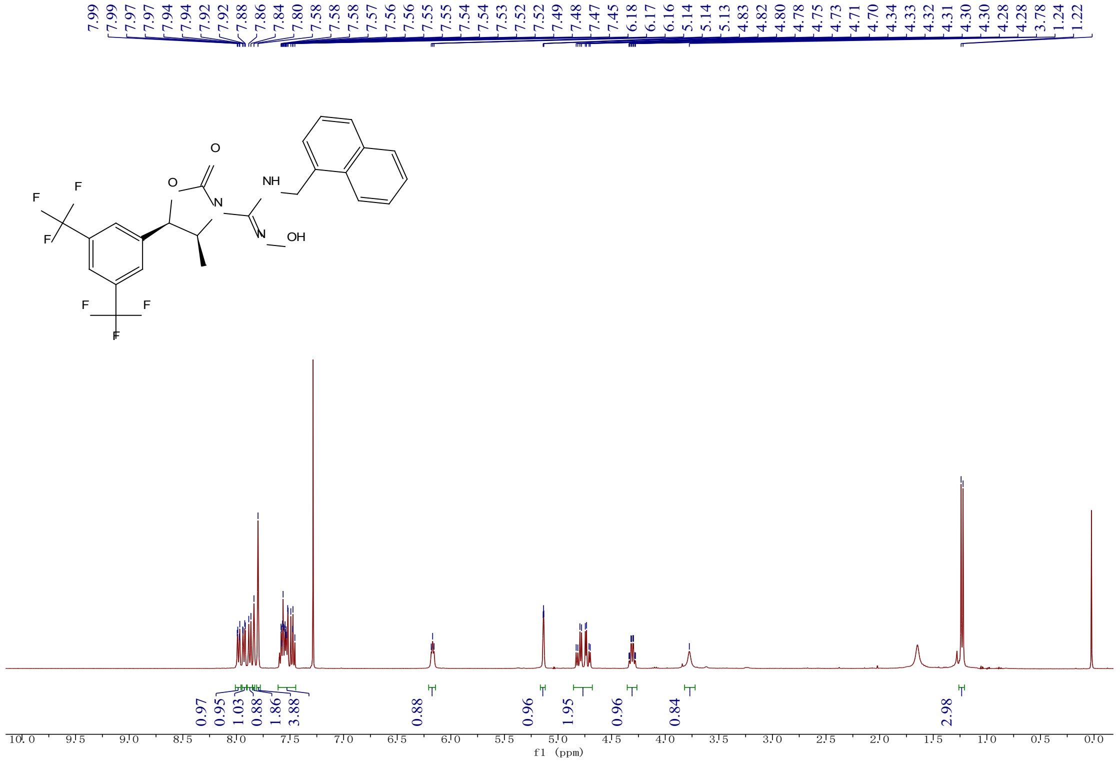

$^{13}$C NMR of Compound **4**

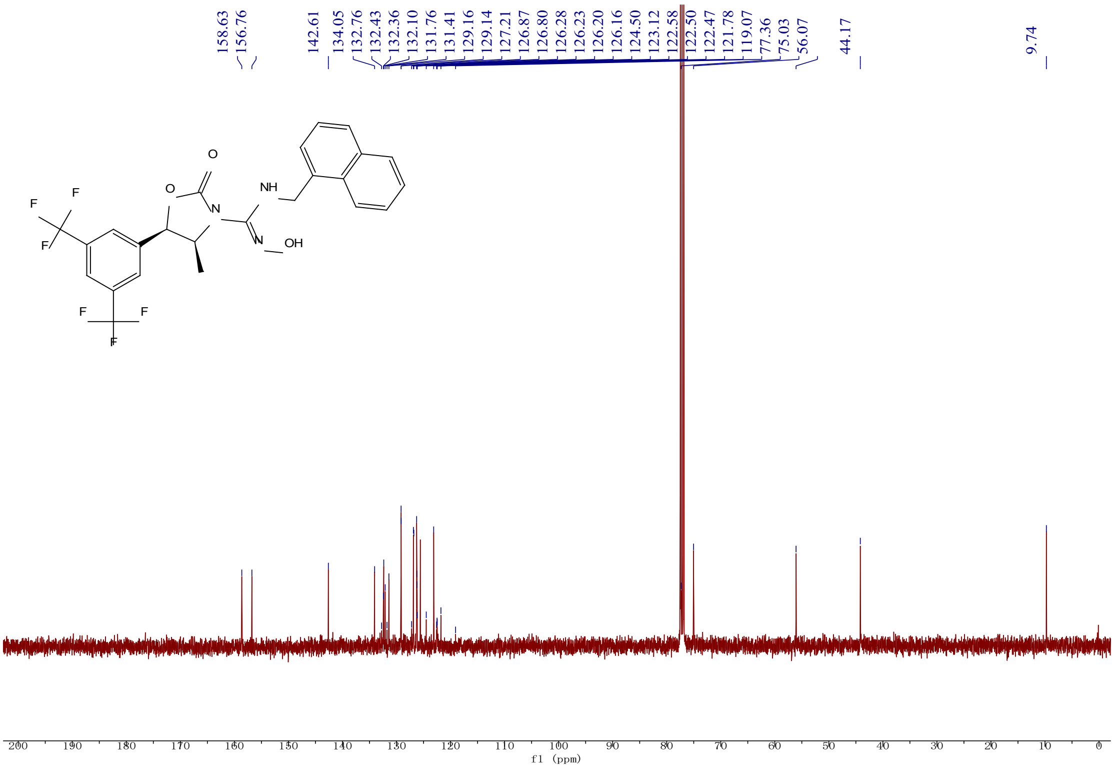


$^{1}$H NMR of Compound **5**

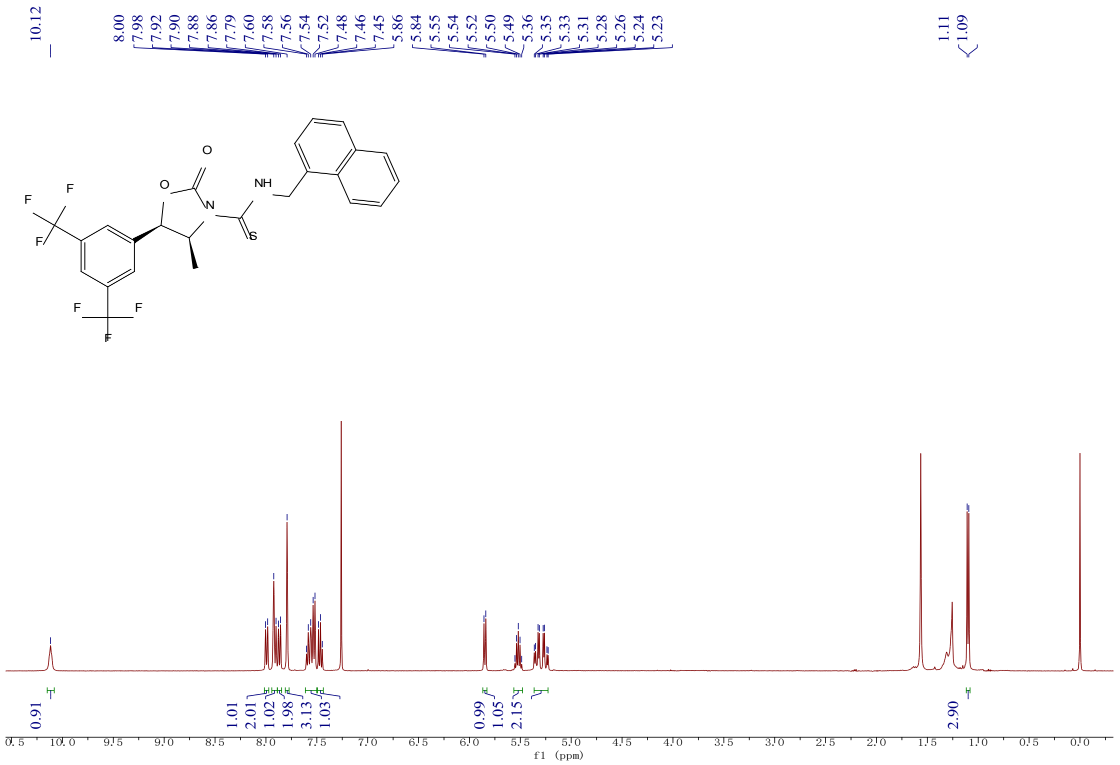


$^{13}$C NMR of Compound **5**

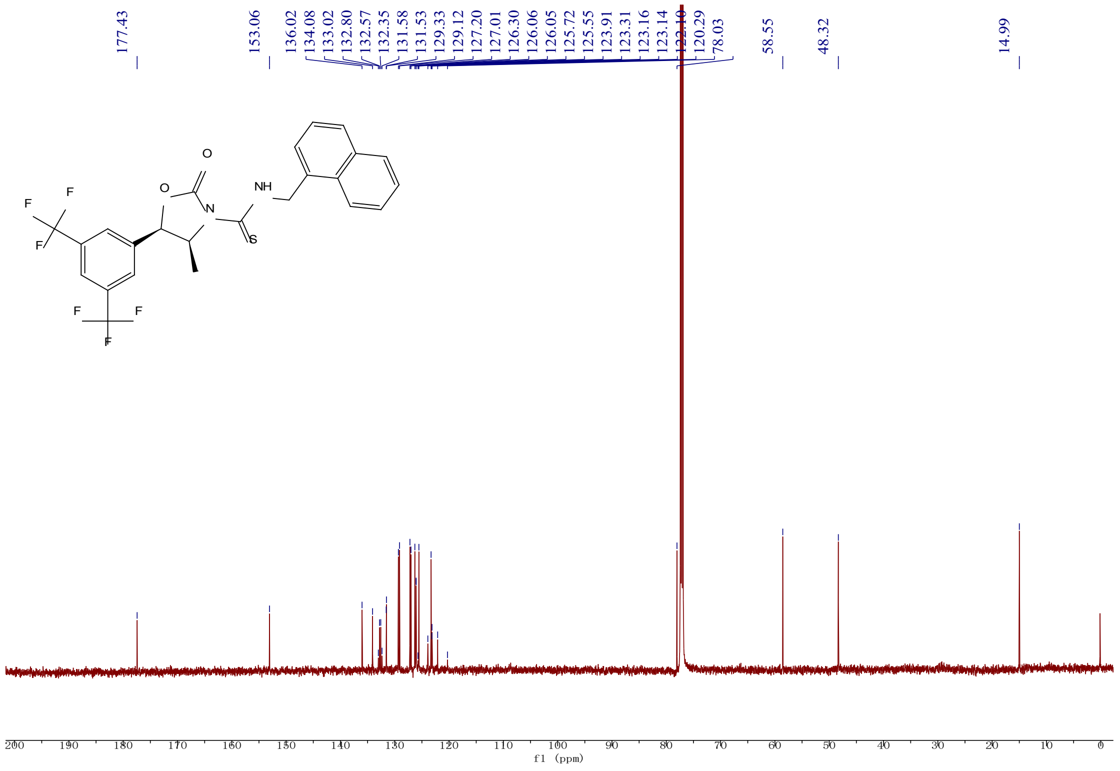

$^1$H NMR of Compound **6**

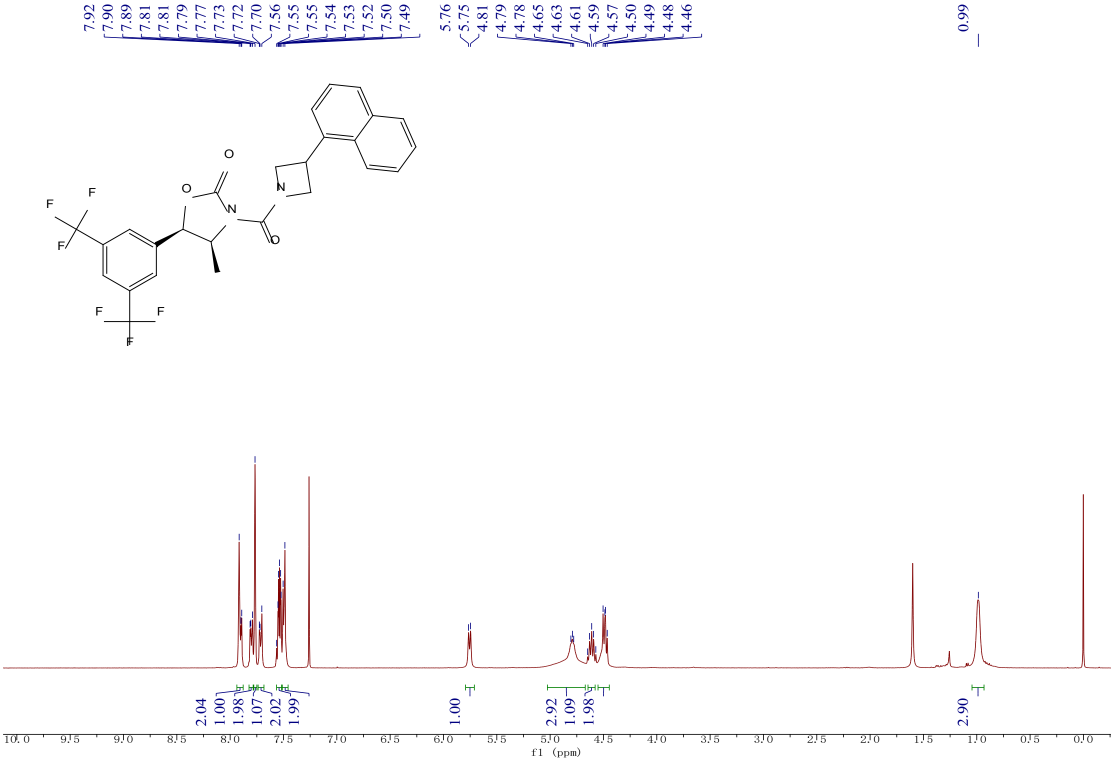


$^{13}$C NMR of Compound **6**

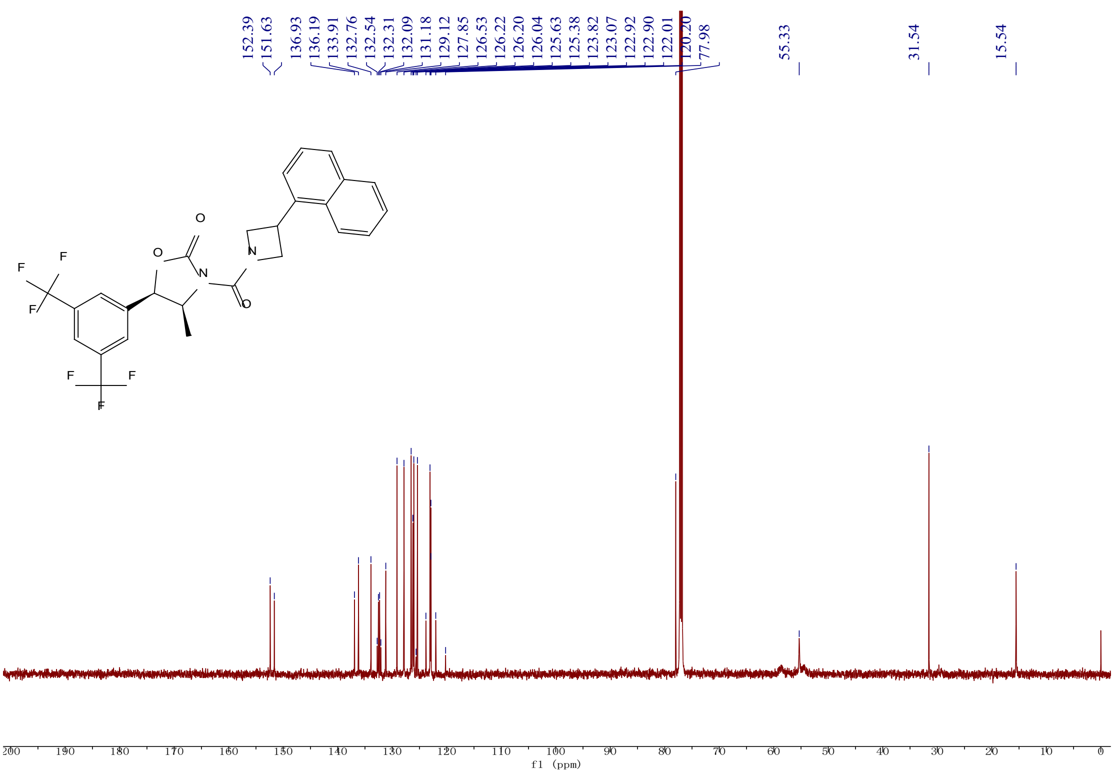

$^{1}$H NMR of Compound **7**

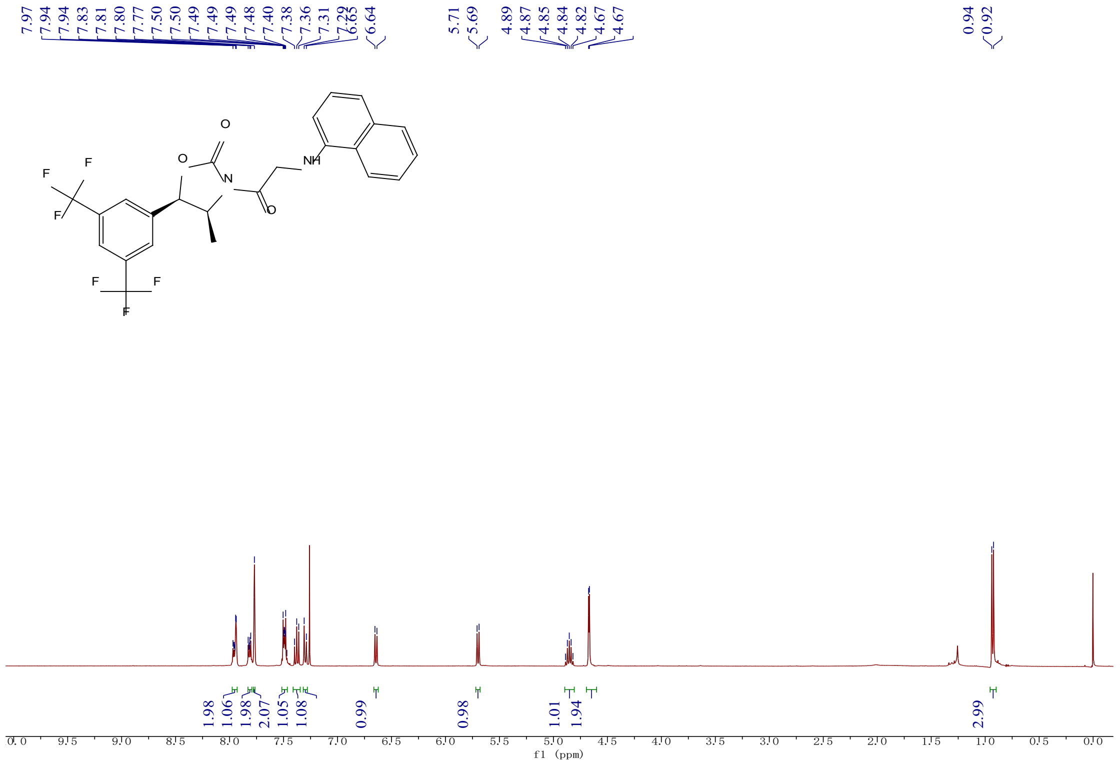

$^{13}$C NMR of Compound **7**

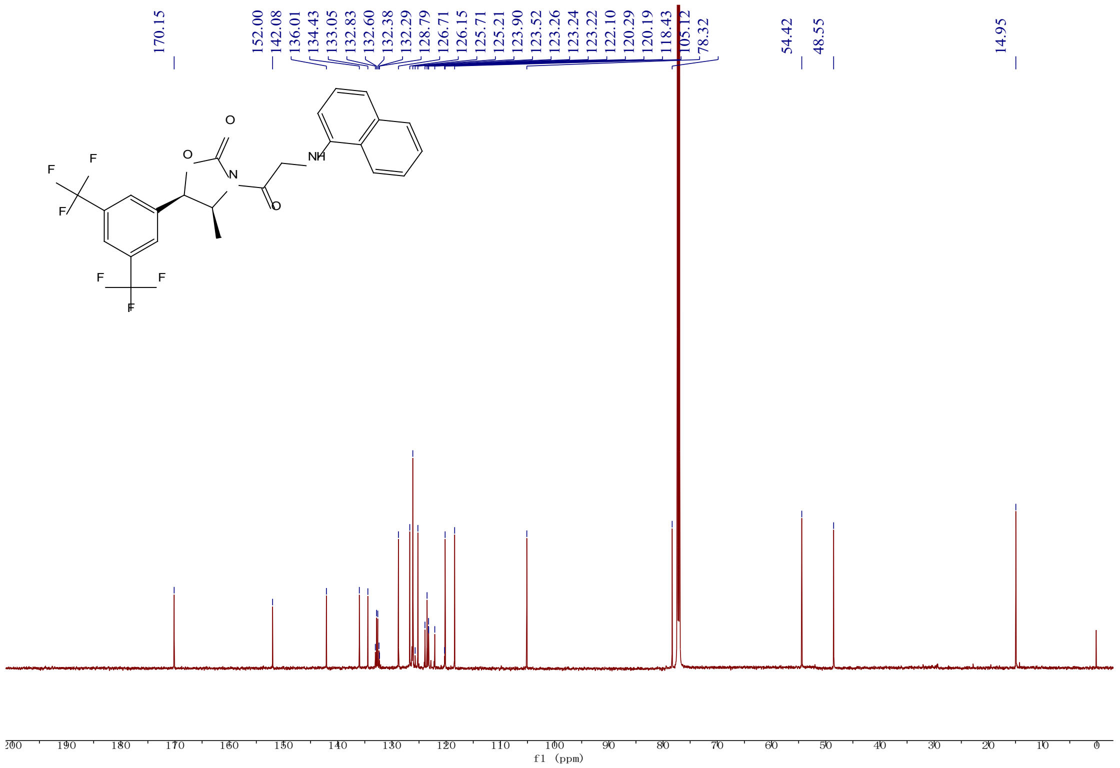

$^{1}$H NMR of Compound **8**

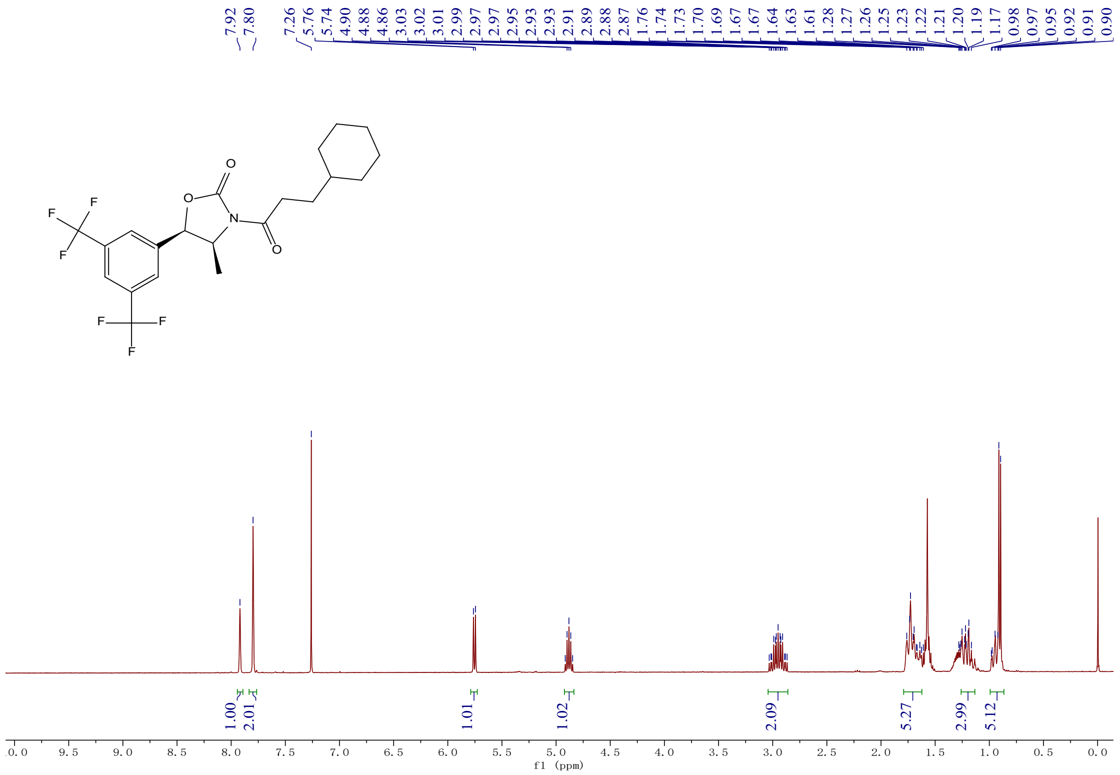


$^{13}$C NMR of Compound **8**

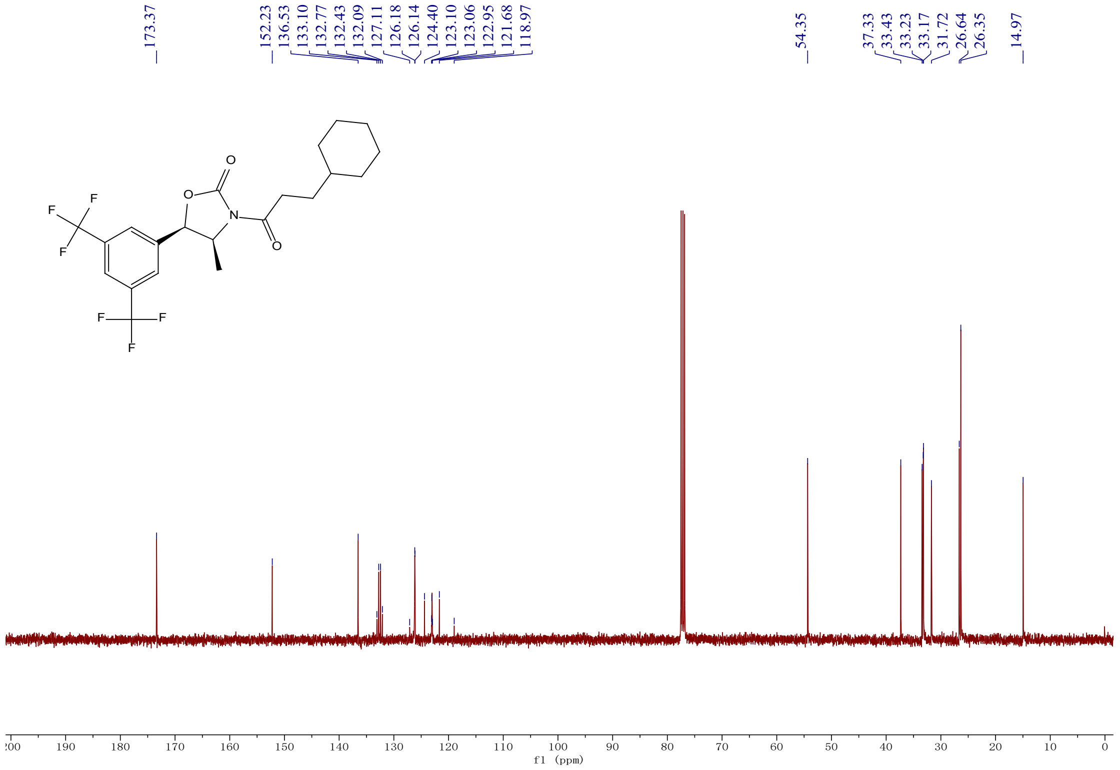

## $^{1}$H NMR of Compound **9**

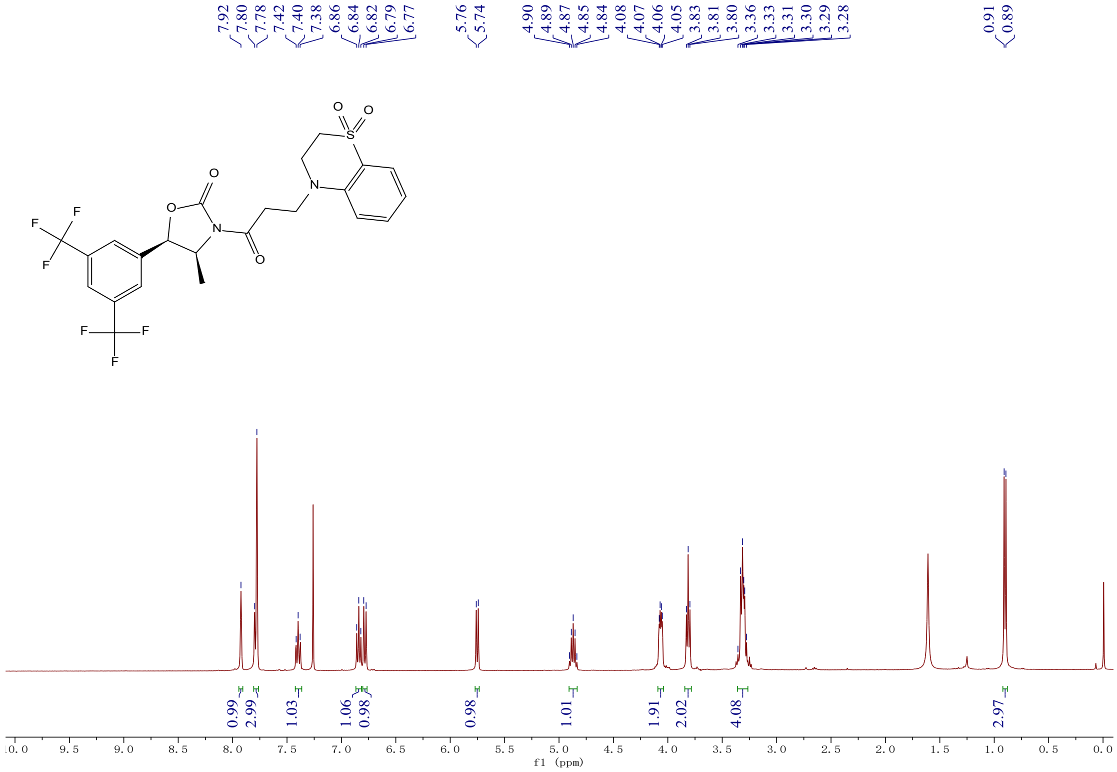


## $^{13}$C NMR of Compound **9**

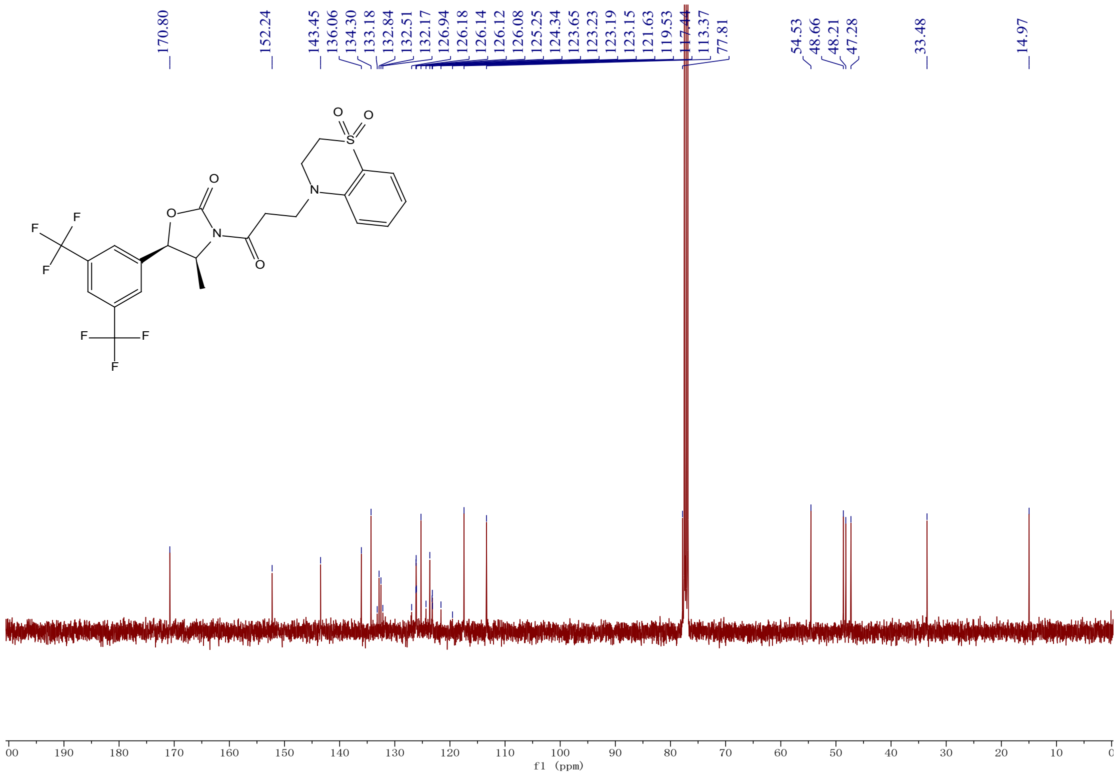

$^{1}$H NMR of Compound **10**

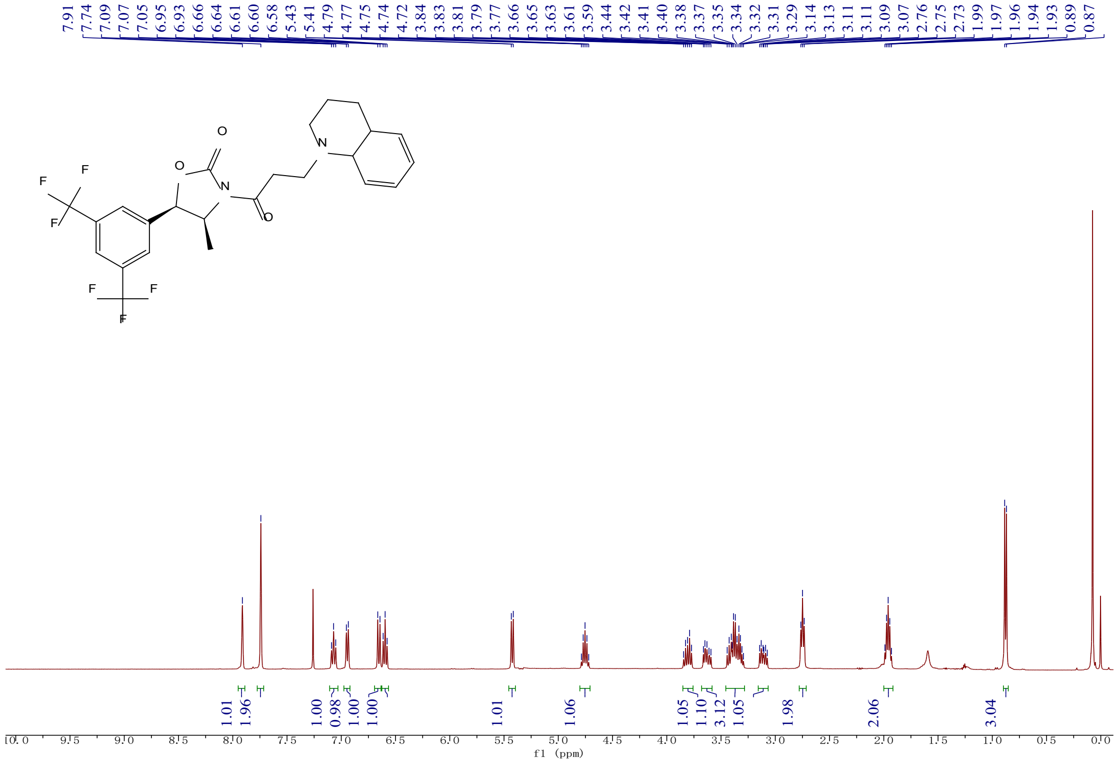


$^{13}$C NMR of Compound **10**

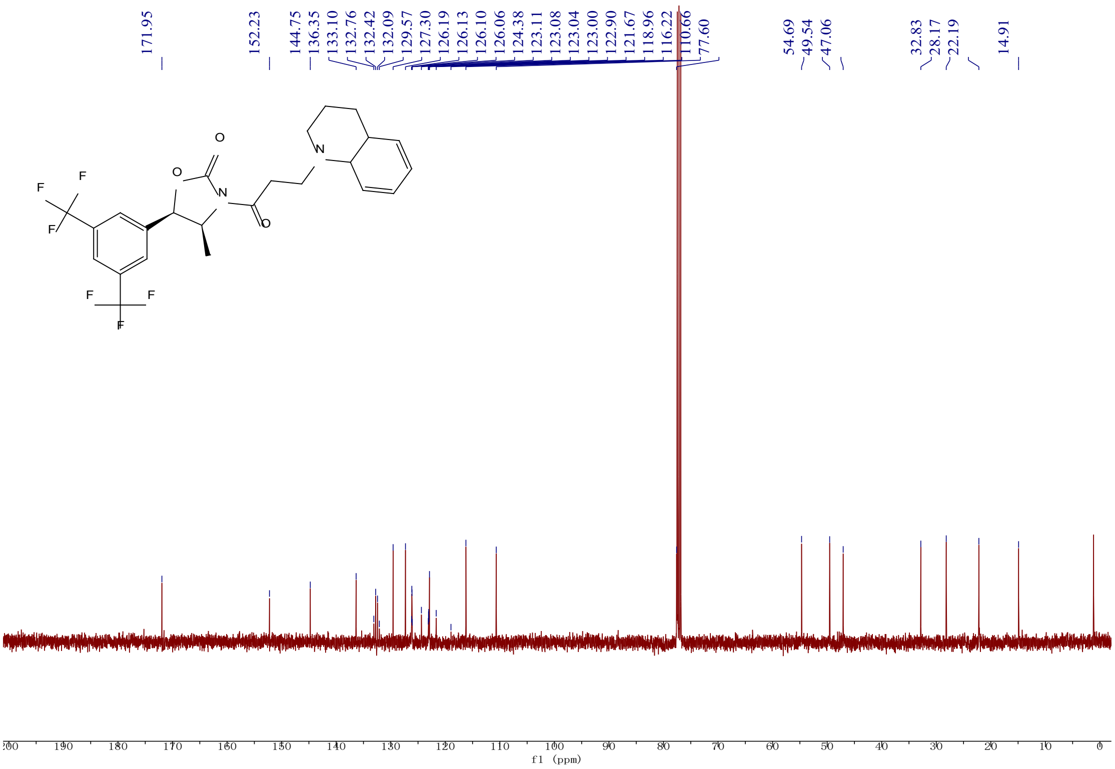


<sup></sup>

$^{1}$H NMR of Compound **11**

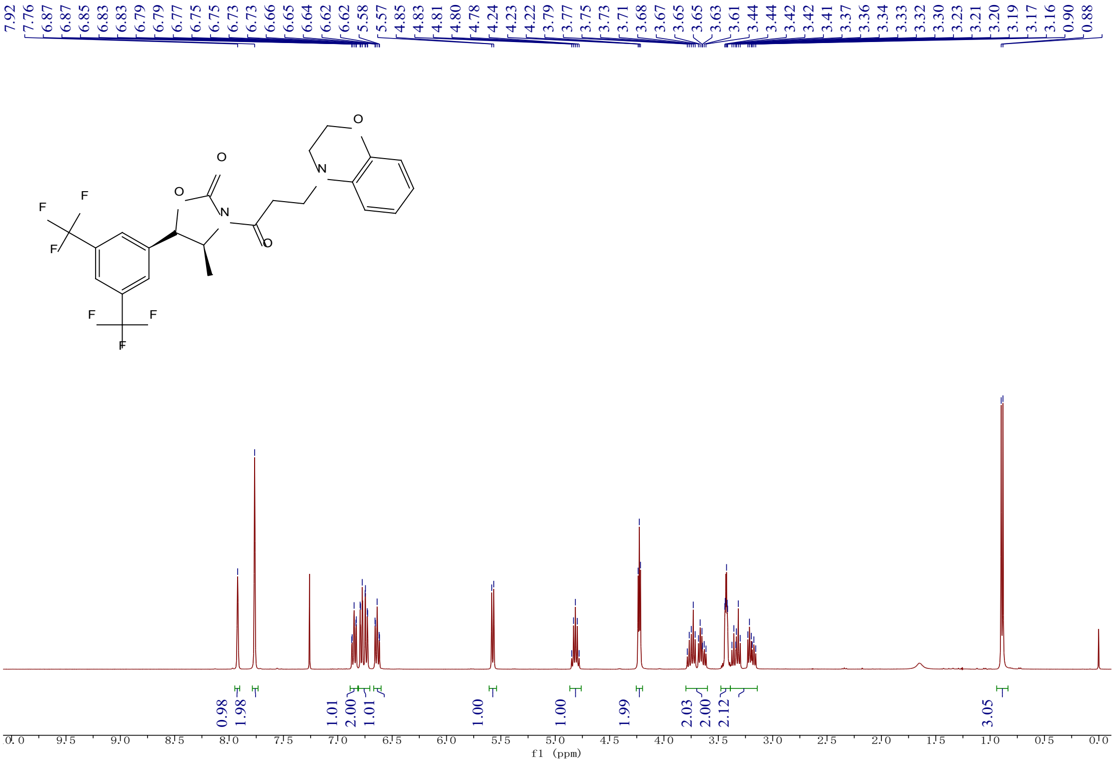

$^{13}C$ NMR of Compound **11**

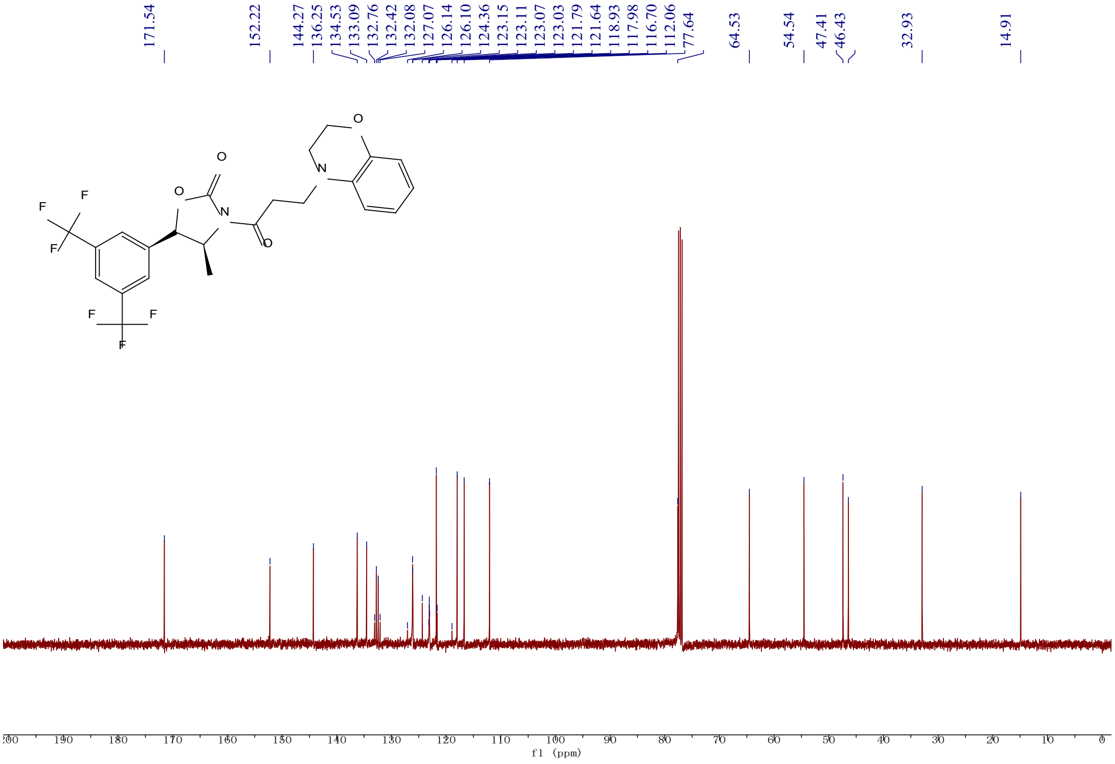


$^{1}H$ NMR of Compound **12**

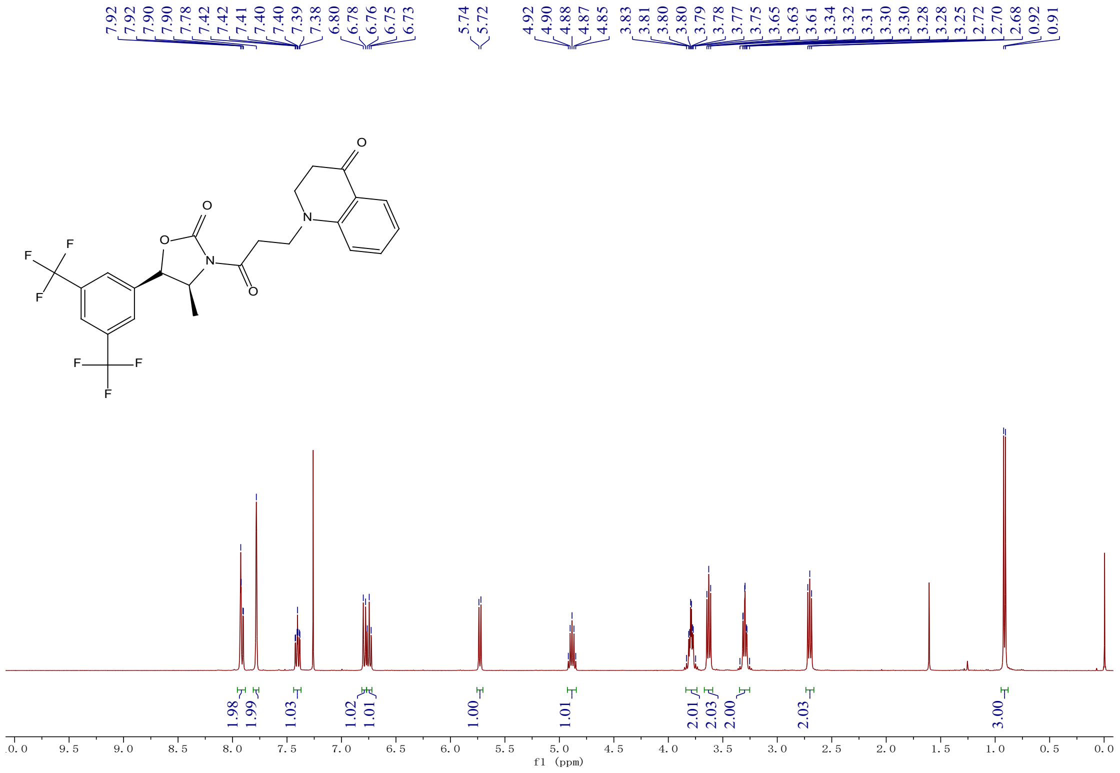


<sup></sup>

## $^{13}$C NMR of Compound **12**

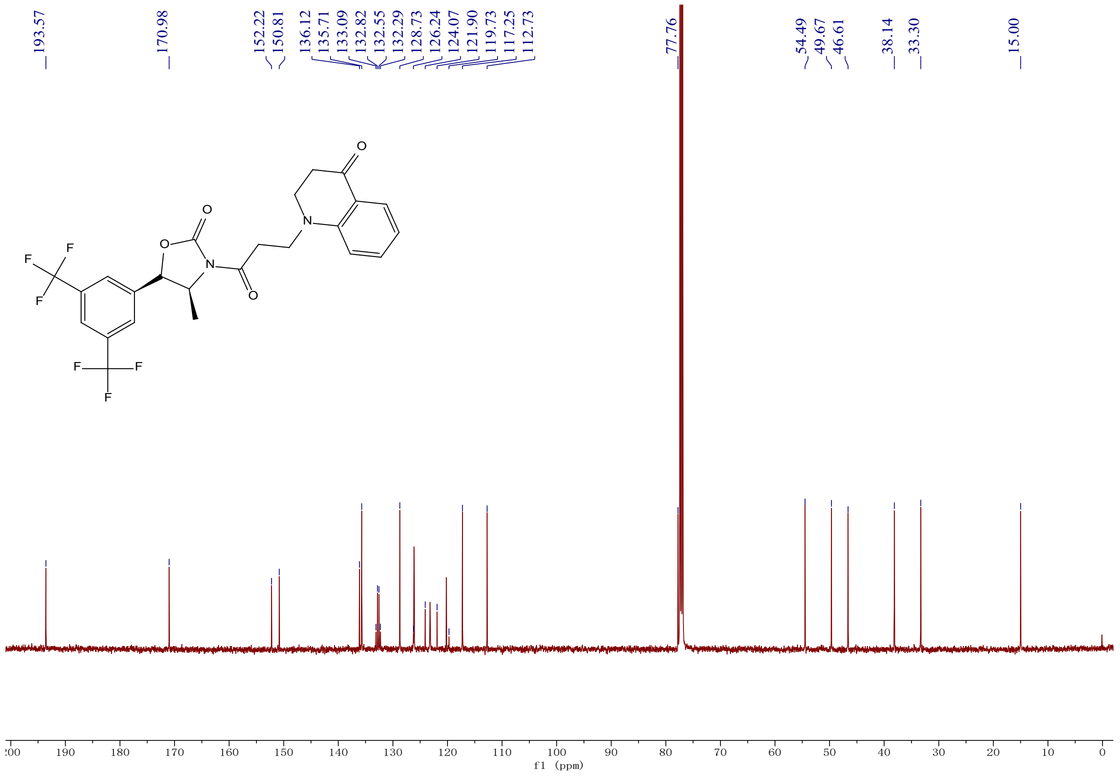

$^{1}$H NMR of Compound **13**

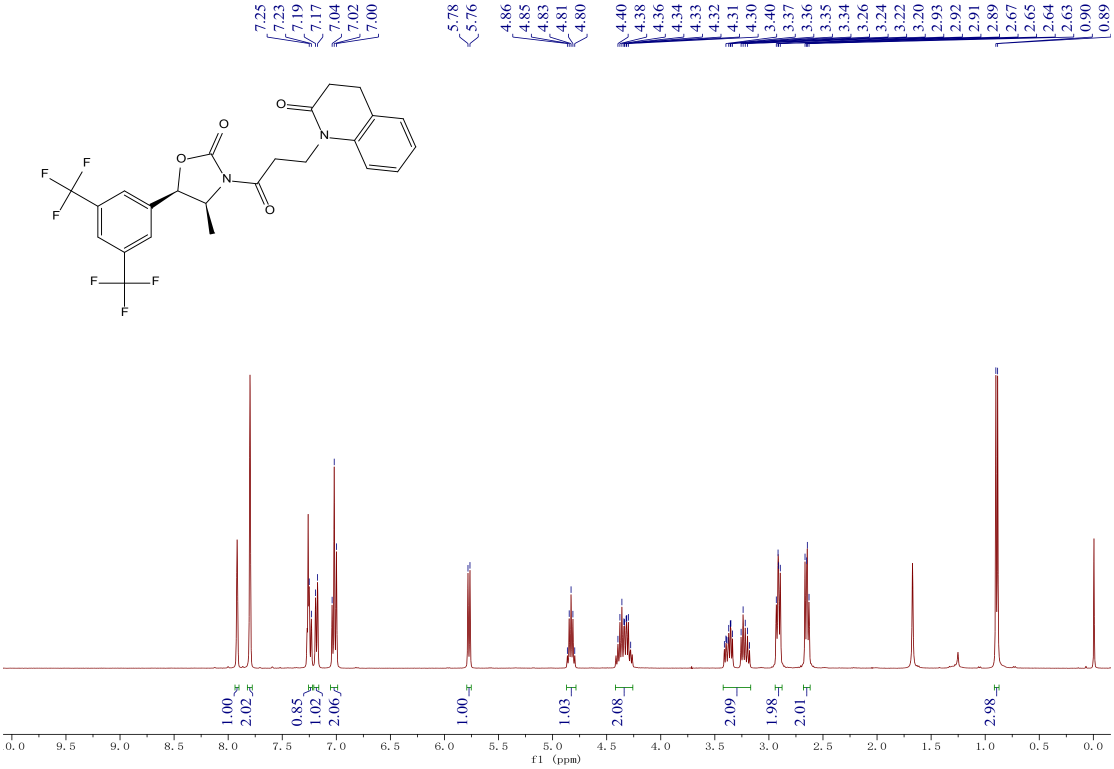


$^{13}$C NMR of Compound **13**

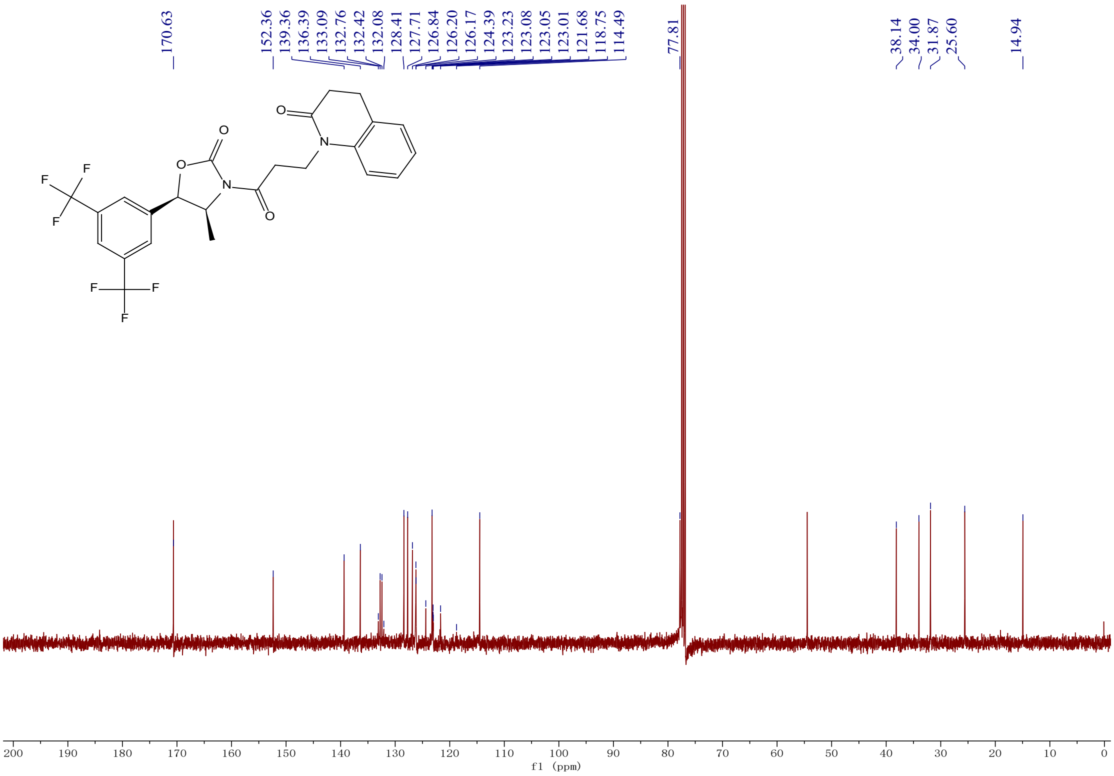

$^{1}$H NMR of Compound **14**

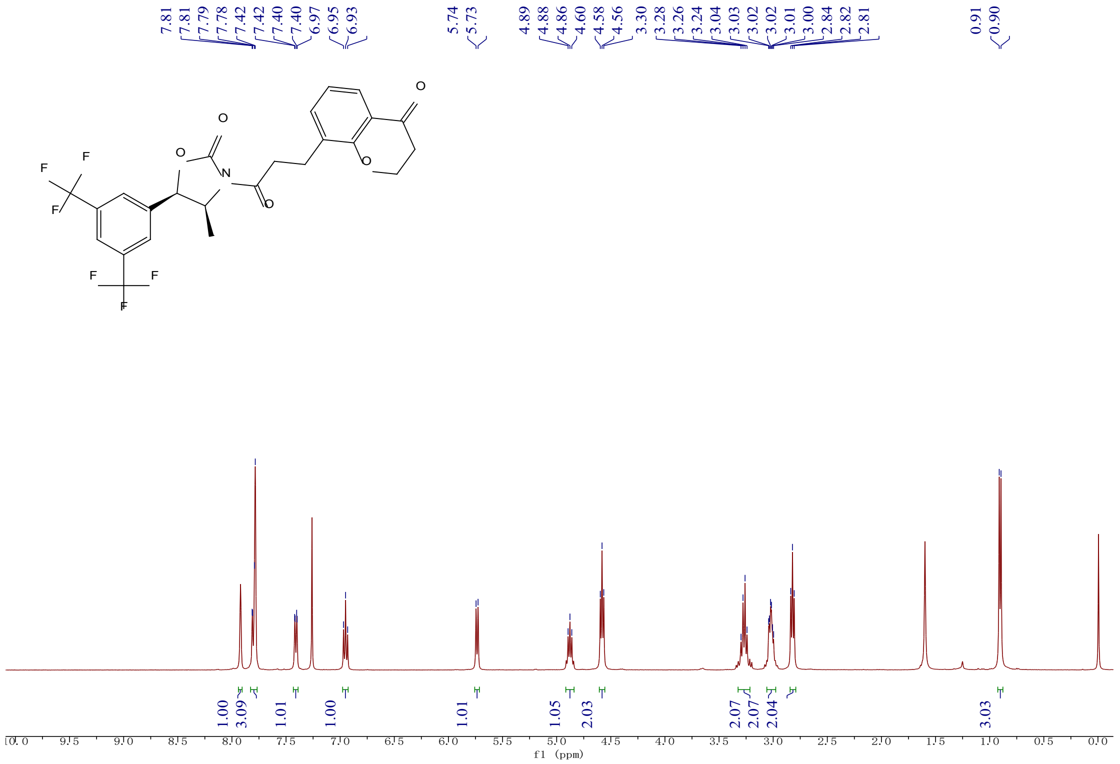

$^{13}$C NMR of Compound **14**

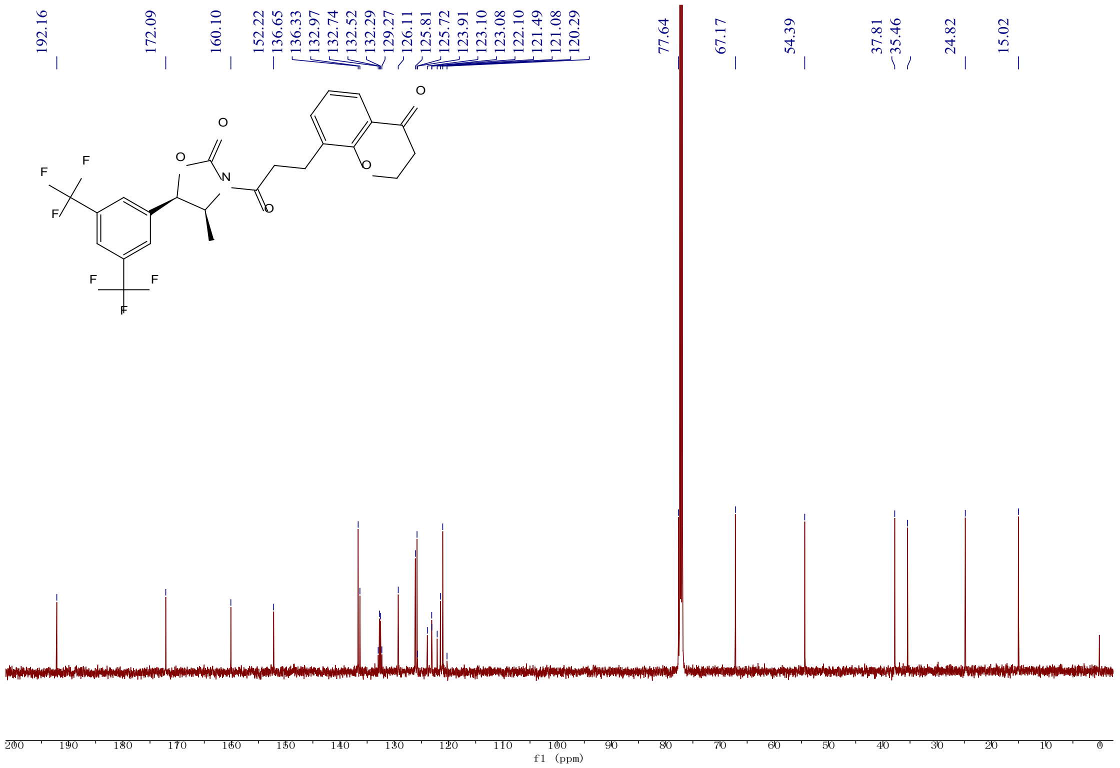

$^{1}$H NMR of Compound **15**

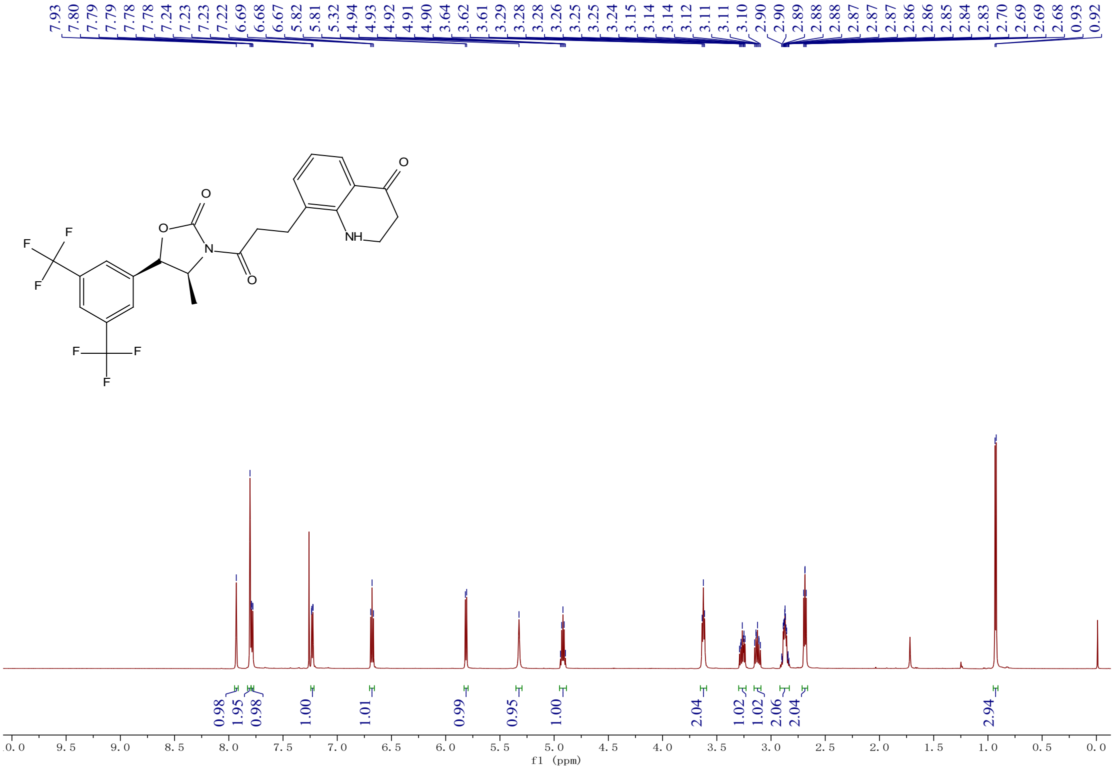


$^{13}$C NMR of Compound **15**

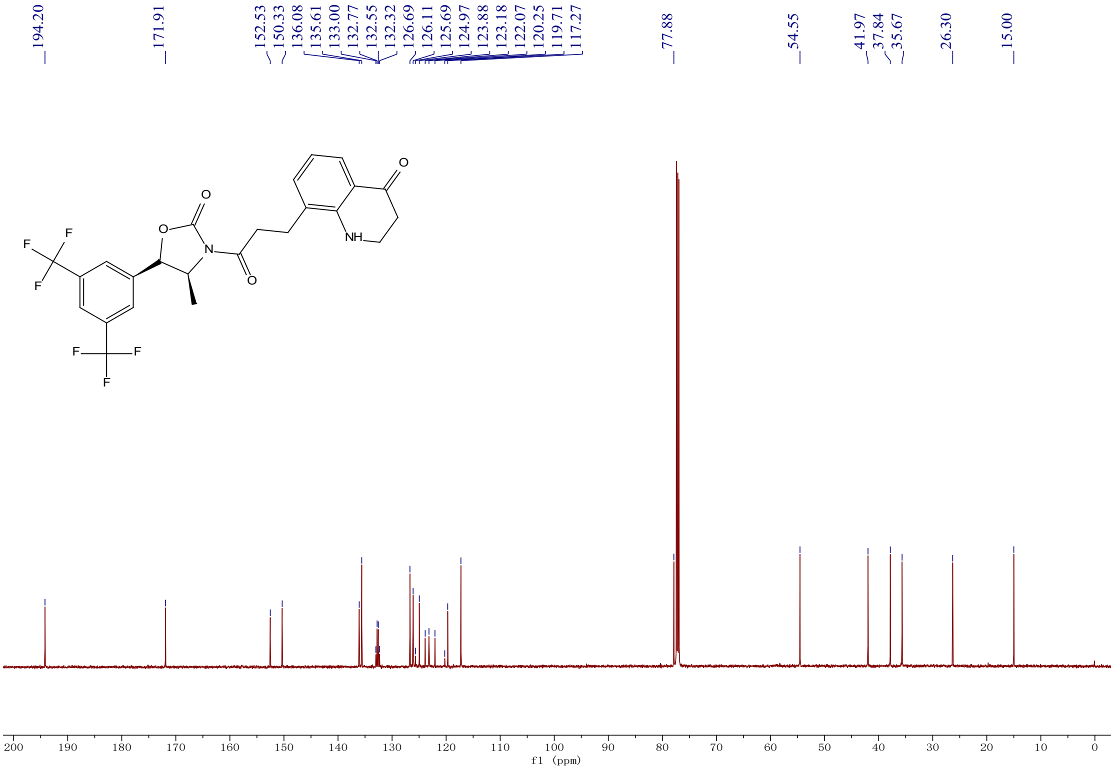

## $^{1}$H NMR of Compound **16**

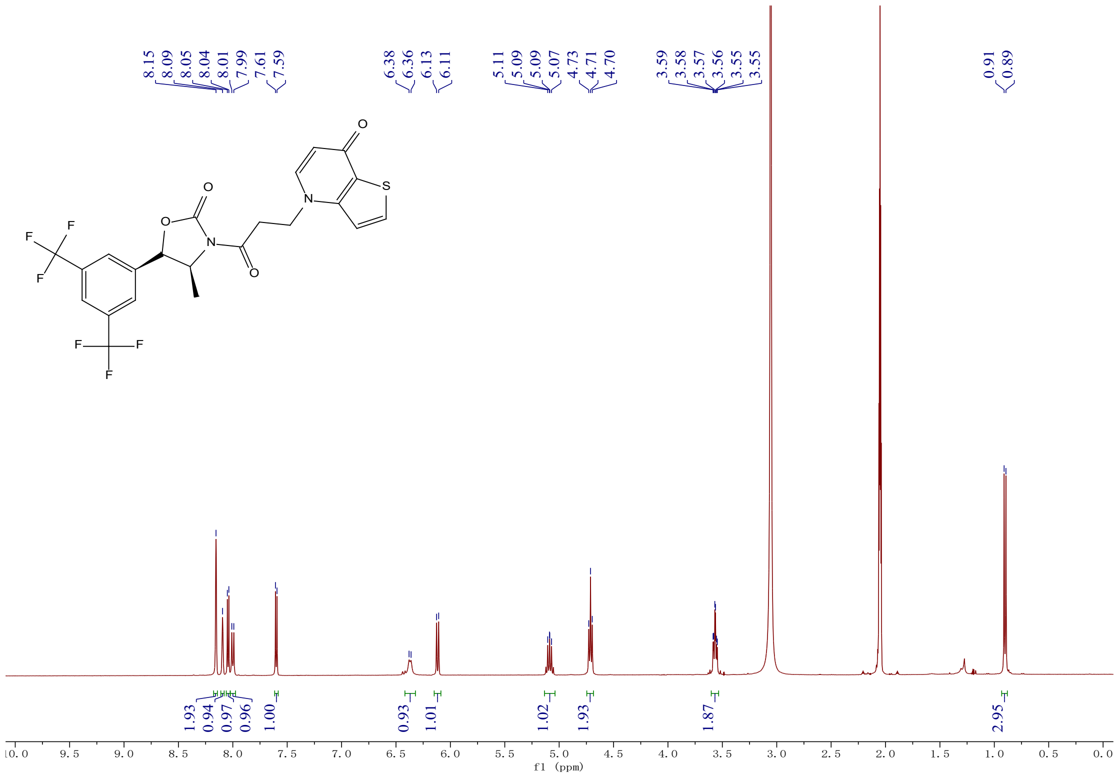

## $^{13}$C NMR of Compound **16**

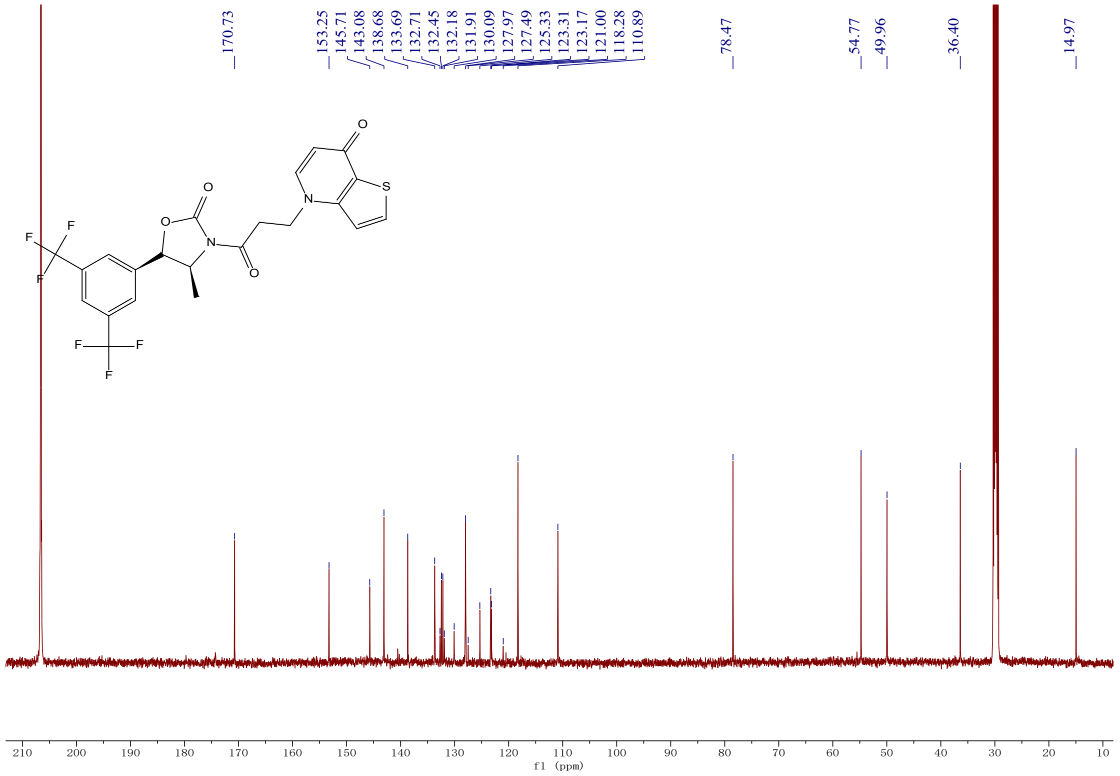

$^{1}$H NMR of Compound **17a**

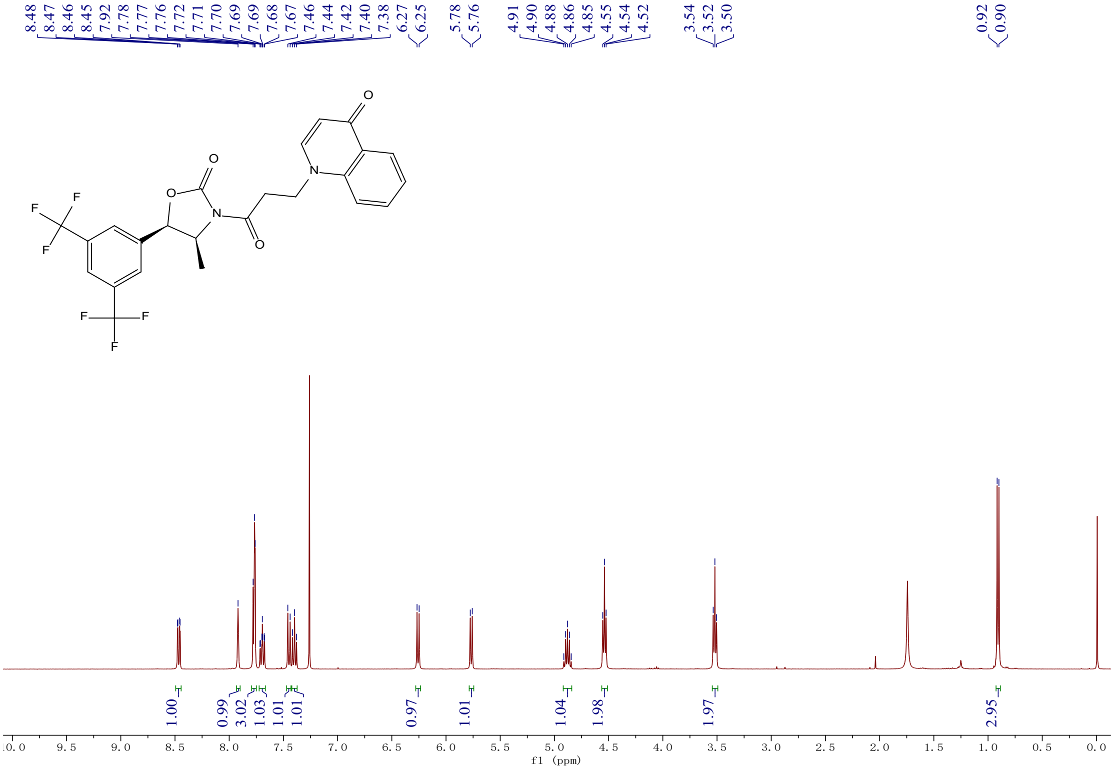

$^{13}$C NMR of Compound **17a**

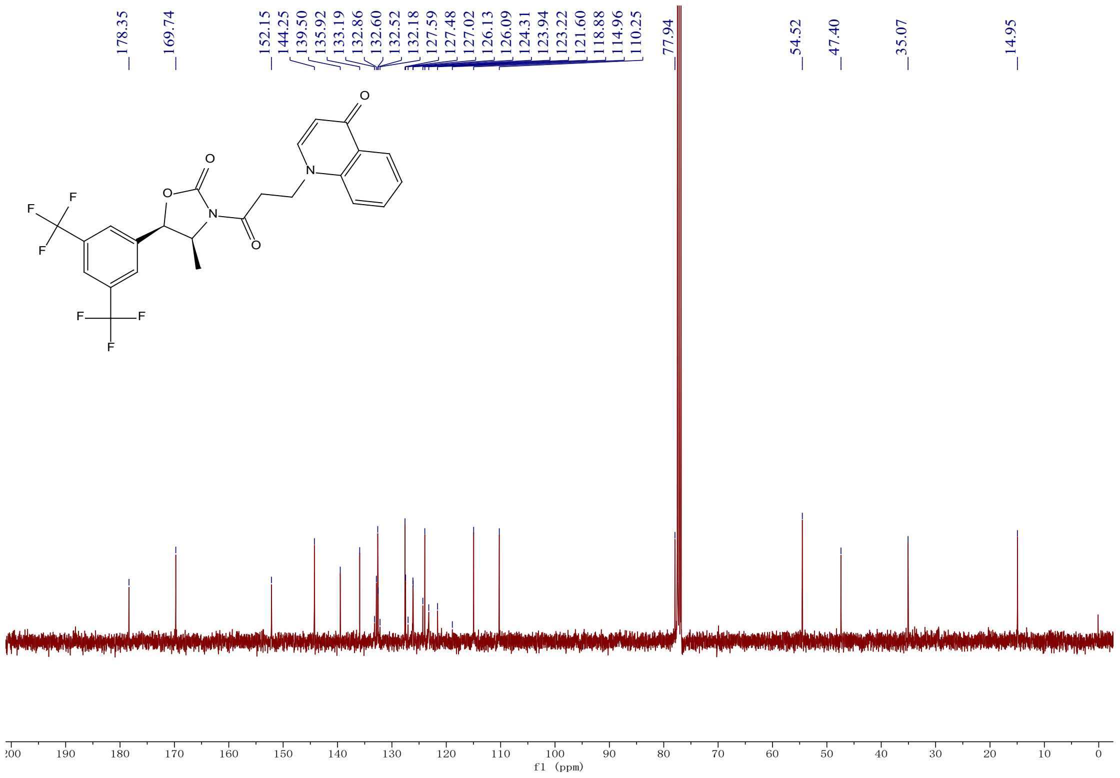


$^{1}$H NMR of Compound **17b**

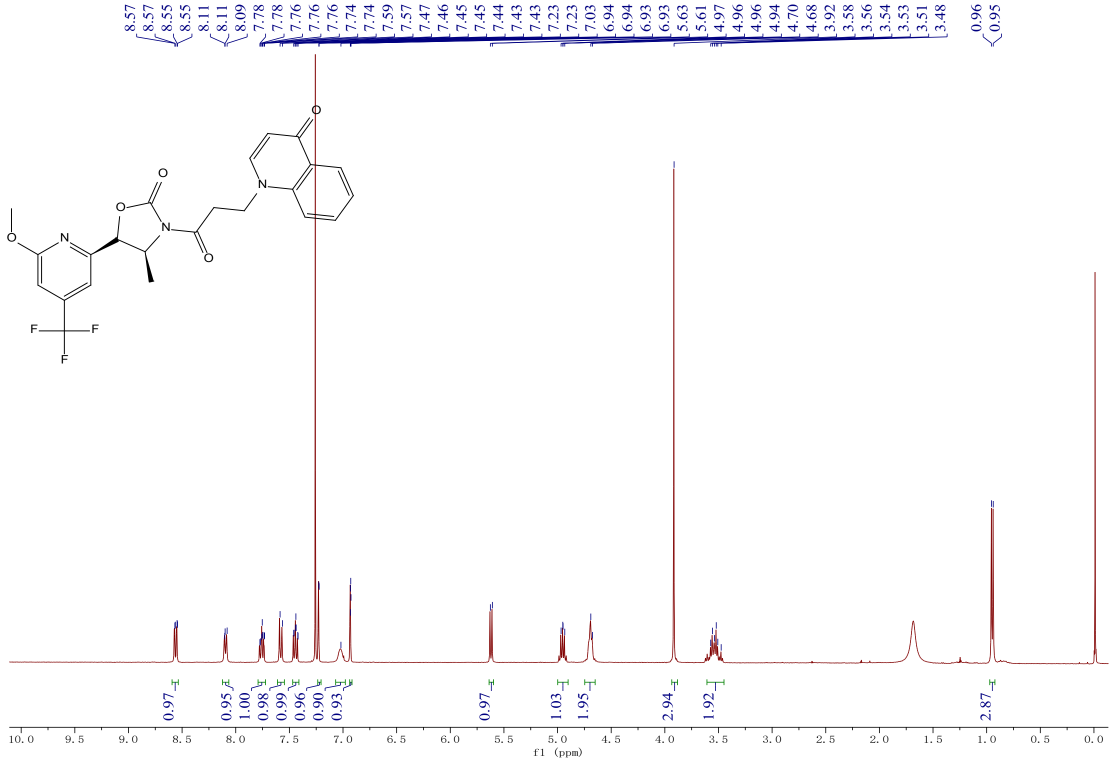

$^{13}$C NMR of Compound **17b**

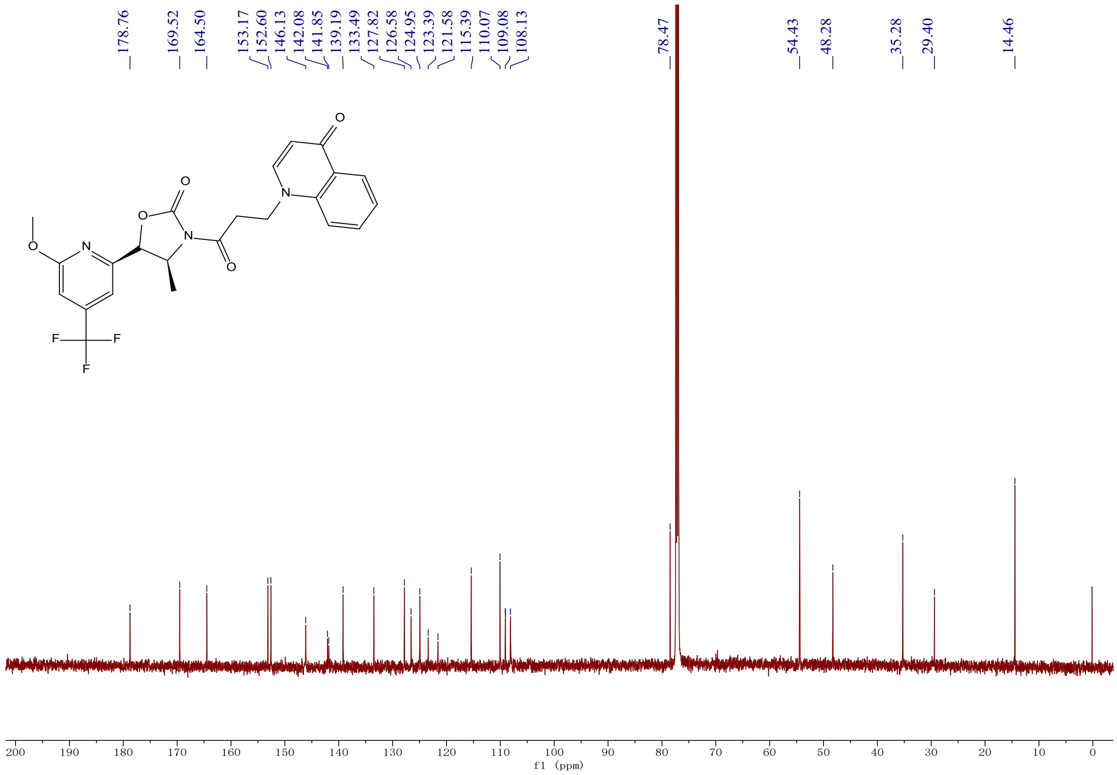


$^{1}$H NMR of Compound **17c**

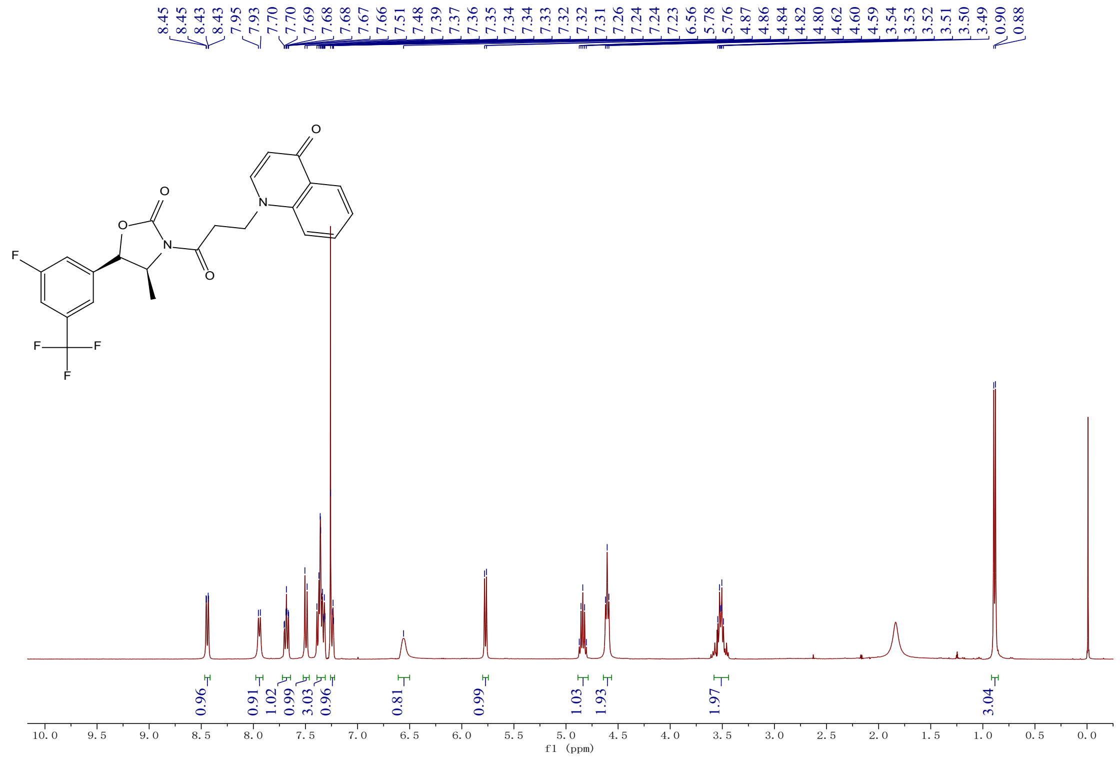


$^{13}$C NMR of Compound **17c**

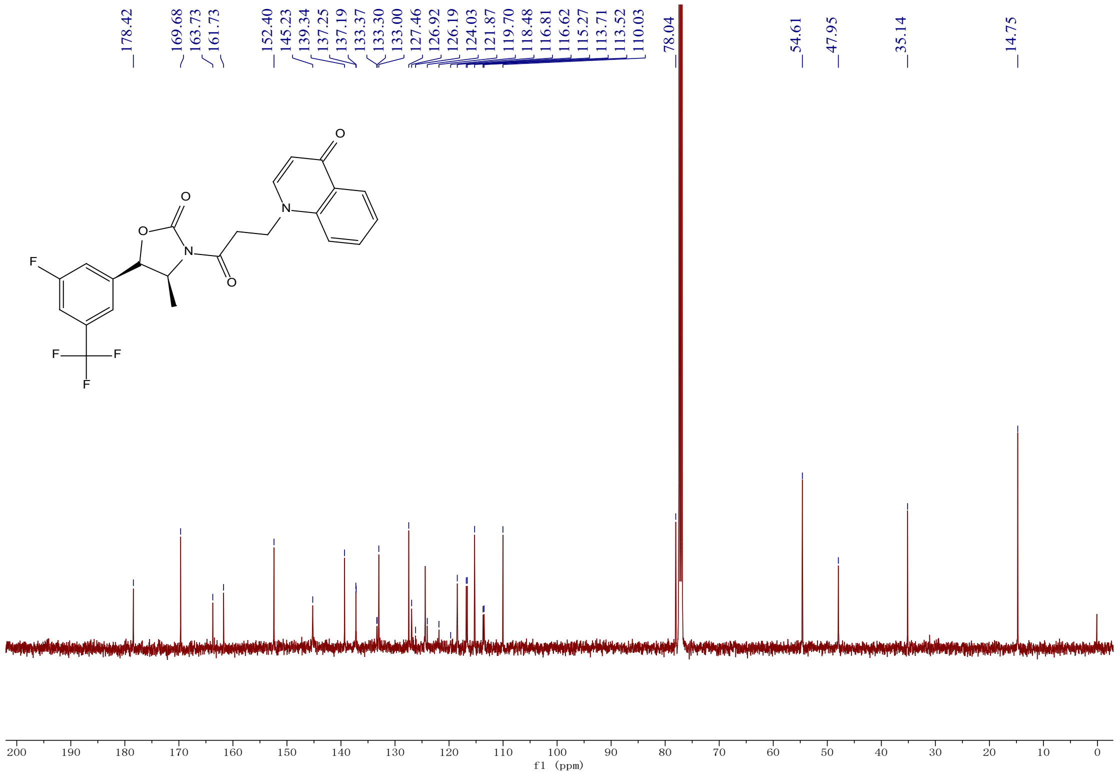


$^{1}$H NMR of Compound **17d**

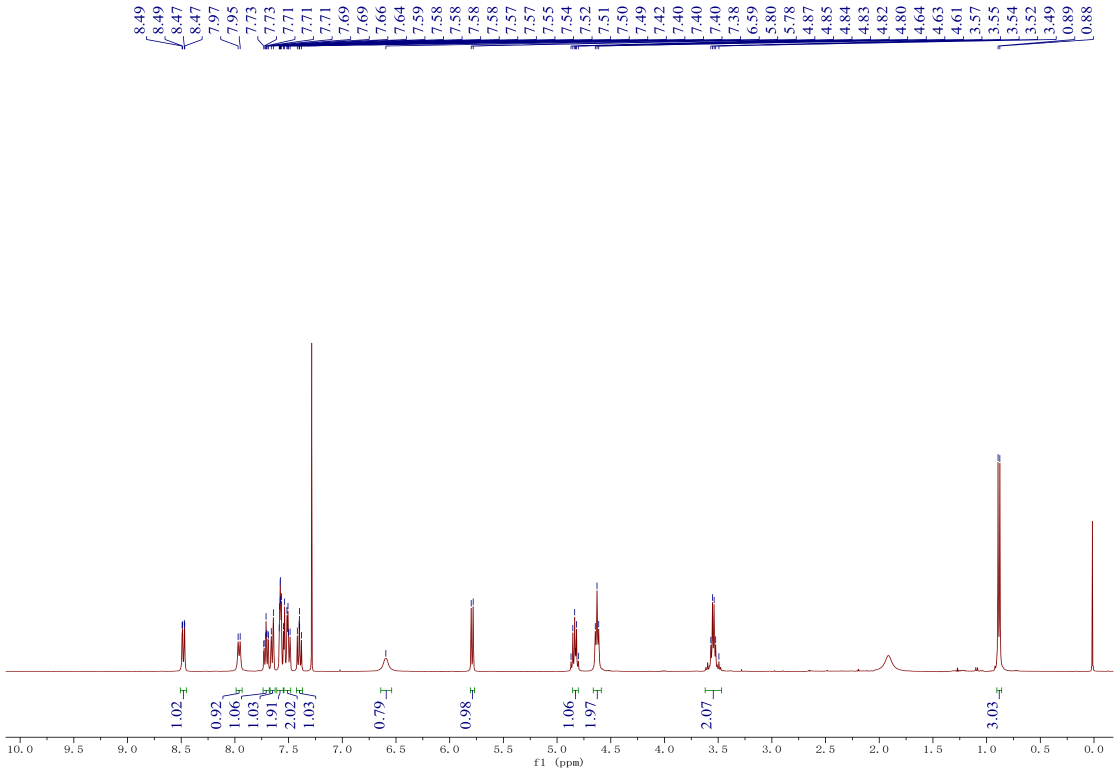


$^{13}$C NMR of Compound **17d**

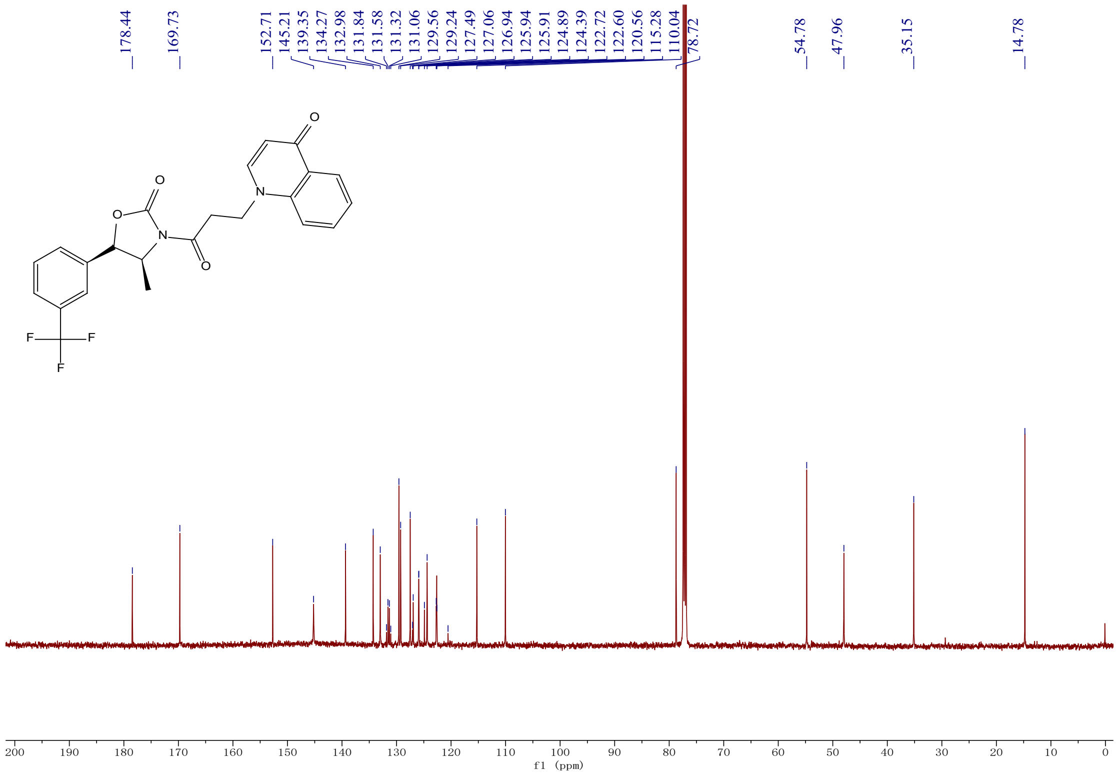

$^{1}$H NMR of Compound **18a**

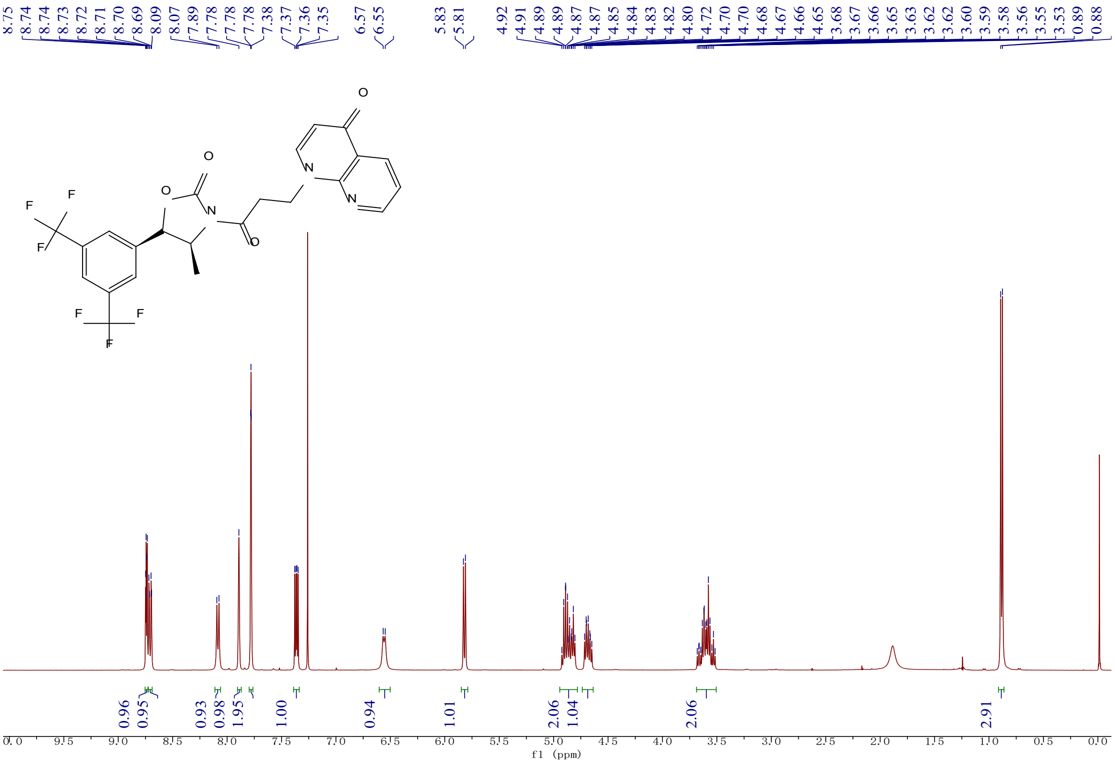


$^{13}$C NMR of Compound **18a**

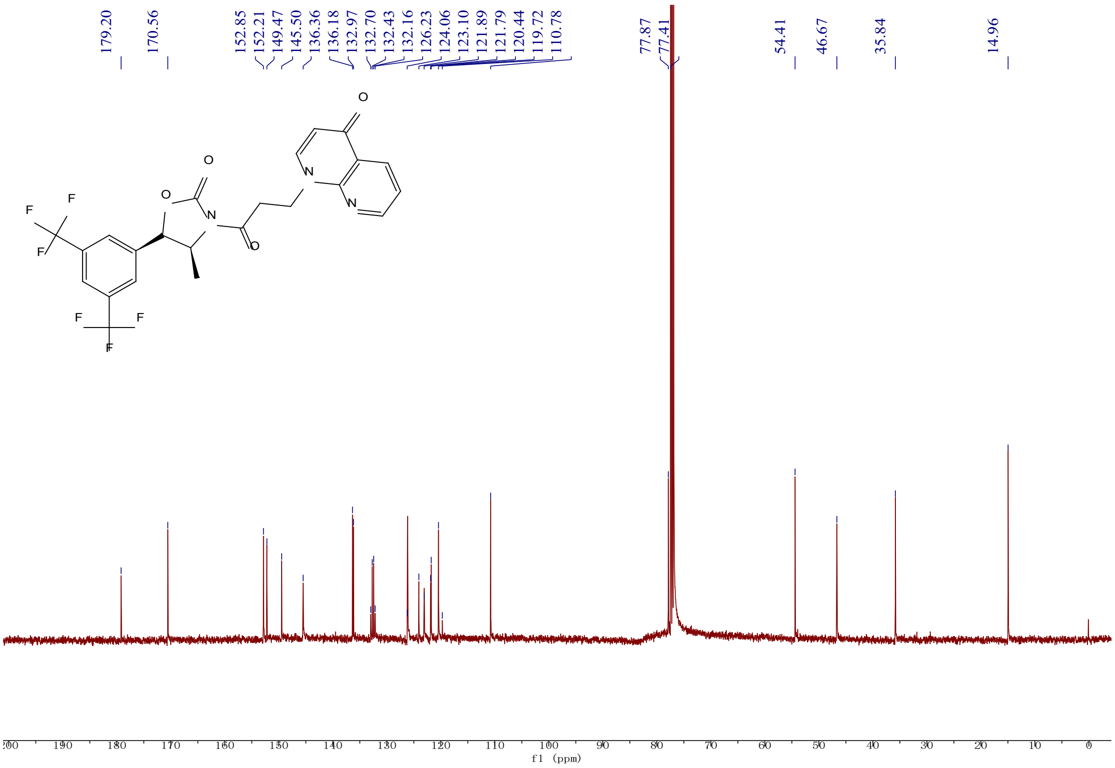

$^{1}$H NMR of Compound **18b**

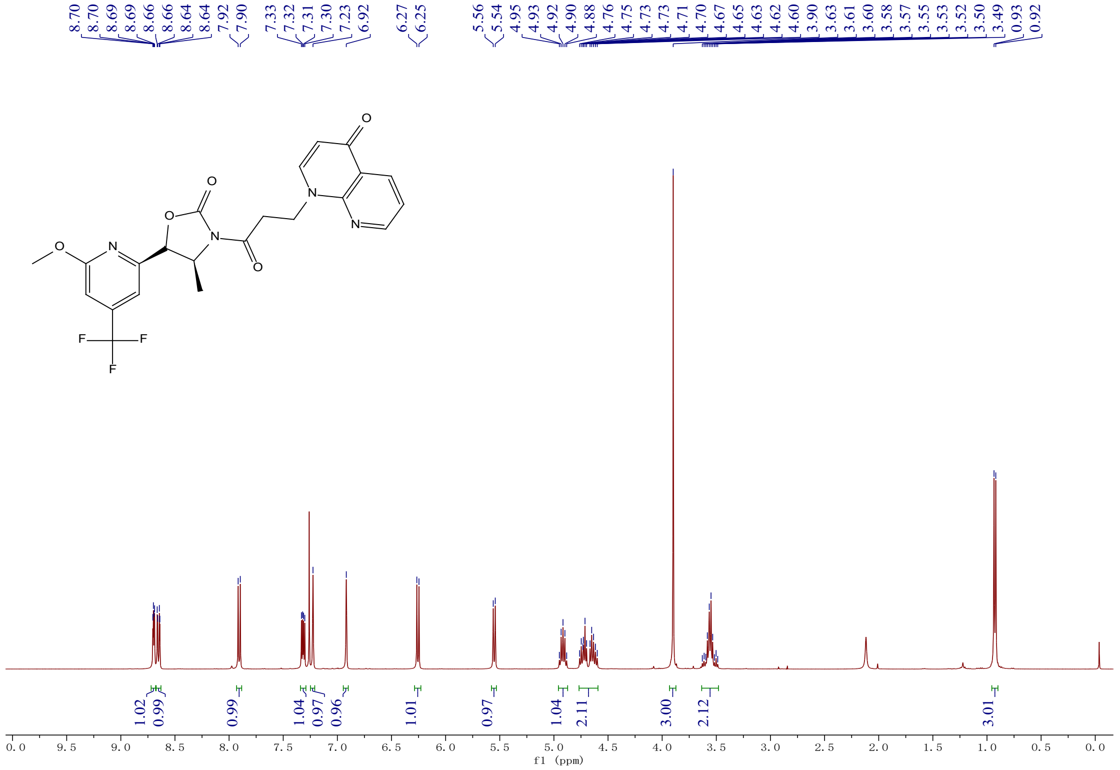

$^{13}$C NMR of Compound **18b**

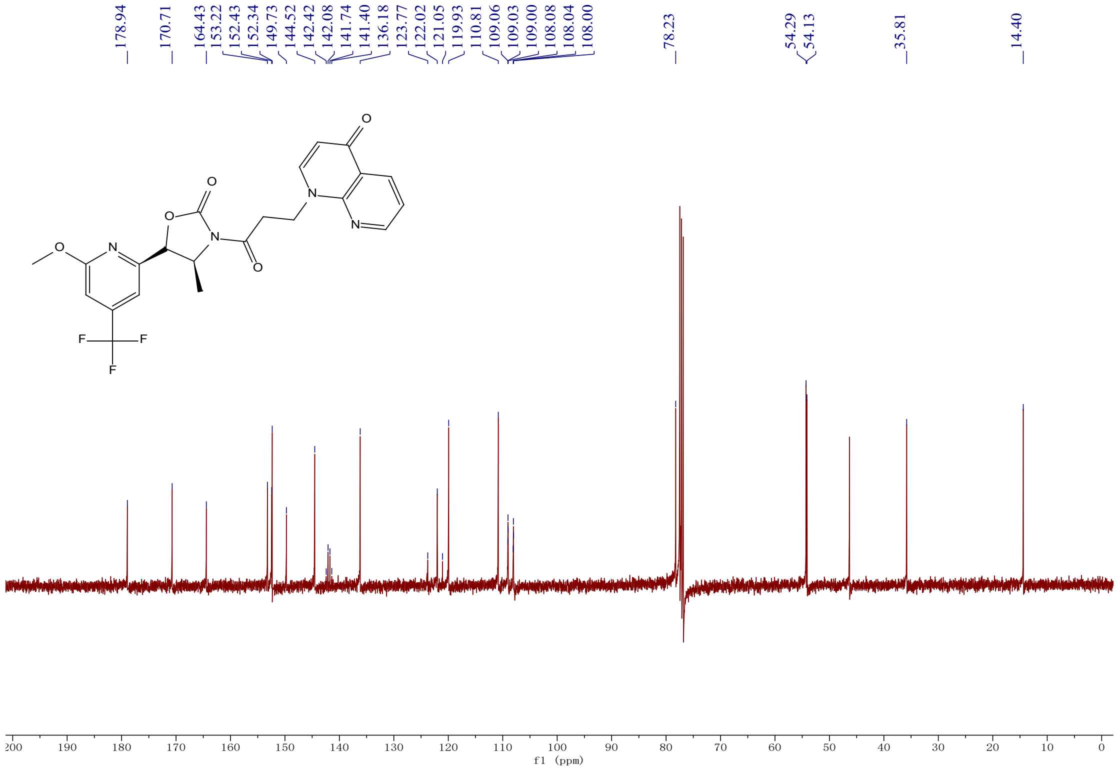

$^{1}$H NMR of Compound **19**

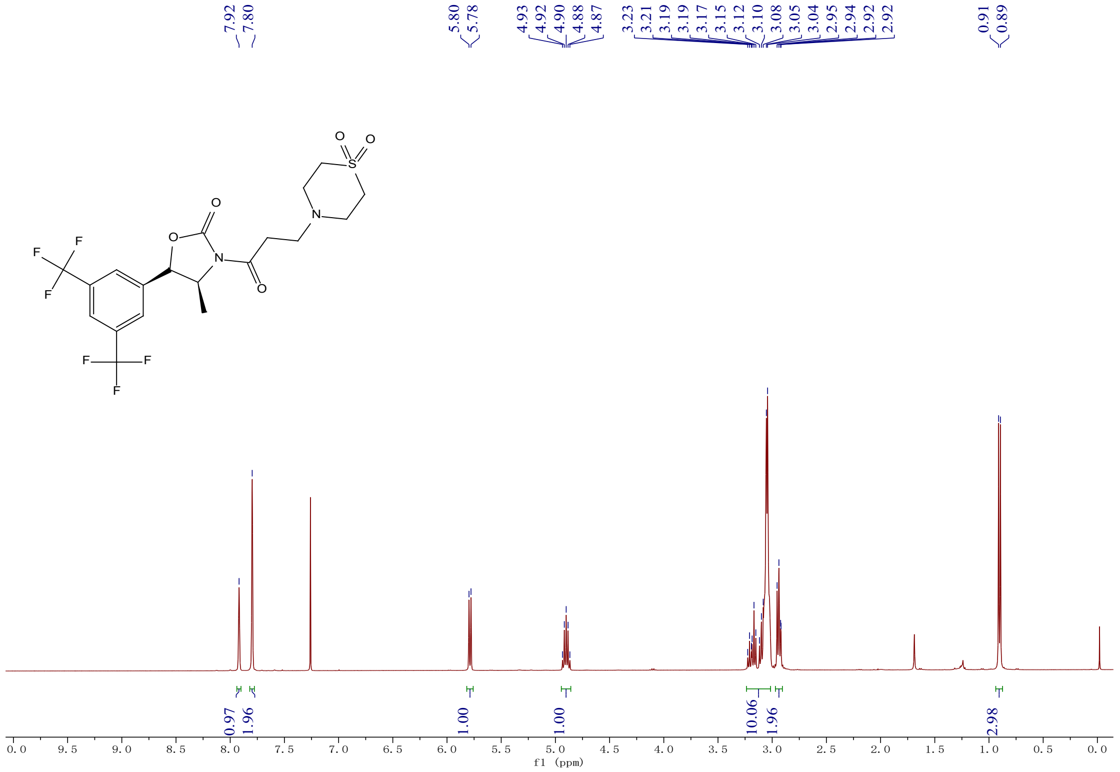


$^{13}$C NMR of Compound **19**

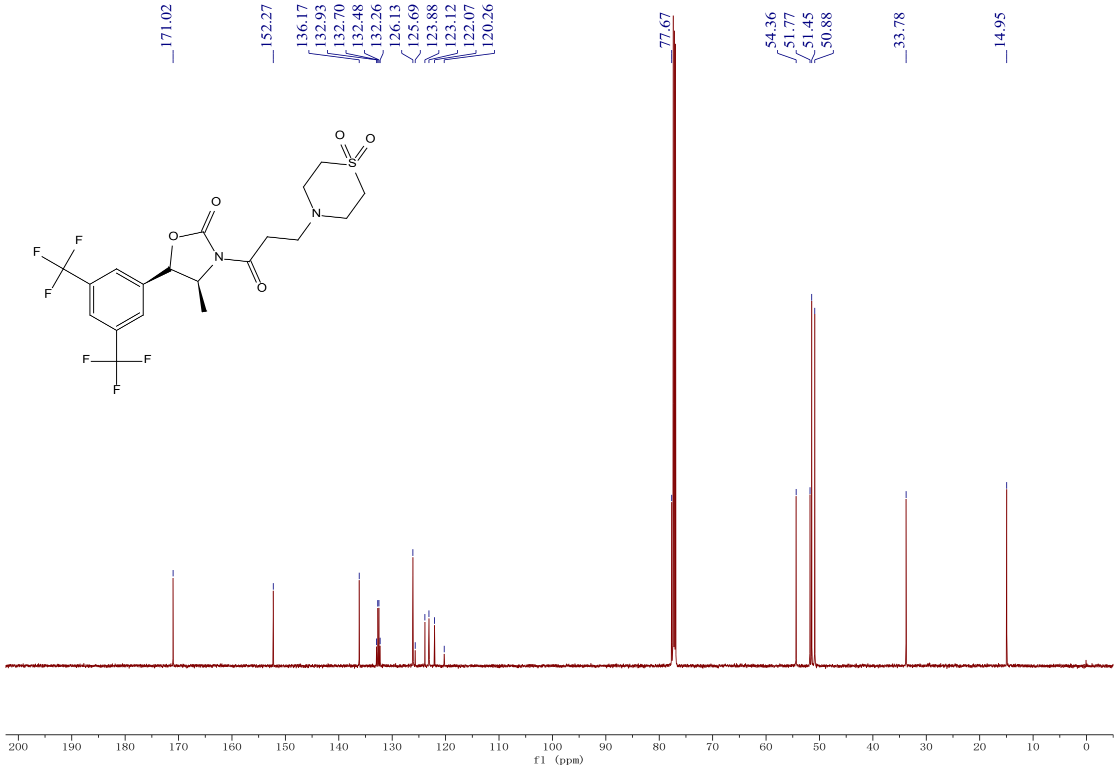

$^{1}$H NMR of Compound **20**

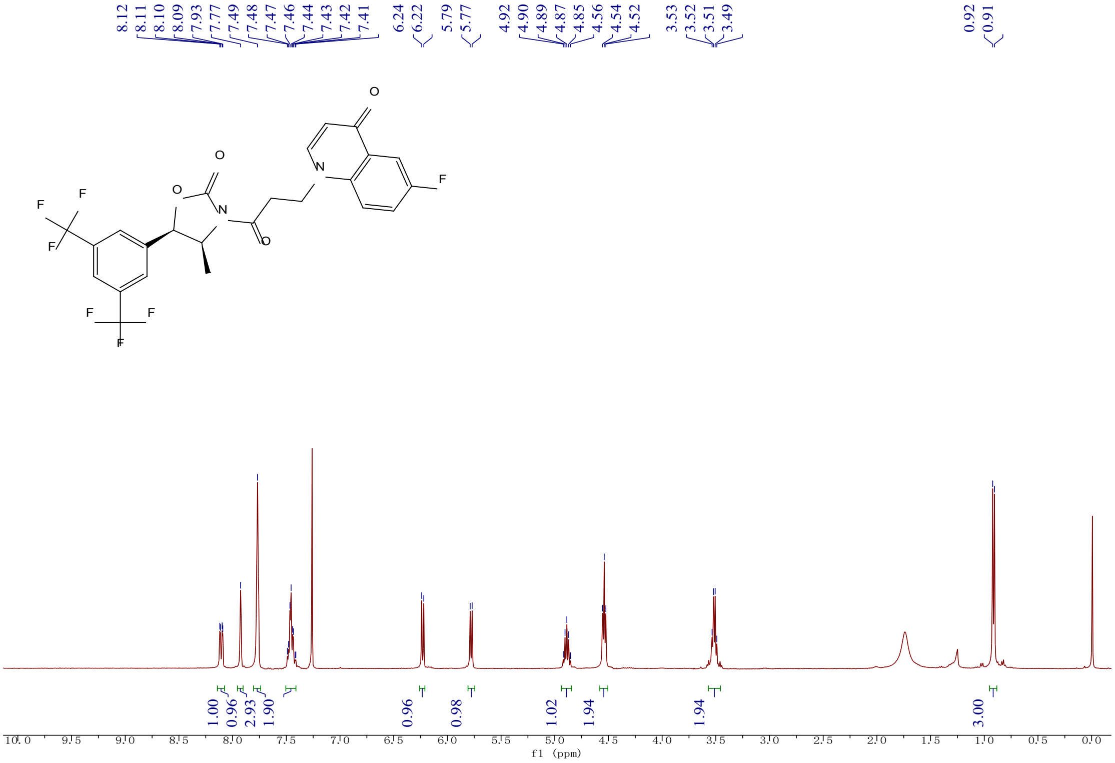


$^{13}$C NMR of Compound **20**

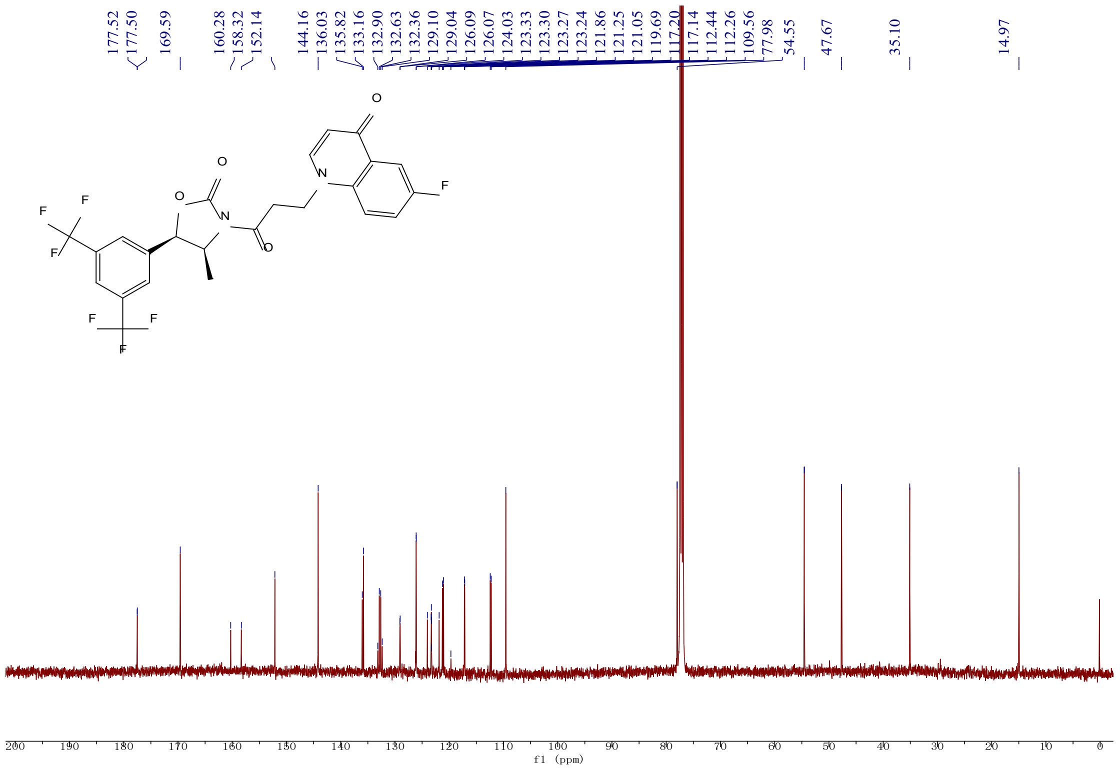


$^1$H NMR of Compound **21**

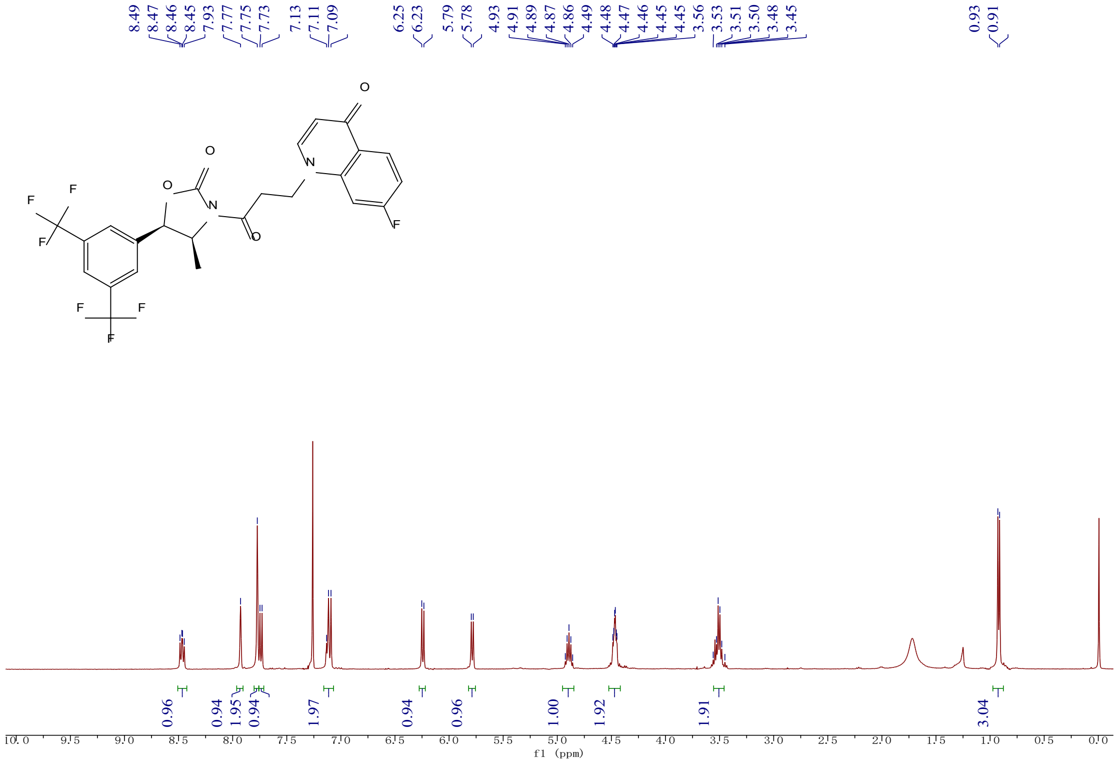

$^{13}$C NMR of Compound **21**

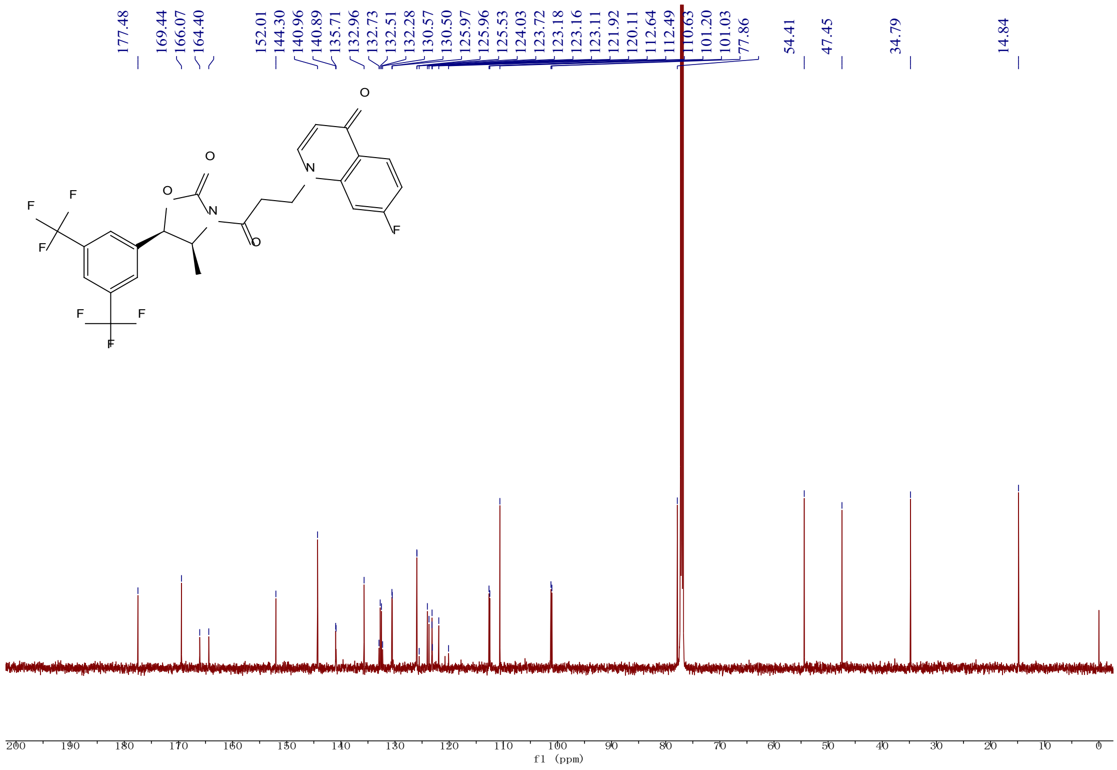

$^{1}$H NMR of Compound **22**

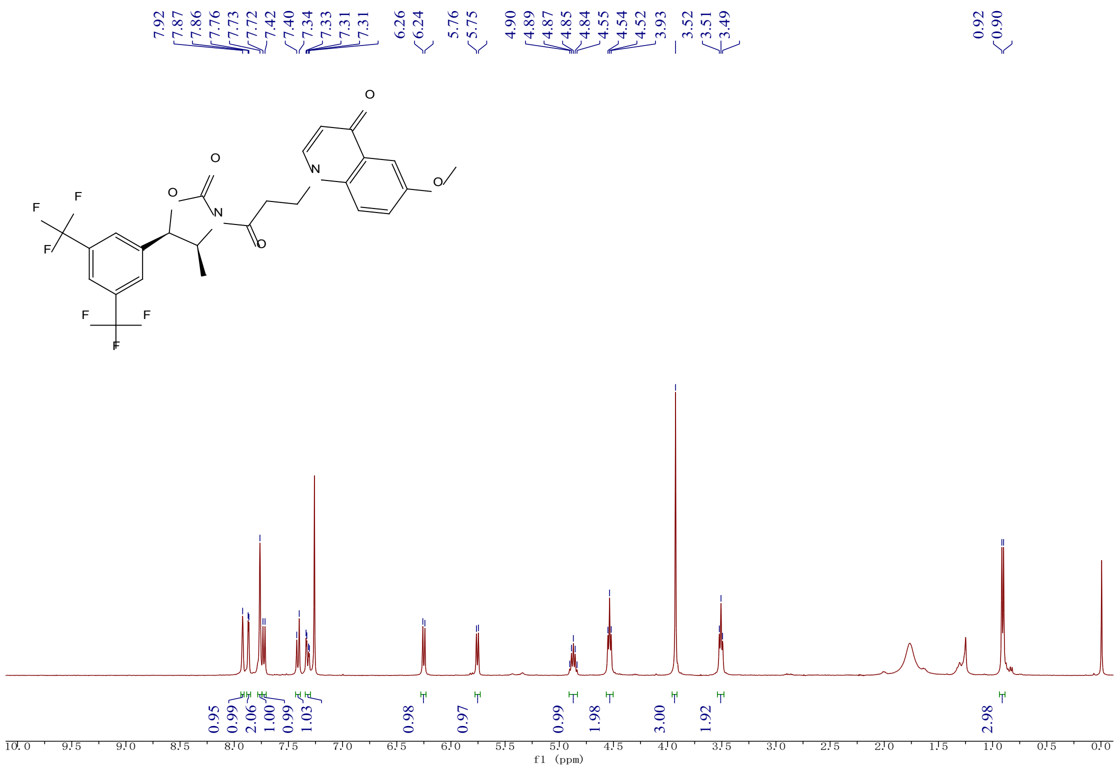

<sup></sup>

## $^{13}$C NMR of Compound **22**

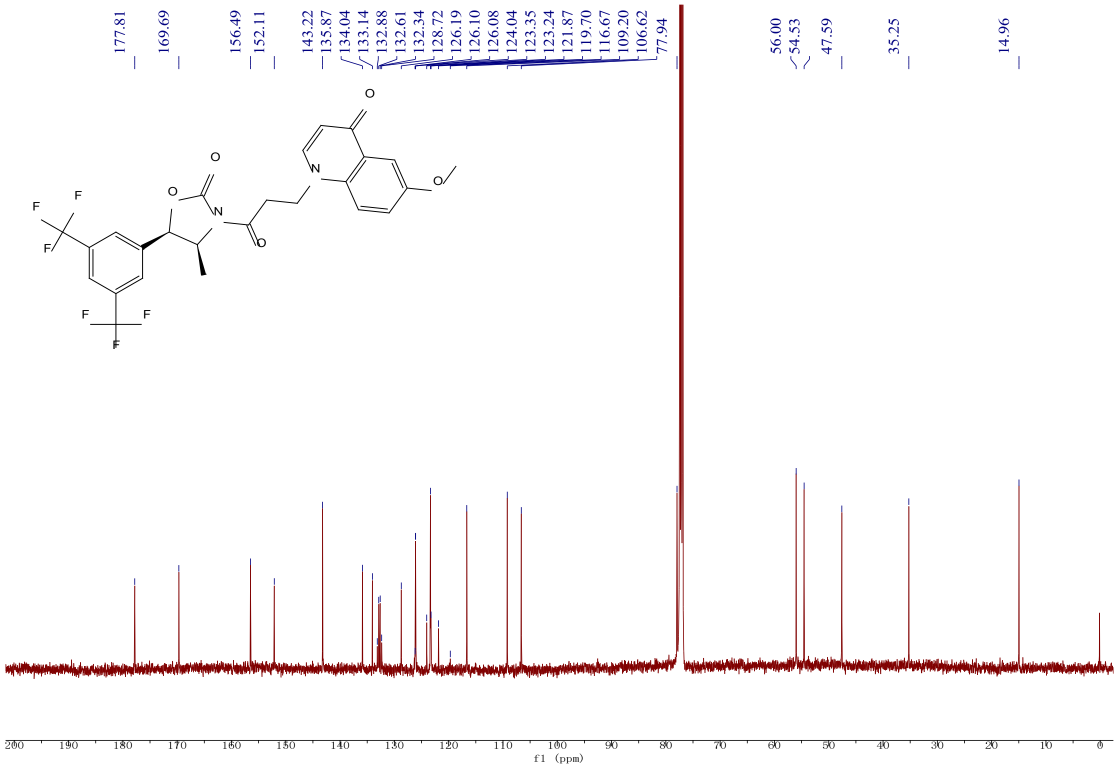

## $^{1}$H NMR of Compound **23**

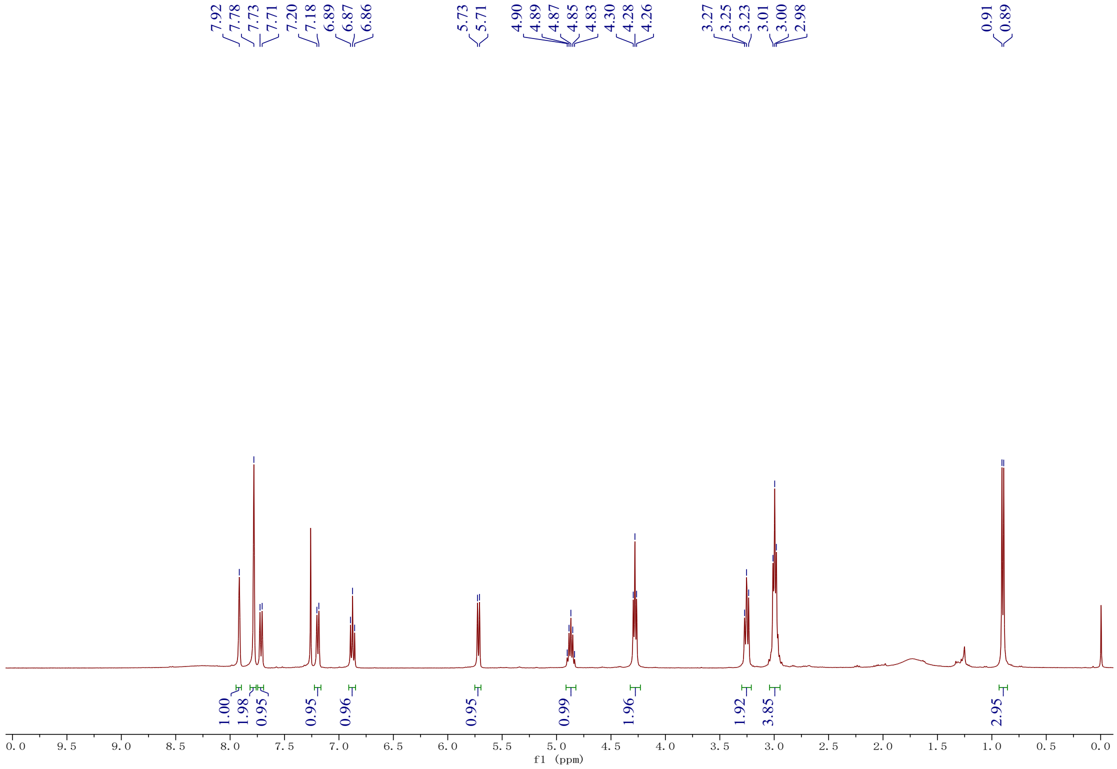


## $^{13}$C NMR of Compound **23**

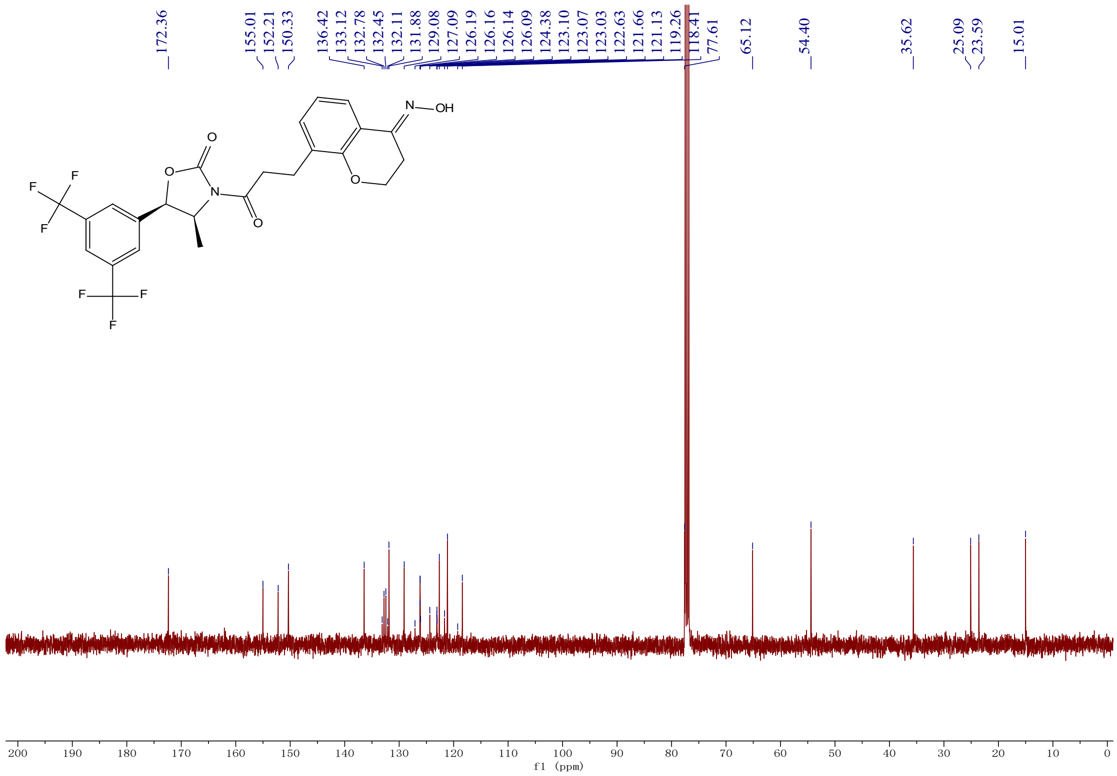

# $^{1}$H NMR of Compound **24**

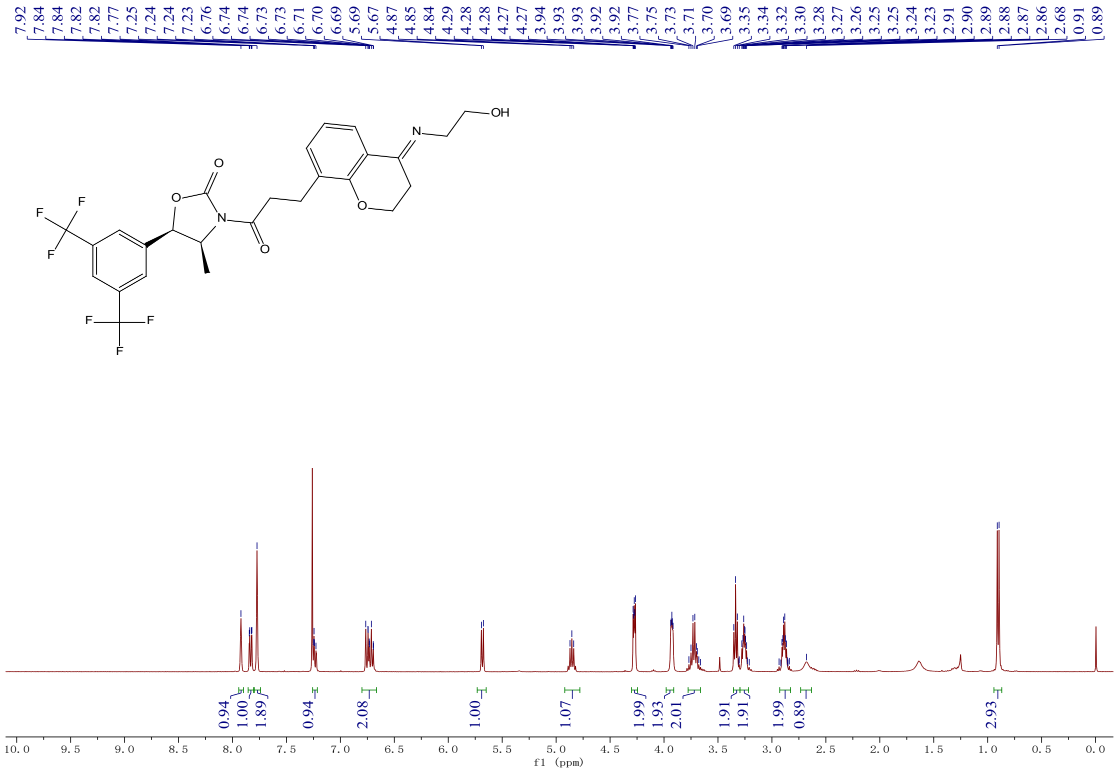


# $^{13}$C NMR of Compound 24

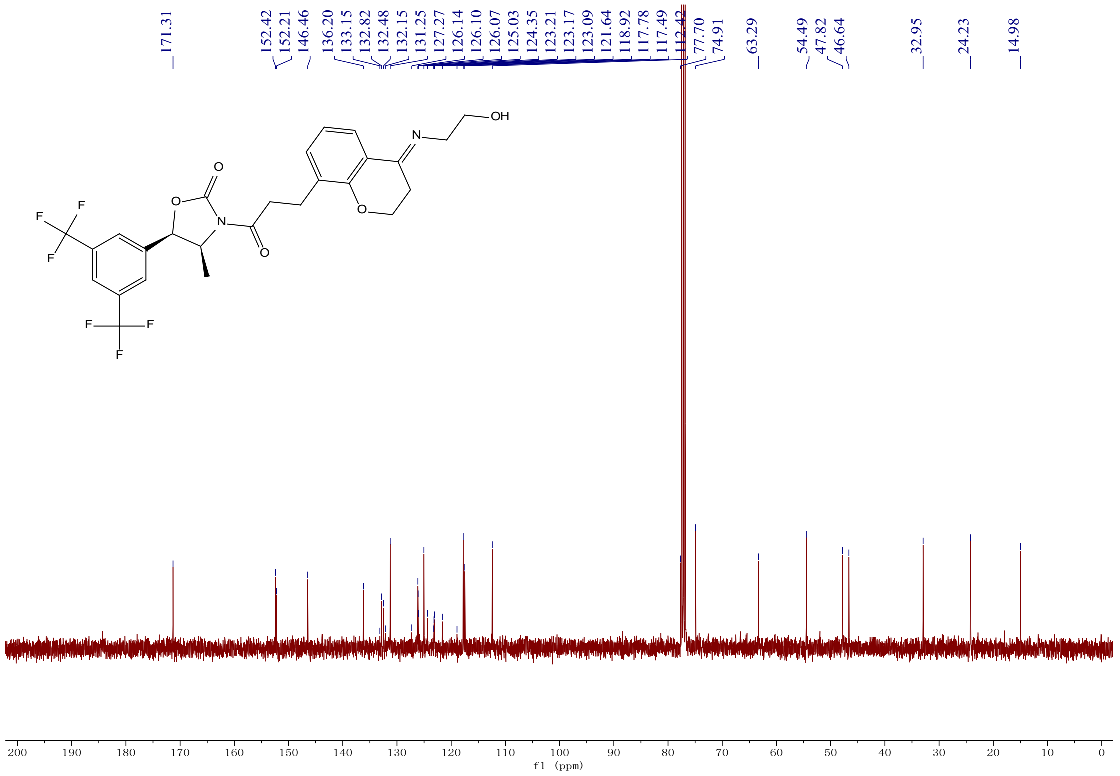

## $^1H$ NMR of Compound **25**

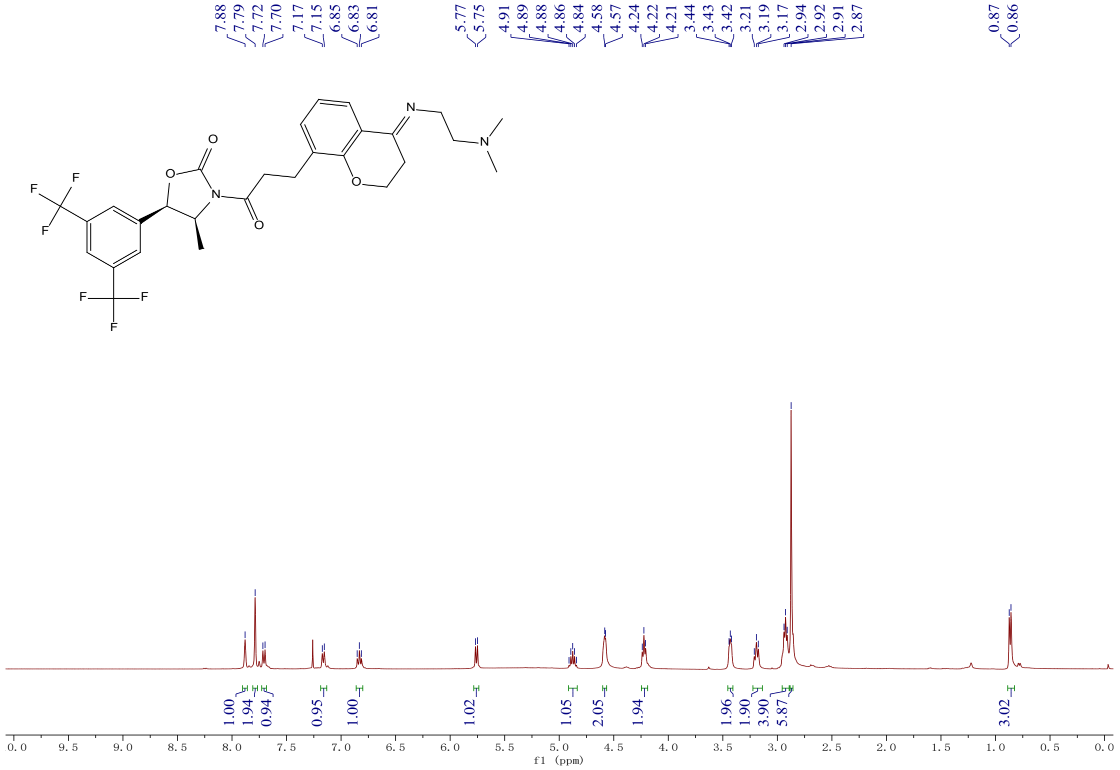


## $^{13}C$ NMR of Compound **25**

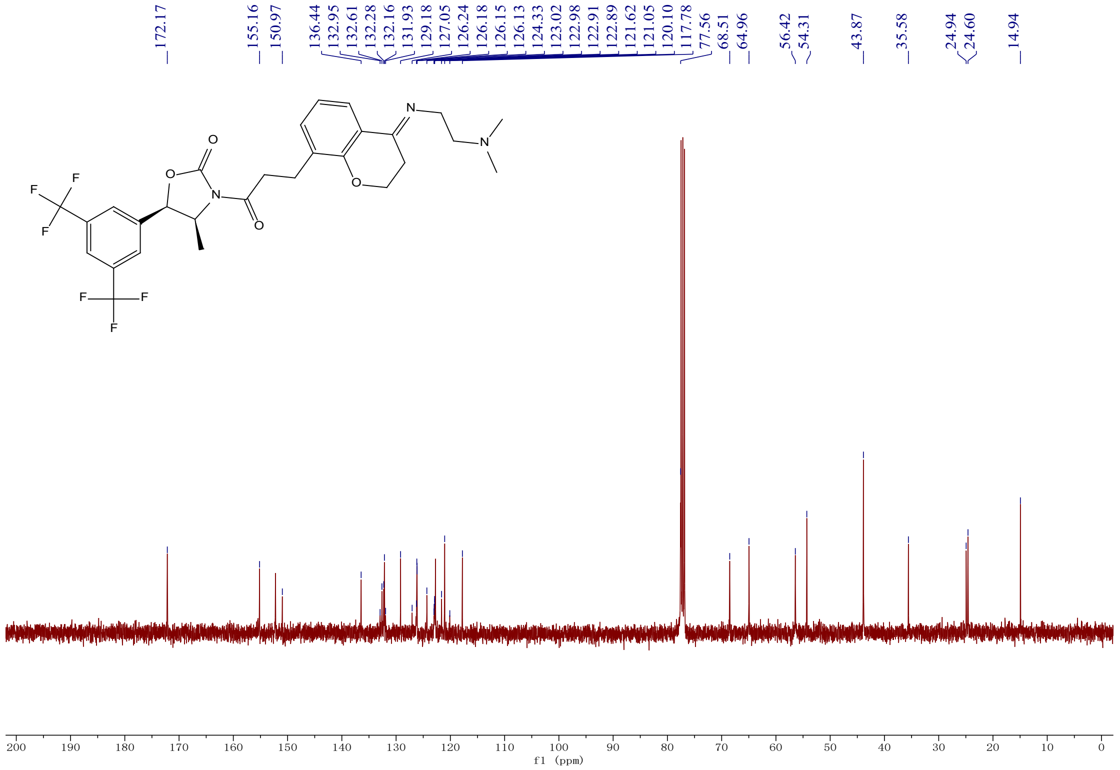

$^{1}$H NMR of Compound **26**

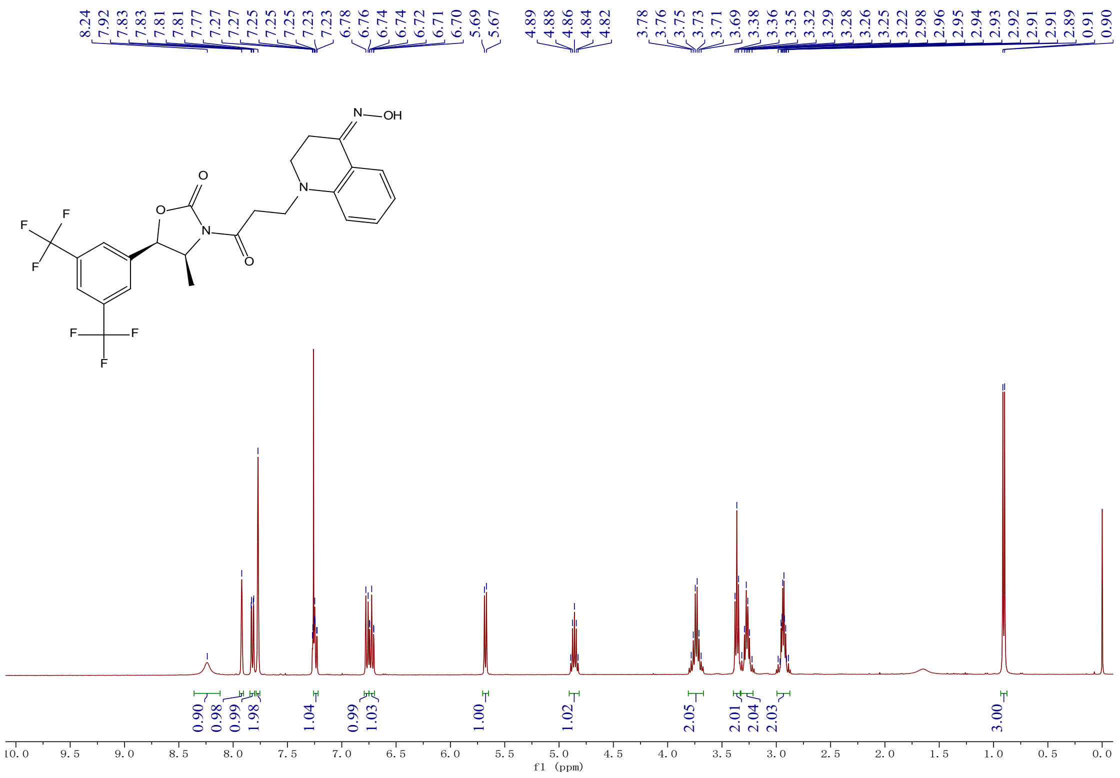

$^{13}$C NMR of Compound **26**

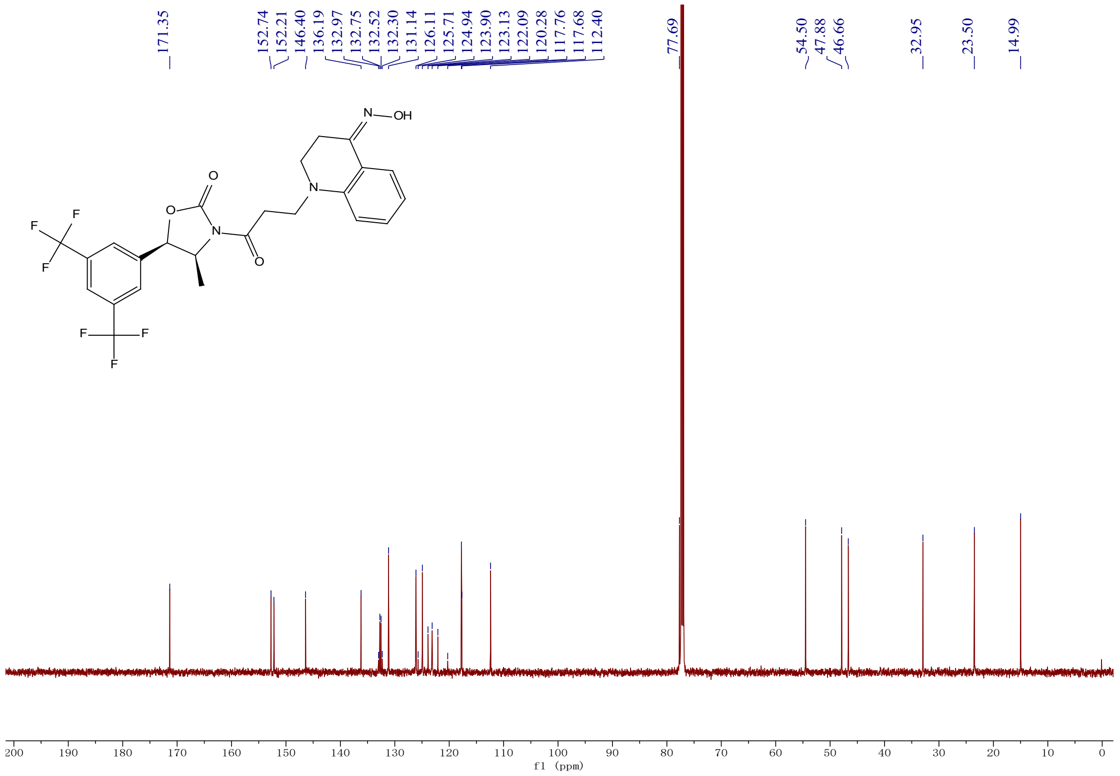

$^{1}$H NMR of Compound **27**

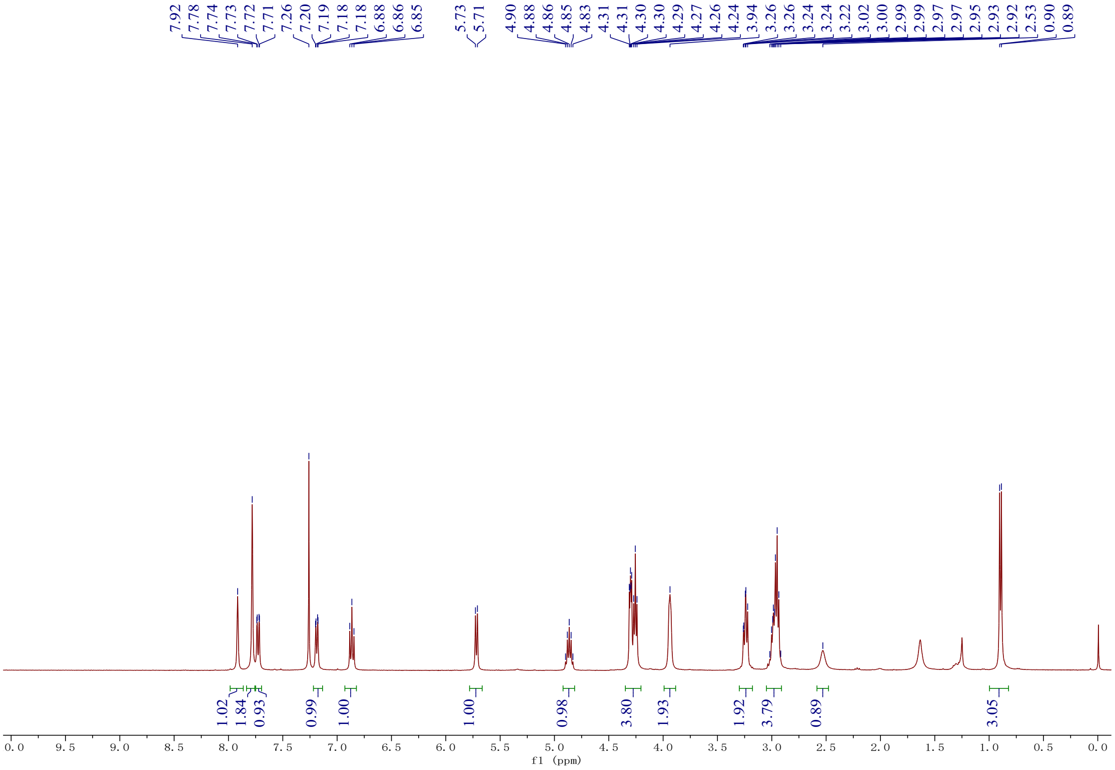

$^{13}$C NMR of Compound **27**

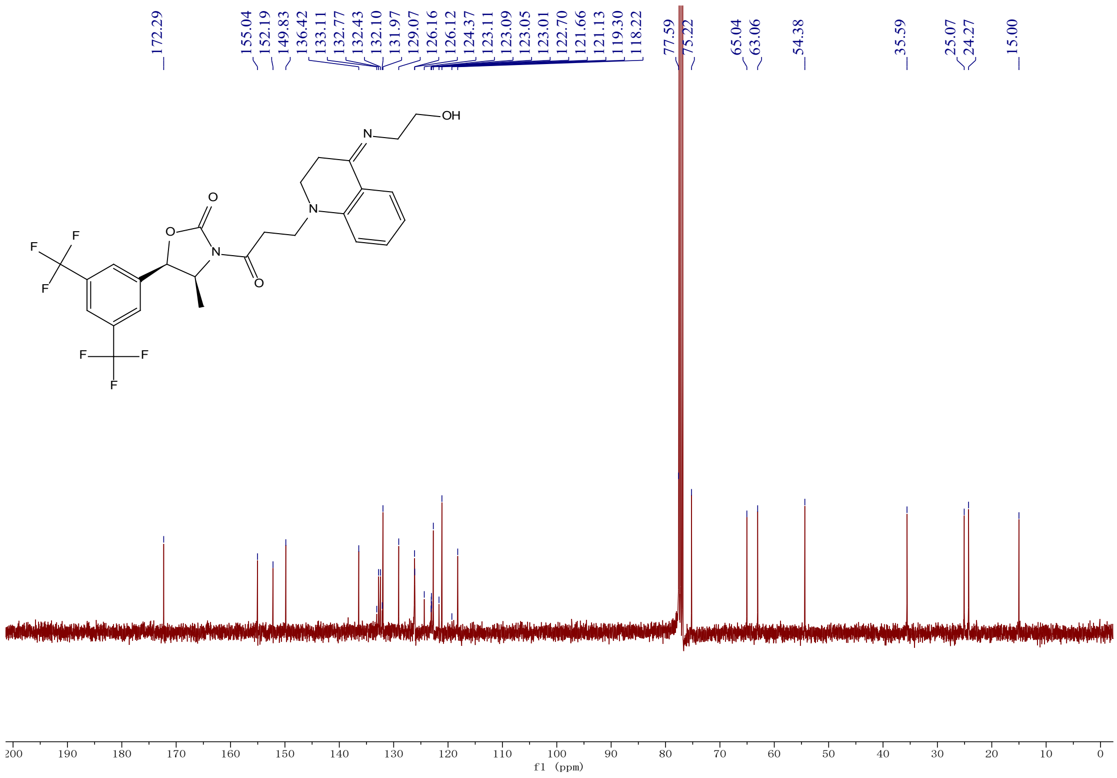
172.29
155.04
152.19
149.83
136.42
133.11
132.77
132.43
132.10
131.97
129.07
126.16
126.12
124.37
123.11
123.09
123.05
123.01
122.70
121.66
121.13
119.30
118.22
77.59
75.22
65.04
63.06
54.38
35.59
25.07
24.27
15.00
N
OH
O
O
N
O
N
F
F
F
F
F
F
200 190 180 170 160 150 140 130 120 110 100 90 80 70 60 50 40 30 20 10 0
f1 (ppm)

## 8. The purity of compounds

The purity of 1 (98.41%).

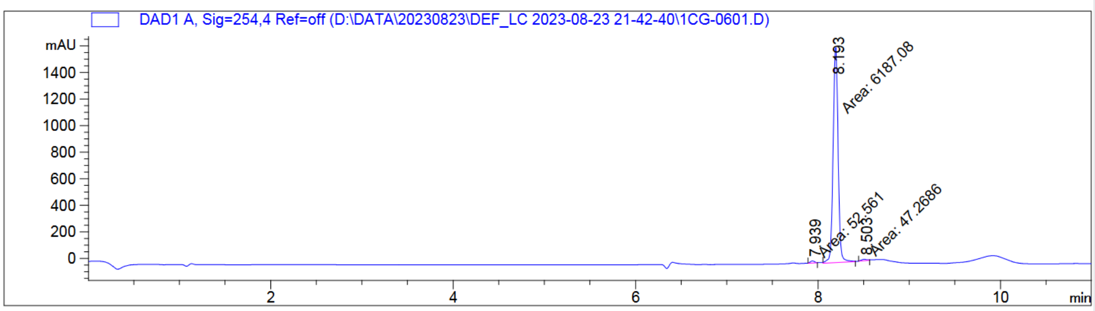


```
=====================================================================
                         Area Percent Report
=====================================================================

Sorted By             :      Signal
Multiplier:                  :       1.0000
Dilution:                    :       1.0000
Use Multiplier & Dilution Factor with ISTDs


Signal 1: DAD1 A, Sig=254,4 Ref=off

Peak RetTime Type  Width     Area       Height      Area
  #   [min]        [min]   [mAU*s]      [mAU]        %
----|-------|----|-------|----------|----------|--------|
   1   7.939 MM    0.0536   52.56097   16.33949   0.8360
   2   8.193 MM    0.0630 6187.07861 1636.32446  98.4121
   3   8.503 MM    0.0710   47.26858   11.09291   0.7519
```

The purity of 2 (96.29%).

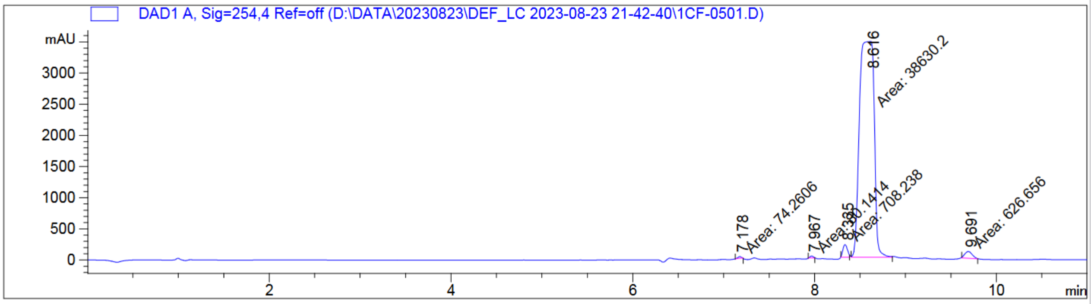


```
=====================================================================
                         Area Percent Report
=====================================================================

Sorted By             :      Signal
Multiplier:                  :      1.0000
Dilution:                    :      1.0000
Use Multiplier & Dilution Factor with ISTDs


Signal 1: DAD1 A, Sig=254,4 Ref=off
```

| Peak # | RetTime [min] | Type | Width [min] | Area [mAU*s] | Height [mAU] | Area % |
|---|---|---|---|---|---|---|
| 1 | 7.178 | MM | 0.0440 | 74.26057 | 28.14883 | 0.1851 |
| 2 | 7.967 | MM | 0.0441 | 80.14139 | 30.26535 | 0.1998 |
| 3 | 8.335 | MM | 0.0578 | 708.23767 | 204.14017 | 1.7653 |
| 4 | 8.616 | MM | 0.1860 | 3.86302e4 | 3460.97729 | 96.2878 |
| 5 | 9.691 | MM | 0.0951 | 626.65637 | 109.85093 | 1.5620 |

The purity of 3 (95.57%).

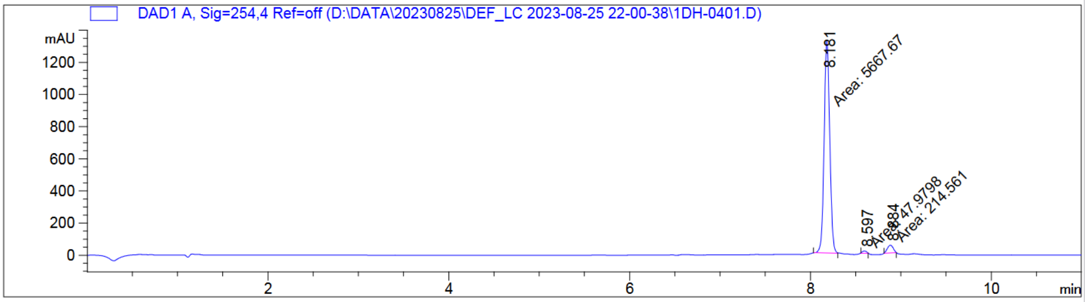


```
=====================================================================
                         Area Percent Report
=====================================================================

Sorted By             :      Signal
Multiplier:                  :      1.0000
Dilution:                    :      1.0000
Use Multiplier & Dilution Factor with ISTDs


Signal 1: DAD1 A, Sig=254,4 Ref=off
```

| Peak # | RetTime [min] | Type | Width [min] | Area [mAU*s] | Height [mAU] | Area % |
|---|---|---|---|---|---|---|
| 1 | 8.181 | MM | 0.0714 | 5667.66895 | 1322.63232 | 95.5728 |
| 2 | 8.597 | MM | 0.0523 | 47.97982 | 15.27612 | 0.8091 |
| 3 | 8.884 | MM | 0.0734 | 214.56114 | 48.72253 | 3.6181 |

The purity of 4 (96.74%).

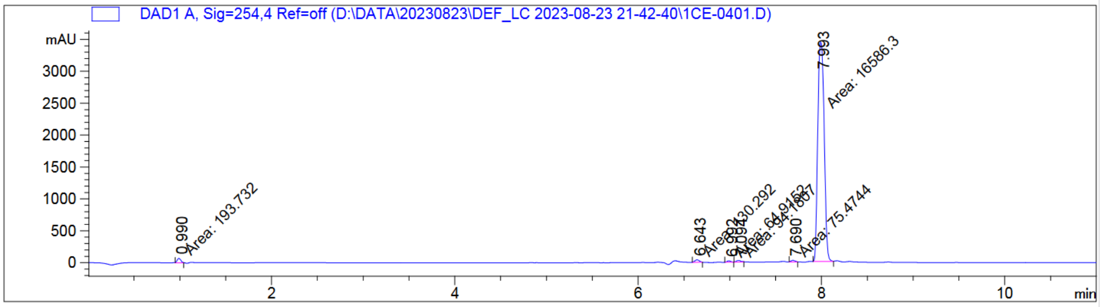


```
=====================================================================
                         Area Percent Report
=====================================================================

Sorted By             :      Signal
Multiplier:                   :      1.0000
Dilution:                     :      1.0000
Use Multiplier & Dilution Factor with ISTDs


Signal 1: DAD1 A, Sig=254,4 Ref=off
```

| Peak # | RetTime [min] | Type | Width [min] | Area [mAU*s] | Height [mAU] | Area % |
|---|---|---|---|---|---|---|
| 1 | 0.990 | MM | 0.0464 | 193.73215 | 69.63570 | 1.1300 |
| 2 | 6.643 | MM | 0.0578 | 130.29202 | 37.59547 | 0.7599 |
| 3 | 6.992 | MM | 0.0509 | 64.91521 | 21.25394 | 0.3786 |
| 4 | 7.094 | MM | 0.0592 | 94.18075 | 26.52461 | 0.5493 |
| 5 | 7.690 | MM | 0.0500 | 75.47444 | 25.17246 | 0.4402 |
| 6 | 7.993 | MM | 0.0802 | 1.65863e4 | 3445.15771 | 96.7419 |

The purity of 5 (96.87%).

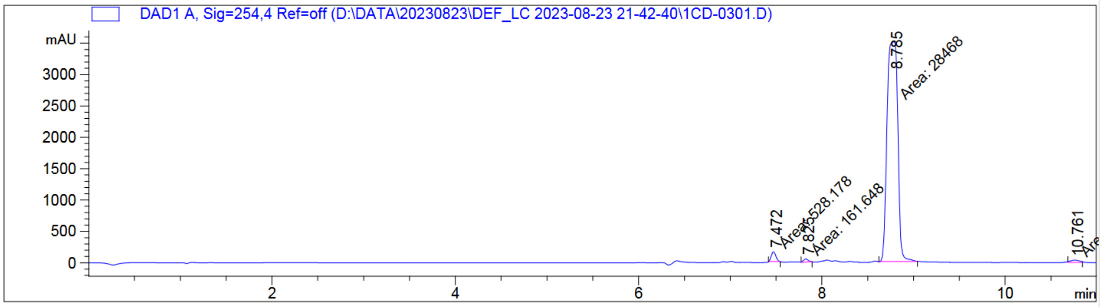


```
=====================================================================
                         Area Percent Report
=====================================================================

Sorted By             :      Signal
Multiplier:                   :      1.0000
Dilution:                     :      1.0000
Use Multiplier & Dilution Factor with ISTDs


Signal 1: DAD1 A, Sig=254,4 Ref=off
```

| Peak # | RetTime [min] | Type | Width [min] | Area [mAU*s] | Height [mAU] | Area % |
|---|---|---|---|---|---|---|
| 1 | 7.472 | MM | 0.0568 | 528.17773 | 154.88484 | 1.7973 |
| 2 | 7.825 | MM | 0.0569 | 161.64807 | 47.38398 | 0.5501 |
| 3 | 8.785 | MM | 0.1355 | 2.84680e4 | 3501.04297 | 96.8732 |
| 4 | 10.761 | MM | 0.1133 | 229.04153 | 33.67862 | 0.7794 |

The purity of 6 (98.02%).

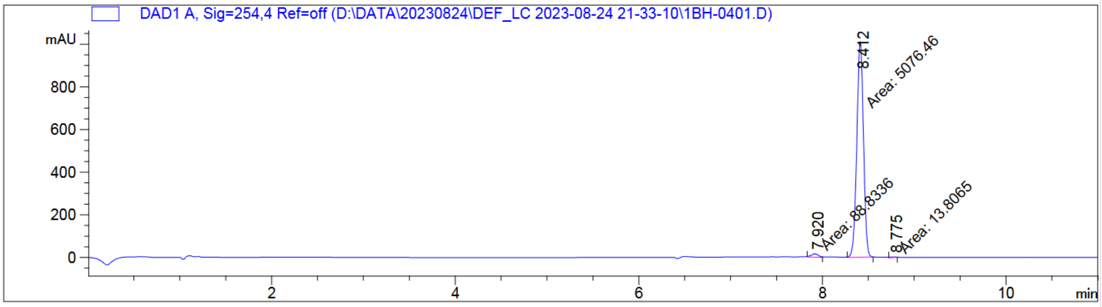


```
=====================================================================
                         Area Percent Report
=====================================================================

Sorted By             :      Signal
Multiplier:                   :      1.0000
Dilution:                     :      1.0000
Use Multiplier & Dilution Factor with ISTDs


Signal 1: DAD1 A, Sig=254,4 Ref=off

Peak RetTime Type  Width     Area      Height     Area
  #   [min]        [min]   [mAU*s]     [mAU]        %
----|-------|----|-------|----------|----------|--------|
   1   7.920 MM    0.0935   88.83358   15.82836   1.7152
   2   8.412 MM    0.0835 5076.45703 1012.81555  98.0182
   3   8.775 MM    0.0776   13.80655    2.96491   0.2666
```

The purity of 7 (98.32%).

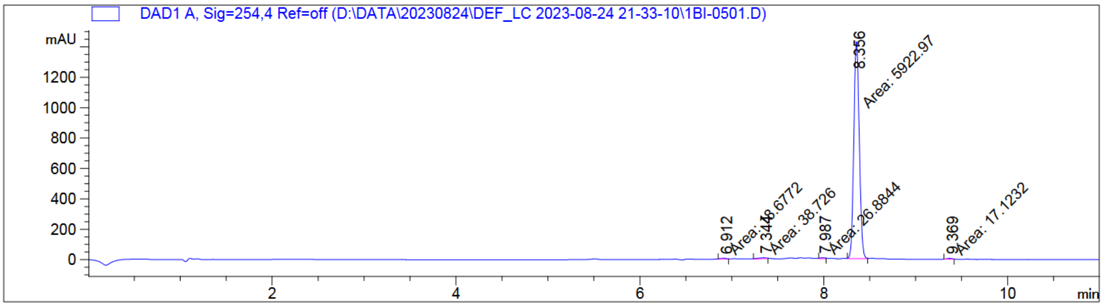


```
=====================================================================
                        Area Percent Report
=====================================================================

Sorted By             :      Signal
Multiplier:                  :       1.0000
Dilution:                    :       1.0000
Use Multiplier & Dilution Factor with ISTDs


Signal 1: DAD1 A, Sig=254,4 Ref=off

Peak RetTime Type  Width     Area      Height     Area
  #   [min]        [min]   [mAU*s]     [mAU]        %
----|-------|----|-------|----------|----------|--------|
   1   6.912 MM    0.0629   18.67718    4.94883   0.3100
   2   7.344 MM    0.1078   38.72599    5.98780   0.6428
   3   7.987 MM    0.0641   26.88441    6.98822   0.4463
   4   8.356 MM    0.0688 5922.96680 1435.06763  98.3167
   5   9.369 MM    0.0658   17.12322    4.33613   0.2842
```

The purity of 8 (98.81%).

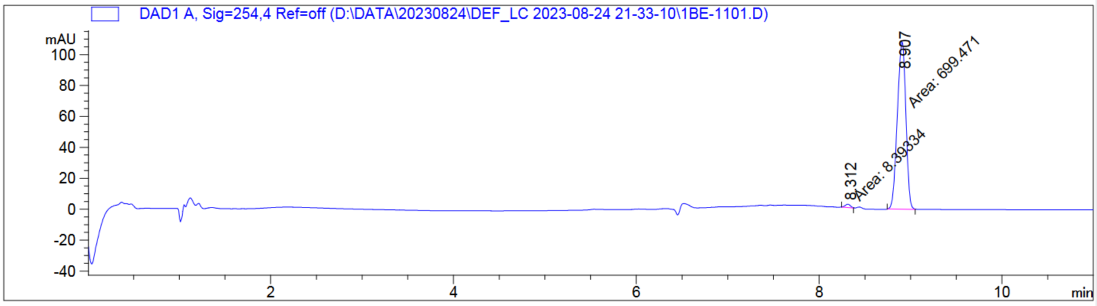


```
=====================================================================
                        Area Percent Report
=====================================================================

Sorted By             :      Signal
Multiplier:                  :       1.0000
Dilution:                    :       1.0000
Use Multiplier & Dilution Factor with ISTDs


Signal 1: DAD1 A, Sig=254,4 Ref=off

Peak RetTime Type  Width     Area      Height     Area
  #   [min]        [min]   [mAU*s]     [mAU]        %
----|-------|----|-------|----------|----------|--------|
   1   8.312 MM    0.0662    8.39334    2.11206   1.1857
   2   8.907 MM    0.1075  699.47131  108.47315  98.8143
```

The purity of 9 (98.62%).

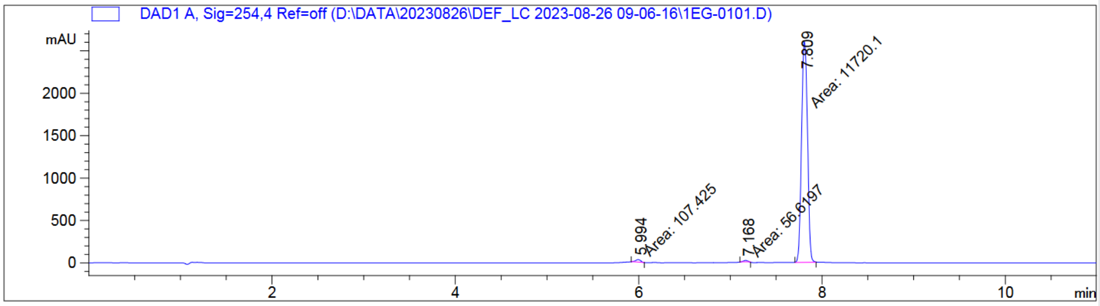


```
=====================================================================
                        Area Percent Report
=====================================================================

Sorted By             :      Signal
Multiplier:                  :      1.0000
Dilution:                    :      1.0000
Use Multiplier & Dilution Factor with ISTDs


Signal 1: DAD1 A, Sig=254,4 Ref=off

Peak RetTime Type  Width     Area       Height      Area
  #   [min]        [min]   [mAU*s]      [mAU]        %
----|-------|----|-------|----------|----------|--------|
   1   5.994 MM    0.0621  107.42525   28.82289   0.9039
   2   7.168 MM    0.0544   56.61969   17.33456   0.4764
   3   7.809 MM    0.0749 1.17201e4  2609.30054  98.6196
```

The purity of 10 (99.63%).

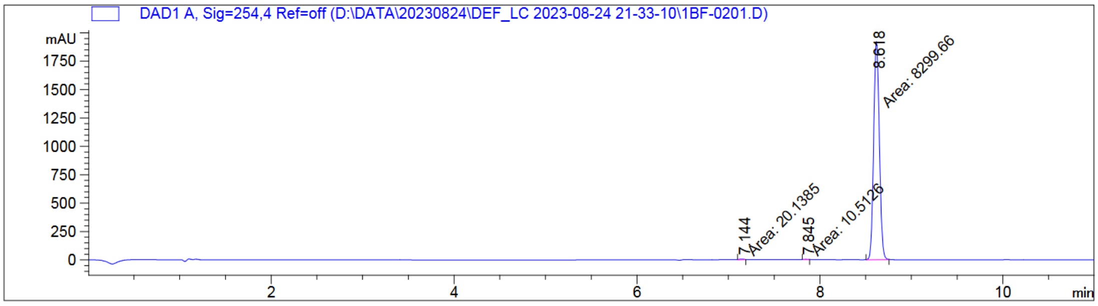


```
=====================================================================
                        Area Percent Report
=====================================================================

Sorted By             :      Signal
Multiplier:                  :      1.0000
Dilution:                    :      1.0000
Use Multiplier & Dilution Factor with ISTDs


Signal 1: DAD1 A, Sig=254,4 Ref=off

Peak RetTime Type  Width     Area       Height      Area
  #   [min]        [min]   [mAU*s]      [mAU]        %
----|-------|----|-------|----------|----------|--------|
   1   7.144 MM    0.0639   20.13852    5.24906   0.2417
   2   7.845 MM    0.0599   10.51259    2.92369   0.1262
   3   8.618 MM    0.0718 8299.65820 1925.66052  99.6321
```

The purity of 11 (99.14%).

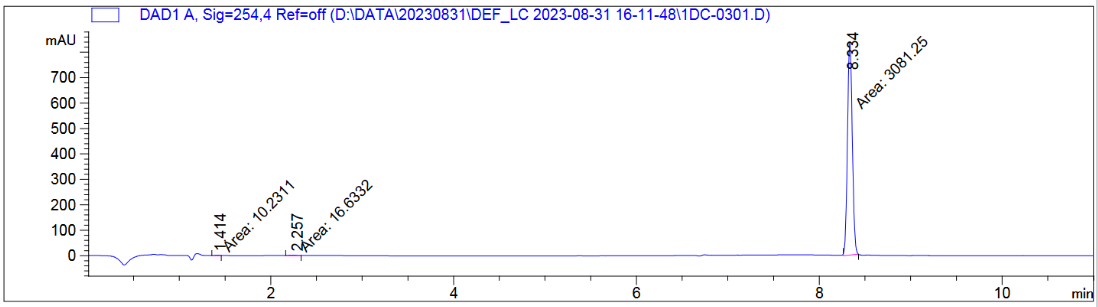


```
=====================================================================
                         Area Percent Report
=====================================================================

Sorted By             :      Signal
Multiplier:                  :      1.0000
Dilution:                    :      1.0000
Use Multiplier & Dilution Factor with ISTDs


Signal 1: DAD1 A, Sig=254,4 Ref=off

Peak RetTime Type  Width     Area      Height     Area
  #   [min]        [min]   [mAU*s]     [mAU]        %
----|-------|----|-------|----------|----------|--------|
   1   1.414 MM    0.0891   10.23112    1.91369   0.3292
   2   2.257 MM    0.1629   16.63325    1.70195   0.5352
   3   8.334 MM    0.0613 3081.25000  838.32788  99.1357
```

The purity of 12 (99.28%).

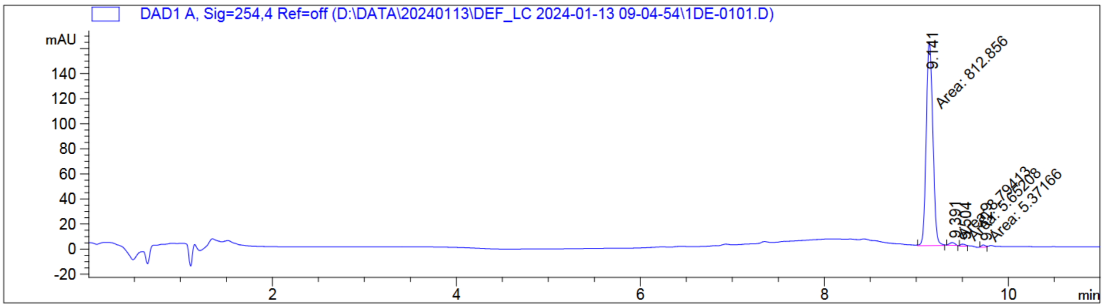


```
=====================================================================
                         Area Percent Report
=====================================================================

Sorted By             :      Signal
Multiplier:                  :      1.0000
Dilution:                    :      1.0000
Use Multiplier & Dilution Factor with ISTDs


Signal 1: DAD1 A, Sig=254,4 Ref=off

Peak RetTime Type  Width     Area      Height     Area
  #   [min]        [min]   [mAU*s]     [mAU]        %
----|-------|----|-------|----------|----------|--------|
   1   9.141 MM    0.0830  812.85559  163.29579  97.6200
   2   9.391 MM    0.0650    8.79413    2.25507   1.0561
   3   9.504 MM    0.0609    5.65208    1.54569   0.6788
   4   9.729 MM    0.0533    5.37166    1.68086   0.6451
```

The purity of 13 (99.31%).

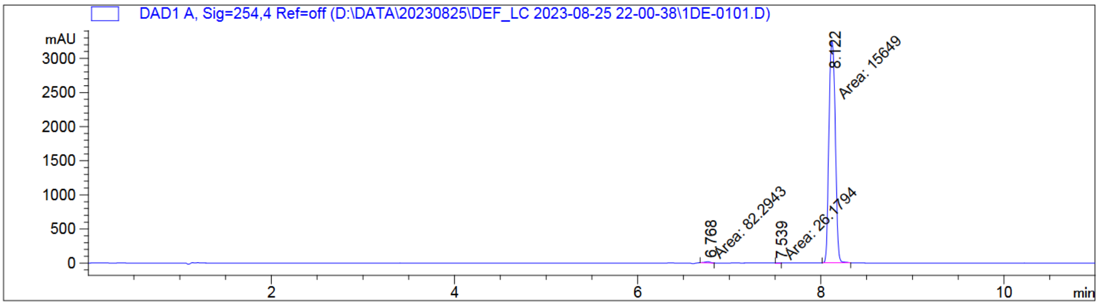


```
=====================================================================
                         Area Percent Report
=====================================================================

Sorted By             :      Signal
Multiplier:                   :       1.0000
Dilution:                     :       1.0000
Use Multiplier & Dilution Factor with ISTDs


Signal 1: DAD1 A, Sig=254,4 Ref=off
```

| Peak # | RetTime [min] | Type | Width [min] | Area [mAU*s] | Height [mAU] | Area % |
|---|---|---|---|---|---|---|
| 1 | 6.768 | MM | 0.0805 | 82.29435 | 17.04542 | 0.5223 |
| 2 | 7.539 | MM | 0.0646 | 26.17938 | 6.74938 | 0.1661 |
| 3 | 8.122 | MM | 0.0804 | 1.56490e4 | 3245.95264 | 99.3116 |

The purity of 14 (99.70%).

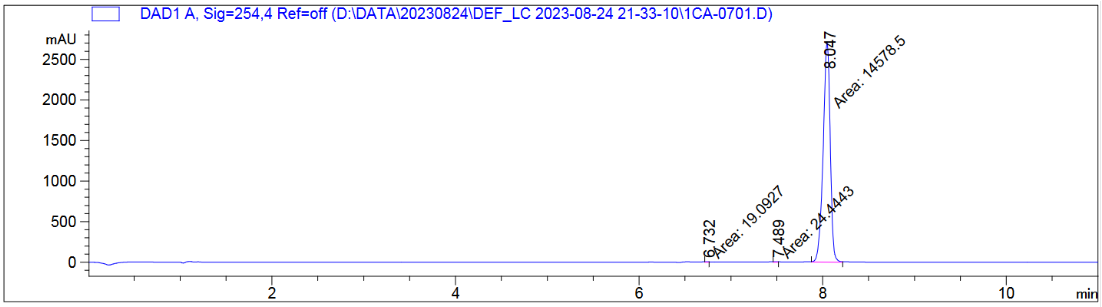


```
=====================================================================
                         Area Percent Report
=====================================================================

Sorted By             :      Signal
Multiplier:                   :       1.0000
Dilution:                     :       1.0000
Use Multiplier & Dilution Factor with ISTDs


Signal 1: DAD1 A, Sig=254,4 Ref=off
```

| Peak # | RetTime [min] | Type | Width [min] | Area [mAU*s] | Height [mAU] | Area % |
|---|---|---|---|---|---|---|
| 1 | 6.732 | MM | 0.0414 | 19.09267 | 7.67950 | 0.1306 |
| 2 | 7.489 | MM | 0.0530 | 24.44431 | 7.68521 | 0.1672 |
| 3 | 8.047 | MM | 0.0893 | 1.45785e4 | 2719.61670 | 99.7023 |

The purity of 15 (98.83%).

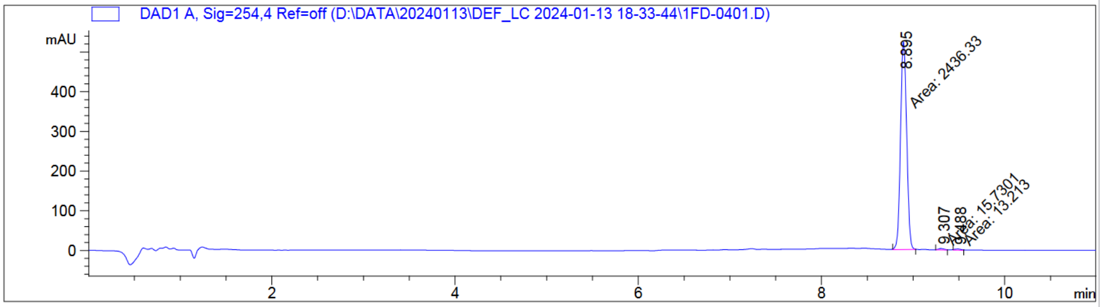


==================================================================

Area Percent Report

==================================================================

Sorted By : Signal
Multiplier: : 1.0000
Dilution: : 1.0000
Use Multiplier & Dilution Factor with ISTDs

Signal 1: DAD1 A, Sig=254,4 Ref=off

| Peak # | RetTime [min] | Type | Width [min] | Area [mAU*s] | Height [mAU] | Area % |
|---|---|---|---|---|---|---|
| 1 | 8.895 | MM | 0.0774 | 2436.32959 | 524.37195 | 98.8260 |
| 2 | 9.307 | MM | 0.0769 | 15.73011 | 3.41085 | 0.6381 |
| 3 | 9.488 | MM | 0.0809 | 13.21303 | 2.72341 | 0.5360 |

The purity of 16 (99.13%).

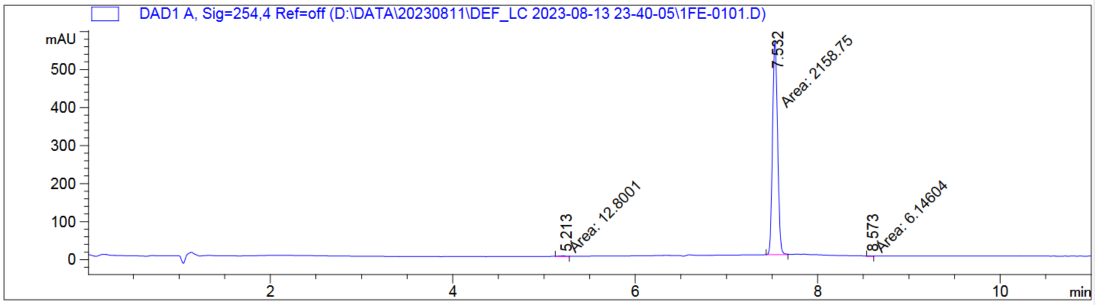


==================================================================

Area Percent Report

==================================================================

Sorted By : Signal
Multiplier: : 1.0000
Dilution: : 1.0000
Use Multiplier & Dilution Factor with ISTDs

Signal 1: DAD1 A, Sig=254,4 Ref=off

| Peak # | RetTime [min] | Type | Width [min] | Area [mAU*s] | Height [mAU] | Area % |
|---|---|---|---|---|---|---|
| 1 | 5.213 | MM | 0.1081 | 12.80010 | 1.97285 | 0.5878 |
| 2 | 7.532 | MM | 0.0639 | 2158.75171 | 562.64227 | 99.1300 |
| 3 | 8.573 | MM | 0.0730 | 6.14604 | 1.40389 | 0.2822 |

The purity of 17a (99.56%).

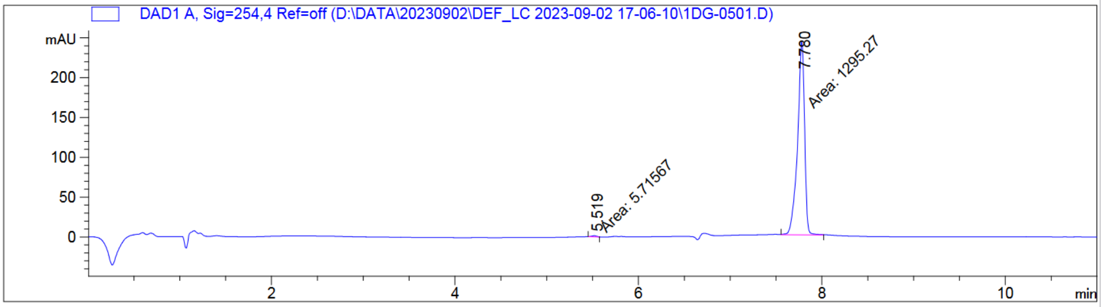


```
========================================================================
                         Area Percent Report
========================================================================

Sorted By              :      Signal
Multiplier:                    :       1.0000
Dilution:                      :       1.0000
Use Multiplier & Dilution Factor with ISTDs


Signal 1: DAD1 A, Sig=254,4 Ref=off

Peak RetTime Type  Width     Area      Height     Area
  #   [min]        [min]   [mAU*s]     [mAU]       %
----|-------|----|-------|----------|----------|--------|
   1   5.519 MM    0.0671    5.71567    1.41955   0.4393
   2   7.780 MM    0.0888 1295.27478  243.02625  99.5607
```

The purity of 17b (98.17%).

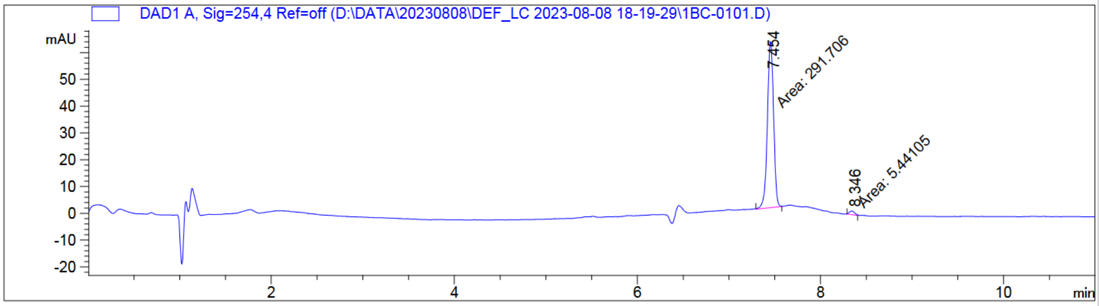


```
========================================================================
                         Area Percent Report
========================================================================

Sorted By              :      Signal
Multiplier:                    :       1.0000
Dilution:                      :       1.0000
Use Multiplier & Dilution Factor with ISTDs


Signal 1: DAD1 A, Sig=254,4 Ref=off

Peak RetTime Type  Width     Area      Height     Area
  #   [min]        [min]   [mAU*s]     [mAU]       %
----|-------|----|-------|----------|----------|--------|
   1   7.454 MM    0.0781  291.70584   62.23654  98.1689
   2   8.346 MM    0.0668    5.44105    1.35740   1.8311
```

The purity of 17c (98.16%).

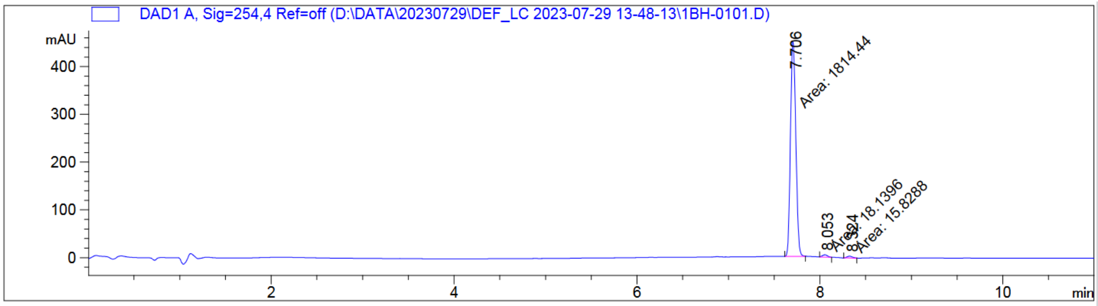


```
=====================================================================
                         Area Percent Report
=====================================================================

Sorted By             :      Signal
Multiplier:                   :      1.0000
Dilution:                     :      1.0000
Use Multiplier & Dilution Factor with ISTDs


Signal 1: DAD1 A, Sig=254,4 Ref=off

Peak RetTime Type  Width      Area      Height     Area
  #   [min]        [min]    [mAU*s]     [mAU]       %
----|-------|----|-------|----------|----------|--------|
   1   7.706 MM    0.0670 1814.43970  451.40579  98.1623
   2   8.053 MM    0.0642   18.13964    4.70734   0.9814
   3   8.324 MM    0.0698   15.82877    3.77922   0.8563
```

The purity of 17d (97.57%).

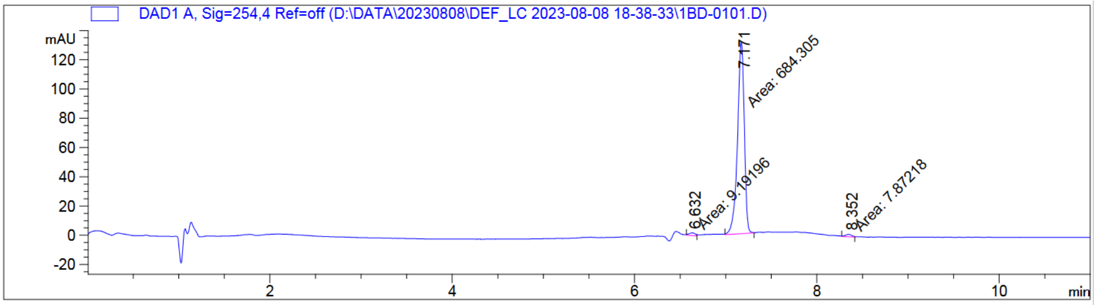


```
=====================================================================
                        Area Percent Report
=====================================================================

Sorted By             :      Signal
Multiplier:                   :      1.0000
Dilution:                     :      1.0000
Use Multiplier & Dilution Factor with ISTDs


Signal 1: DAD1 A, Sig=254,4 Ref=off

Peak RetTime Type  Width     Area      Height     Area
  #   [min]        [min]   [mAU*s]     [mAU]       %
----|-------|----|-------|----------|----------|--------|
   1   6.632 MM    0.0834    9.19196    1.83705   1.3106
   2   7.171 MM    0.0866  684.30469  131.66336  97.5670
   3   8.352 MM    0.0801    7.87218    1.63776   1.1224
```

The purity of 18a (99.27%).

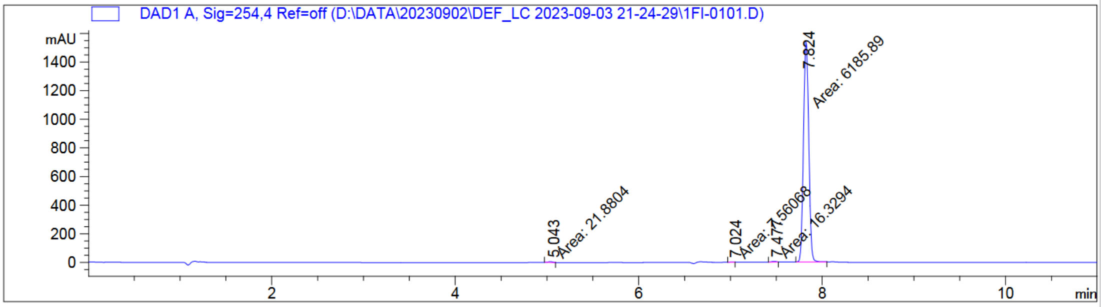


```
=====================================================================
                        Area Percent Report
=====================================================================

Sorted By             :      Signal
Multiplier:                   :      1.0000
Dilution:                     :      1.0000
Use Multiplier & Dilution Factor with ISTDs


Signal 1: DAD1 A, Sig=254,4 Ref=off

Peak RetTime Type  Width     Area      Height     Area
  #   [min]        [min]   [mAU*s]     [mAU]       %
----|-------|----|-------|----------|----------|--------|
   1   5.043 MM    0.0650   21.88040    5.61450   0.3511
   2   7.024 MM    0.0564    7.56068    2.23332   0.1213
   3   7.477 MM    0.0685   16.32936    3.97378   0.2620
   4   7.824 MM    0.0667 6185.89014 1544.71887  99.2655
```

The purity of 18b (98.37%).

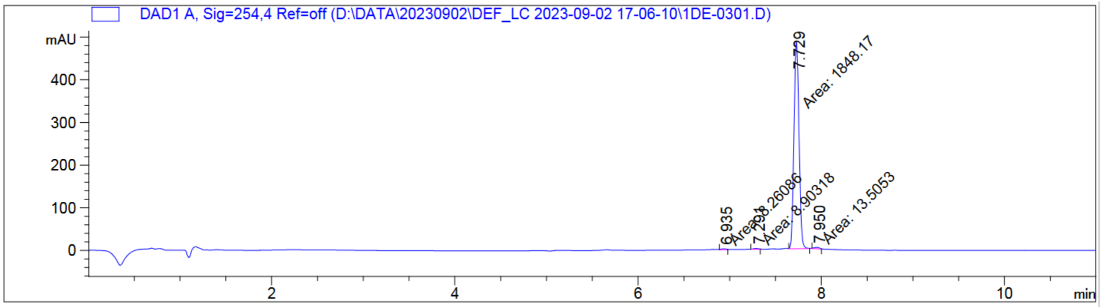


```
=====================================================================
                          Area Percent Report
=====================================================================

Sorted By             :      Signal
Multiplier:                   :      1.0000
Dilution:                     :      1.0000
Use Multiplier & Dilution Factor with ISTDs


Signal 1: DAD1 A, Sig=254,4 Ref=off

Peak RetTime Type  Width     Area      Height     Area
  #   [min]        [min]   [mAU*s]     [mAU]        %
----|-------|----|-------|----------|----------|--------|
   1   6.935 MM    0.0626    8.26086    2.20095   0.4397
   2   7.291 MM    0.0680    8.90318    2.18263   0.4739
   3   7.729 MM    0.0634 1848.17419  485.86865  98.3676
   4   7.950 MM    0.0651   13.50528    3.45881   0.7188
```

The purity of 19 (96.17%).

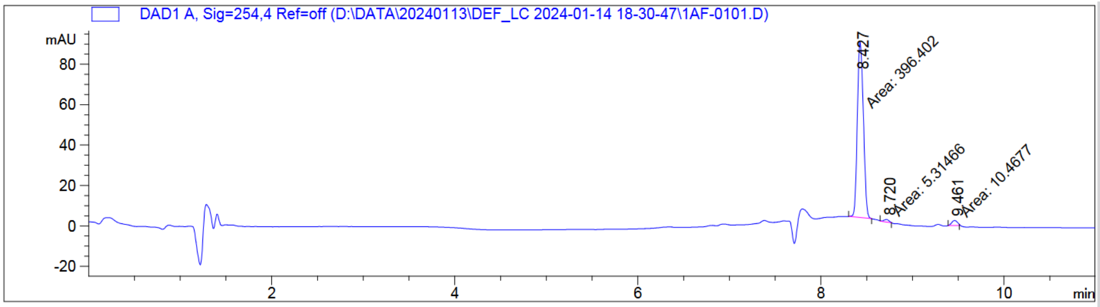


==================================================================

Area Percent Report

==================================================================

Sorted By : Signal
Multiplier: : 1.0000
Dilution: : 1.0000
Use Multiplier & Dilution Factor with ISTDs

Signal 1: DAD1 A, Sig=254,4 Ref=off

| Peak # | RetTime [min] | Type | Width [min] | Area [mAU*s] | Height [mAU] | Area % |
|---|---|---|---|---|---|---|
| 1 | 8.427 | MM | 0.0760 | 396.40228 | 86.96585 | 96.1711 |
| 2 | 8.720 | MM | 0.0683 | 5.31466 | 1.29662 | 1.2894 |
| 3 | 9.461 | MM | 0.0719 | 10.46768 | 2.42648 | 2.5396 |

The purity of 20 (97.06%).

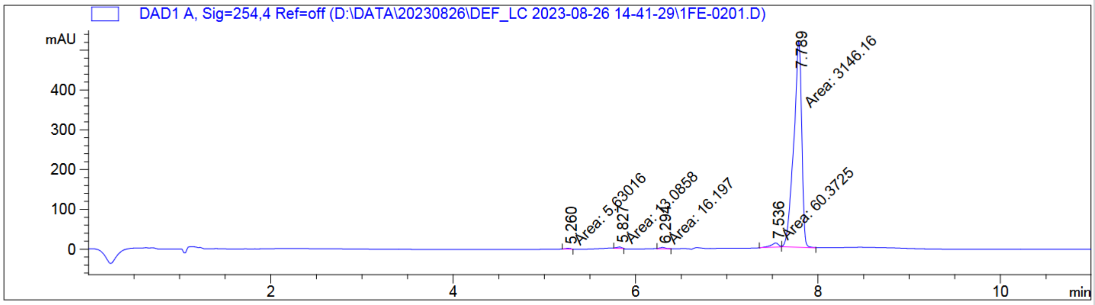


==================================================================

Area Percent Report

==================================================================

Sorted By : Signal
Multiplier: : 1.0000
Dilution: : 1.0000
Use Multiplier & Dilution Factor with ISTDs

Signal 1: DAD1 A, Sig=254,4 Ref=off

| Peak # | RetTime [min] | Type | Width [min] | Area [mAU*s] | Height [mAU] | Area % |
|---|---|---|---|---|---|---|
| 1 | 5.260 | MM | 0.0510 | 5.63016 | 1.84126 | 0.1737 |
| 2 | 5.827 | MM | 0.0612 | 13.08579 | 3.56169 | 0.4037 |
| 3 | 6.294 | MM | 0.0773 | 16.19702 | 3.49317 | 0.4997 |
| 4 | 7.536 | MM | 0.0950 | 60.37251 | 10.59505 | 1.8625 |
| 5 | 7.789 | MM | 0.1011 | 3146.16162 | 518.66309 | 97.0604 |

The purity of 21 (96.34%).

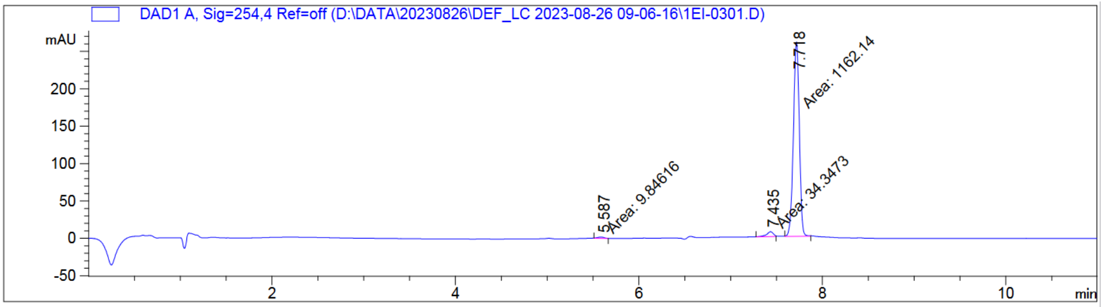


==================================================================

Area Percent Report

==================================================================

Sorted By : Signal
Multiplier: : 1.0000
Dilution: : 1.0000
Use Multiplier & Dilution Factor with ISTDs

Signal 1: DAD1 A, Sig=254,4 Ref=off

| Peak # | RetTime [min] | Type | Width [min] | Area [mAU*s] | Height [mAU] | Area % |
|---|---|---|---|---|---|---|
| 1 | 5.587 | MM | 0.0890 | 9.84616 | 1.84455 | 0.8162 |
| 2 | 7.435 | MM | 0.0867 | 34.34732 | 6.59941 | 2.8472 |
| 3 | 7.718 | MM | 0.0741 | 1162.14490 | 261.56580 | 96.3366 |

The purity of 22 (97.34%).

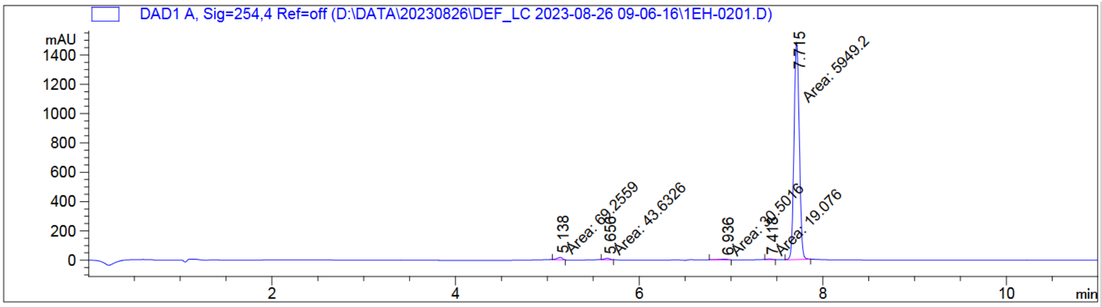


==================================================================

Area Percent Report

==================================================================

Sorted By : Signal
Multiplier: : 1.0000
Dilution: : 1.0000
Use Multiplier & Dilution Factor with ISTDs

Signal 1: DAD1 A, Sig=254,4 Ref=off

| Peak # | RetTime [min] | Type | Width [min] | Area [mAU*s] | Height [mAU] | Area % |
|---|---|---|---|---|---|---|
| 1 | 5.138 | MM | 0.0697 | 69.25591 | 16.55727 | 1.1332 |
| 2 | 5.656 | MM | 0.0718 | 43.63262 | 10.13262 | 0.7139 |
| 3 | 6.936 | MM | 0.1035 | 30.50156 | 4.91332 | 0.4991 |
| 4 | 7.418 | MM | 0.0645 | 19.07600 | 4.92889 | 0.3121 |
| 5 | 7.715 | MM | 0.0666 | 5949.20410 | 1488.62720 | 97.3417 |

The purity of 23 (99.29%).

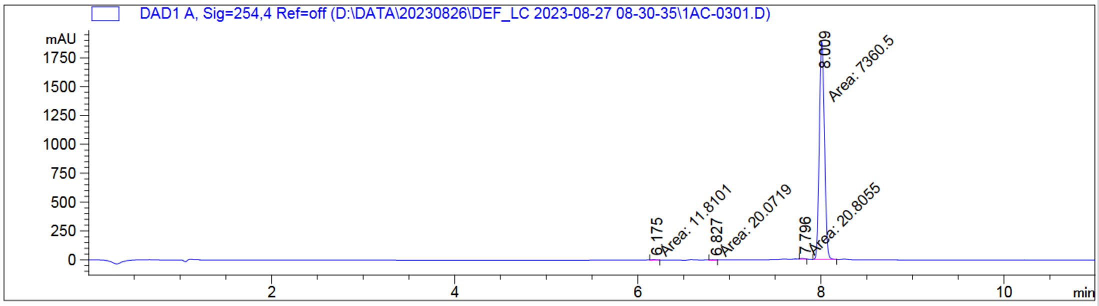


=====================================================================

Area Percent Report

=====================================================================

Sorted By : Signal
Multiplier: : 1.0000
Dilution: : 1.0000
Use Multiplier & Dilution Factor with ISTDs

Signal 1: DAD1 A, Sig=254,4 Ref=off

| Peak # | RetTime [min] | Type | Width [min] | Area [mAU*s] | Height [mAU] | Area % |
|---|---|---|---|---|---|---|
| 1 | 6.175 | MM | 0.0594 | 11.81011 | 3.31183 | 0.1593 |
| 2 | 6.827 | MM | 0.0823 | 20.07194 | 4.06349 | 0.2708 |
| 3 | 7.796 | MM | 0.0543 | 20.80546 | 6.38101 | 0.2807 |
| 4 | 8.009 | MM | 0.0647 | 7360.50244 | 1896.37988 | 99.2893 |

The purity of 24 (99.12%).

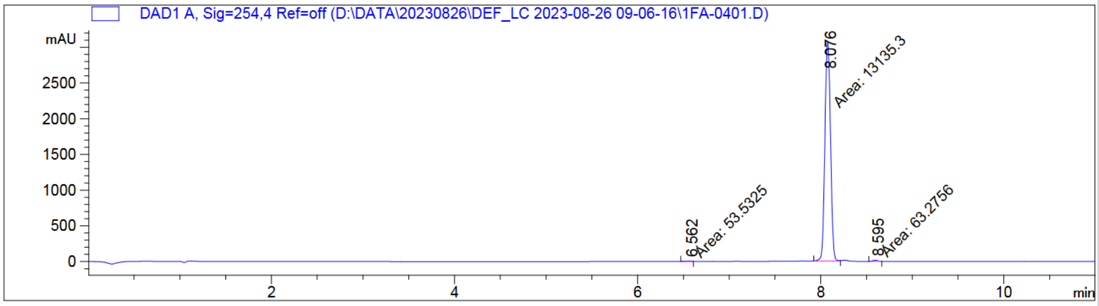


=====================================================================

Area Percent Report

=====================================================================

Sorted By : Signal
Multiplier: : 1.0000
Dilution: : 1.0000
Use Multiplier & Dilution Factor with ISTDs

Signal 1: DAD1 A, Sig=254,4 Ref=off

| Peak # | RetTime [min] | Type | Width [min] | Area [mAU*s] | Height [mAU] | Area % |
|---|---|---|---|---|---|---|
| 1 | 6.562 | MM | 0.0937 | 53.53246 | 9.51780 | 0.4040 |
| 2 | 8.076 | MM | 0.0711 | 1.31353e4 | 3079.90869 | 99.1186 |
| 3 | 8.595 | MM | 0.0693 | 63.27562 | 15.22856 | 0.4775 |

The purity of 25 (97.33%).

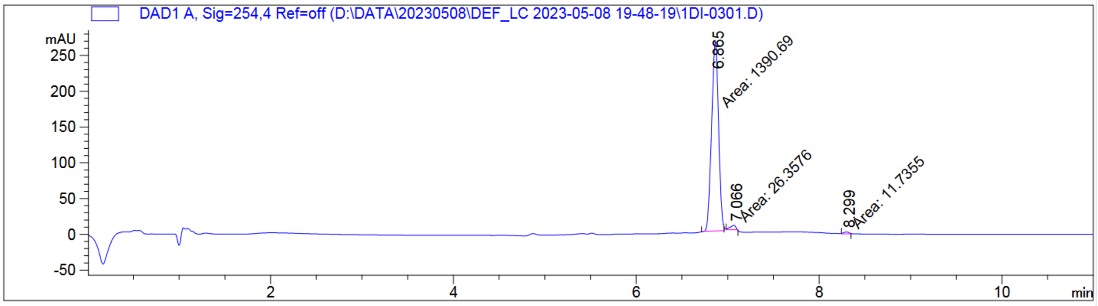


Area Percent Report

Sorted By : Signal
Multiplier: : 1.0000
Dilution: : 1.0000
Use Multiplier & Dilution Factor with ISTDs

Signal 1: DAD1 A, Sig=254,4 Ref=off

| Peak # | RetTime [min] | Type | Width [min] | Area [mAU*s] | Height [mAU] | Area % |
|---|---|---|---|---|---|---|
| 1 | 6.865 | MM | 0.0874 | 1390.68677 | 265.15381 | 97.3339 |
| 2 | 7.066 | MM | 0.0703 | 26.35763 | 6.24821 | 1.8448 |
| 3 | 8.299 | MM | 0.0697 | 11.73546 | 2.80455 | 0.8214 |

The purity of 26 (99.16%).

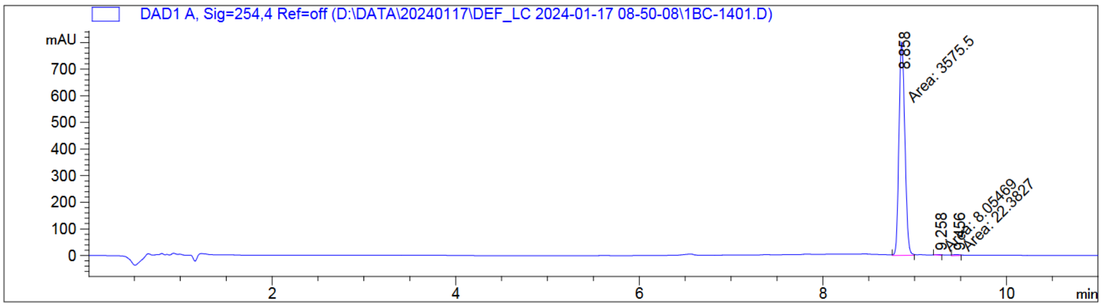


Area Percent Report

Sorted By : Signal
Multiplier: : 1.0000
Dilution: : 1.0000
Use Multiplier & Dilution Factor with ISTDs

Signal 1: DAD1 A, Sig=254,4 Ref=off

| Peak # | RetTime [min] | Type | Width [min] | Area [mAU*s] | Height [mAU] | Area % |
|---|---|---|---|---|---|---|
| 1 | 8.858 | MM | 0.0742 | 3575.50269 | 803.05969 | 99.1559 |
| 2 | 9.258 | MM | 0.0700 | 8.05469 | 1.91770 | 0.2234 |
| 3 | 9.456 | MM | 0.0856 | 22.38268 | 4.35875 | 0.6207 |

The purity of 27 (96.22%).

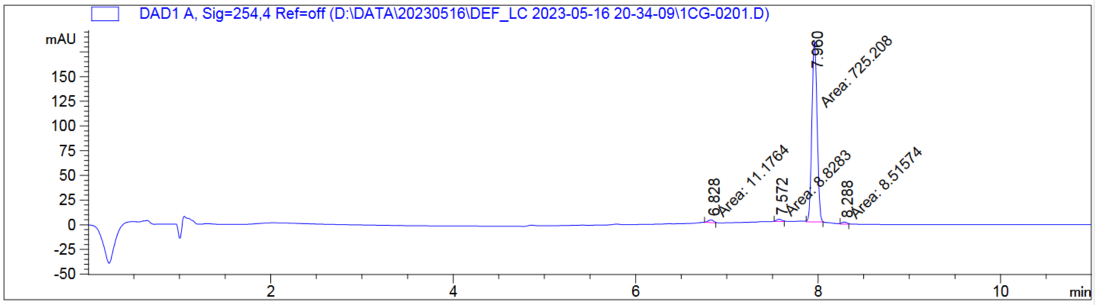


```
=====================================================================
                         Area Percent Report
=====================================================================

Sorted By             :      Signal
Multiplier:                  :       1.0000
Dilution:                    :       1.0000
Use Multiplier & Dilution Factor with ISTDs


Signal 1: DAD1 A, Sig=254,4 Ref=off

Peak RetTime Type  Width     Area      Height     Area
  #   [min]        [min]   [mAU*s]     [mAU]       %
----|-------|----|-------|----------|----------|--------|
   1   6.828 MM    0.0693   11.17641    2.68633   1.4828
   2   7.572 MM    0.0676    8.82830    2.17798   1.1713
   3   7.960 MM    0.0659  725.20789  183.27254  96.2161
   4   8.288 MM    0.0654    8.51574    2.17109   1.1298
```
